# Environmental outcomes of the US Renewable Fuel Standard

**DOI:** 10.1073/pnas.2101084119

**Published:** 2022-02-14

**Authors:** Tyler J. Lark, Nathan P. Hendricks, Aaron Smith, Nicholas Pates, Seth A. Spawn-Lee, Matthew Bougie, Eric G. Booth, Christopher J. Kucharik, Holly K. Gibbs

**Affiliations:** ^a^Nelson Institute for Environmental Studies, University of Wisconsin–Madison, Madison, WI 53726;; ^b^Department of Energy (DOE) Great Lakes Bioenergy Research Center, University of Wisconsin–Madison, Madison, WI 53726;; ^c^Department of Agricultural Economics, Kansas State University, Manhattan, KS 66506;; ^d^Department of Agricultural and Resource Economics, University of California, Davis, CA 95616;; ^e^Department of Agricultural Economics, University of Kentucky, Lexington, KY 40546;; ^f^Department of Geography, University of Wisconsin–Madison, Madison, WI 53726;; ^g^Department of Agronomy, University of Wisconsin–Madison, Madison, WI 53706;; ^h^Civil & Environmental Engineering, University of Wisconsin–Madison, Madison, WI 53706

**Keywords:** biofuels, land use change, greenhouse gas emissions, water quality, environmental policy

## Abstract

The Renewable Fuel Standard (RFS) specifies the use of biofuels in the United States and thereby guides nearly half of all global biofuel production, yet outcomes of this keystone climate and environmental regulation remain unclear. Here we combine econometric analyses, land use observations, and biophysical models to estimate the realized effects of the RFS in aggregate and down to the scale of individual agricultural fields across the United States. We find that the RFS increased corn prices by 30% and the prices of other crops by 20%, which, in turn, expanded US corn cultivation by 2.8 Mha (8.7%) and total cropland by 2.1 Mha (2.4%) in the years following policy enactment (2008 to 2016). These changes increased annual nationwide fertilizer use by 3 to 8%, increased water quality degradants by 3 to 5%, and caused enough domestic land use change emissions such that the carbon intensity of corn ethanol produced under the RFS is no less than gasoline and likely at least 24% higher. These tradeoffs must be weighed alongside the benefits of biofuels as decision-makers consider the future of renewable energy policies and the potential for fuels like corn ethanol to meet climate mitigation goals.

## Significance

Biofuels are included in many proposed strategies to reduce anthropogenic greenhouse gas emissions and limit the magnitude of global warming. The US Renewable Fuel Standard is the world’s largest existing biofuel program, yet despite its prominence, there has been limited empirical assessment of the program’s environmental outcomes. Even without considering likely international land use effects, we find that the production of corn-based ethanol in the United States has failed to meet the policy’s own greenhouse gas emissions targets and negatively affected water quality, the area of land used for conservation, and other ecosystem processes. Our findings suggest that profound advances in technology and policy are still needed to achieve the intended environmental benefits of biofuel production and use.

Bioenergy is an essential component of most proposed pathways to reduce anthropogenic greenhouse gas (GHG) emissions and limit global warming to 1.5 or 2 °C by middle to late century (1–6). Liquid biofuels may contribute to bioenergy’s share of climate mitigation by displacing petroleum-based fuels with those generated from modern-day plants (7, 8). The GHG benefits of such substitution, however, are dependent on several factors including whether biofuel production invokes additional plant growth (9–12), the extent to which combusted plants (typically crops) are replaced in the food system (13–15), and the degree to which biofuel production directly and indirectly alters patterns of land use and management (2, 16–20). Because land use changes (LUCs) and other consequences induced by biofuels have the potential to cause significant novel GHG emissions and modify other ecosystem services and disservices (21–26), accurately estimating and accounting these outcomes is critical for the formation of effective climate and environmental policy (27–29).

The United States is the world leader in biofuel production by volume and generated 47% of global output over the last decade under the purview of its Renewable Fuel Standard (RFS) (30). First enacted in 2005 and greatly expanded in 2007, the RFS requires that biofuels be blended into the transportation fuel supply at annually increasing increments. Volume targets exist for several advanced biofuel types including biomass-based diesel and those made from cellulosic feedstocks. However, the vast majority (∼87%) of the mandate to date has been fulfilled by conventional renewable fuels, specifically corn grain ethanol (30, 31), such that the potential benefits of its more advanced fuel requirements have not yet materialized (32–34).

To comply with the policy’s GHG reduction goals, the RFS requires conventional renewable fuels to generate life cycle GHG savings of at least 20% relative to gasoline. Upon enactment, the policy’s regulatory analysis projected that life cycle emissions of corn ethanol production would just clear the 20% threshold by 2022, even when emissions from LUC were included (35). At the time, most LUC emissions were projected to occur internationally. Since the initial RFS policy-making, however, observations of widespread land conversion and resultant GHG emissions within the United States have also emerged (36–39).

Heightened demand for crops for use as biofuel feedstocks and the associated changes to landscapes may also engender broader environmental disservices upon ground and surface waters, soil resources, and other ecosystem components (40–44). The magnitudes of such effects are highly uncertain, however, as they ultimately depend upon unpredictable behaviors throughout the supply chain—from field to refinery—making it difficult to forecast impacts. As such, public policy-making and support for biofuels has needed to rely on widely varying projections of anticipated effects—a quandary that could potentially misguide strategies for climate change mitigation and environmental protection (27, 28, 45).

The RFS legislation contains several environmental safeguards to try to prevent perverse outcomes including periodic scientific review of the conservation impacts of the program and opportunities to adjust annual fuel volumes if the program creates severe environmental harm (31). Although the most recent program review identified that biofuels may in fact be contributing to land conversion and subsequent declines in water quality, these impacts have not been causally attributed to biofuels or the RFS (32). Likewise, volume requirements for specific fuel types have not been revised based on environmental performance (31). Given the United States’ leading role in biofuel production, understanding the outcomes of the RFS has direct ramifications not only for national environmental quality and global climate change but also for policy-making around the world as governments seek to modify or develop their own biofuel policies to meet climate and clean energy goals.

Here we assess the effects of the RFS on US land and water resources during the first 8 y of the policy’s implementation (2008 to 2016) by integrating econometric analyses with observed changes in agricultural land use and models of biophysical impacts. We analyze how demand from the RFS affected corn, soybean, and wheat prices and how these price shocks influenced the areas planted to specific crops and cropland overall. We then assess how these changes affected key environmental indicators including nitrate leaching, phosphorus runoff, soil erosion, and GHG emissions. For all estimates, we compare outcomes under the 2007 RFS to a business-as-usual (BAU) counterfactual scenario in which ethanol production satisfies only the volume required by the initial 2005 version of the policy, equivalent to the amount needed for reformulated gasoline under the 1990 Clean Air Act. We apply our models only domestically, such that any environmental effects that occur outside the United States would be additional.

Our analyses show a modest change in the use of US agricultural land for crop production due to the RFS, which led to sizable increases in associated environmental impacts including nitrate leaching, phosphorus runoff, and soil erosion. While improvements in production efficiency have likely reduced the carbon intensity of corn ethanol since inception of the RFS, the previously underestimated emissions from US land conversion attributable to the policy are enough to fully negate or even reverse any GHG advantages of the fuel relative to gasoline. Our findings thereby underscore the importance of including such LUCs and environmental effects when projecting and evaluating the performance of renewable fuels and associated policies.

## Results and Discussion

**Fig. 1.**
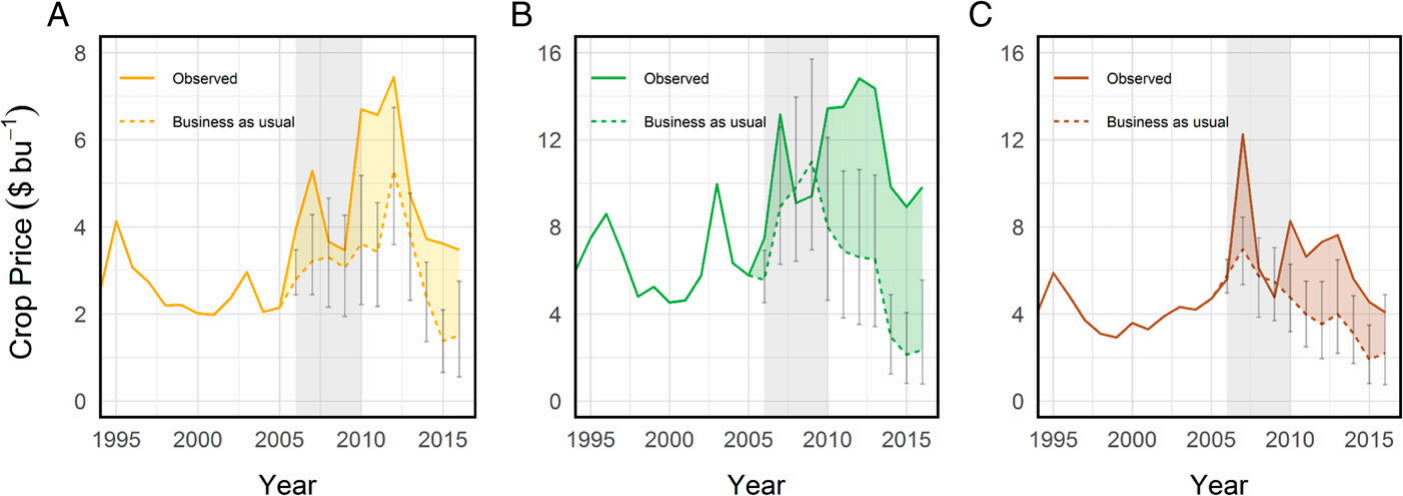
Observed and BAU estimates for crop prices. (*A*) Corn. (*B*) Soybeans. (*C*) Wheat. Vertical bars represent the 95% CIs for each BAU spot price. Each year denotes a crop year; e.g., 2006 is September 2006 to August 2007 for corn and soybeans and June 2006 to May 2007 for wheat. Averages for 2006 to 2010 (highlighted in gray) were used to derive the estimates in the text, although long-run persistent impacts were consistent with these results (46).

We found that the RFS stimulated 20.8 billion L (5.5 Bgal) of additional annual ethanol production, which requires nearly 1.3 billion bushels of corn after accounting for coproducts that can be fed to animals (46). This heightened demand led to persistent increases in corn prices of ∼31% (95% confidence interval [CI]: 5%, 70%) compared to BAU (Fig. 1). The increased demand for corn also spilled over onto other crops, increasing soybean prices by 19% [−8%, 72%] and wheat by 20% [2%, 60%] (*SI Appendix*, Table S1). These outcomes approximate the contribution of the RFS policy specifically, although other factors including changes in fuel blending economics that favored 10% ethanol as an octane source in gasoline (E10) may also have contributed (*SI Appendix*, *Supplementary Results for Price Impacts*).

**Fig. 2.**
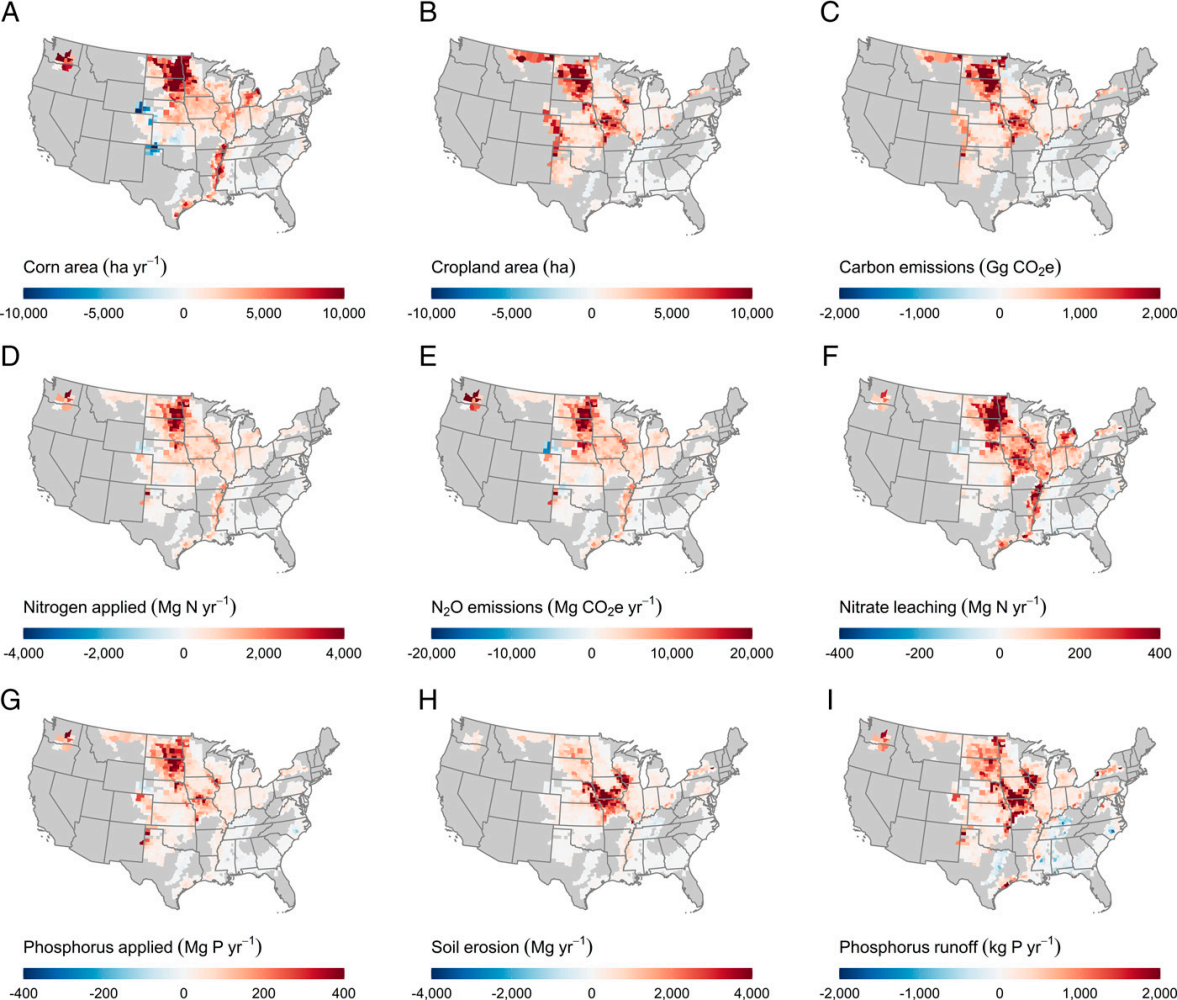
Changes due to the RFS. (*A*) Corn planted area. (*B*) Cropland area. (*C*) Carbon emissions. (*D*) Nitrogen applications. (*E*) Nitrous oxide emissions. (*F*) Nitrate leaching. (*G*) Phosphorus applications. (*H*) Soil erosion. (*I*) Phosphorus runoff. Positive numbers indicate an increase due to the RFS. Field-level results were aggregated to the county level for enumeration and visualization.

The increase in corn prices relative to other crops increased the area planted to corn on existing cropland by an average of 2.8 Mha* per year [95% CI: 2.4, 3.1], which is an 8.7% increase attributable to the RFS. This additional area resulted from producers planting corn more frequently, including a 2.1 Mha [1.8, 2.3] increase in continuous corn production (i.e., sequential year cropping) and a 1.4 Mha [0.8, 1.9] increase in the area planted in rotation with other crops (*SI Appendix*, *Supplementary Results for Crop Rotations* and Fig. S1). Collectively, corn area increased most markedly in North and South Dakota, western Minnesota, and the Mississippi Alluvial Plain—regions where the amount of corn increased 50 to 100% due to the RFS (Fig. 2*A* and *SI Appendix*, Fig. S1).

Heightened commodity prices from the RFS also increased active cropland extent. We estimate that the RFS caused conversion of an additional 1.8 Mha [95% CI: 1.5, 2.1] of natural and seminatural areas to cropland between 2008 and 2016, or 26% more than would have otherwise likely occurred (*SI Appendix*, *Supplementary Results for Cropland Area* and Table S2). Higher prices also reduced cropland abandonment; less cropland was returned to grass or natural cover, either as pasture or through enrollment into the Conservation Reserve Program (CRP), a federal set-aside that pays farmers to reestablish perennial vegetation. We estimate that the RFS decreased abandonment by 0.4 Mha [0.1, 0.6], or 6% less abandonment than expected with BAU. Together these extensive changes produced a net increase in cropland area of 2.1 Mha [1.8, 2.5] relative to BAU, with the greatest increases occurring in the western portions of existing agricultural regions (Fig. 2*B* and *SI Appendix*, Fig. S2).

The combined changes in the intensity of corn production and extent of cropland caused 7.5% more reactive nitrogen (N) from synthetic fertilizer to be applied annually to the landscape (Table 1). This contributed to a 5.3% increase in nitrate (NO_3_^−^) leached annually from agricultural land due to the RFS. Such nitrate losses occurred through vertical seepage below the root zone, where nutrients are no longer accessible to crops, and have been implicated in widespread groundwater contamination throughout the United States with major public health consequences (47, 48). Leaching was highest in regions with high N inputs and coarse soil texture (Fig. 2*F* and *SI Appendix*, Figs. S3 and S4), with nearly two-thirds of the overall nitrate increase stemming from changes to crop rotations.

**Table 1.** Net changes due to the RFS

	Land use		GHG emissions		Environmental indicators				
	Corn area (Mha y^−1^)	Cropland area (Mha)	Nitrous oxide (TgCO_2_e y^−1^)	Ecosystem carbon (TgCO_2_e)	N applied (Gg-N y^−1^)	P applied (Gg-P y^−1^)	Nitrate leaching (Gg-N y^−1^)	P runoff (Mg-P y^−1^)	Soil erosion (Gg y^−1^)
Crop rotation Δ	2.8	—	2.8	—	480.0	21.3	87.1	203.2	222.9
95% CI lower limit	2.4	—	2.3	—	377.3	1.2	56.9	−27.7	11.6
95% CI upper limit	3.1	—	3.2	—	577.0	41.2	117.7	449.3	423.6
Cropland extent Δ	—	2.1	1.3	397.7	237.3	48.2	47.9	439.0	633.9
95% CI lower limit	—	1.8	1.0	313.3	190.5	38.4	33.5	273.6	485.9
95% CI upper limit	—	2.5	1.5	481.7	281.8	57.7	62.1	592.6	780.6
Combined total Δ	2.8	2.1	4.1	397.7	717.2	69.5	135.0	642.2	856.7
95% CI lower limit	2.4	1.8	3.5	313.3	626.7	58.9	111.6	476.9	697.6
95% CI upper limit	3.1	2.5	4.5	481.7	806.5	79.5	157.8	798.0	1,011.4
BAU baseline	31.7	88.4	48.6	—	9,545.5	1,986.4	2,535.5	19,939.3	18,038.7
%Δ from BAU	8.7%	2.4%	8.3%	—	7.5%	3.5%	5.3%	3.2%	4.7%
%Δ per BGY	1.6%	0.4%	1.5%	—	1.4%	0.6%	1.0%	0.6%	0.9%

%Δ from BAU = percent change from BAU; i.e., the incremental effect of the 2007 expansion of the RFS; %Δ per BGY = percent change per BGY increase in ethanol demand.

The RFS also increased total edge-of-field phosphorus (P) runoff by 3.2% (Fig. 2*I* and *SI Appendix*, Figs. S5 and S6). This change was driven by a 3.5% increase in total P applications (Fig. 2*G*) and a 4.7% increase in soil erosion (Fig. 2*H*), which transports dissolved and sediment-bound P to downstream surface waters, where it often causes eutrophication and harmful algal blooms (41, 47, 49). Erosion losses from crop fields can also degrade soil quality over time (50, 51), contribute to enhanced GHG emissions in waterways (52), and impair water quality and aquatic habitat (53, 54) including that of threatened and endangered species (55, 56).

Collectively, increased nitrate leaching, phosphorus runoff, and soil erosion from the RFS fall within the range of outcomes projected at its outset (41, 57, 58) and substantiate long-standing concerns about the policy’s environmental disservices. However, we find disproportionate effects and distinct spatial patterns from different pathways of land use response. Shifting crop rotations toward more corn increased N fertilizer applications and nitrate leaching by nearly twice that of cropland area changes, due largely to the high N requirements of corn relative to other crops. In contrast, erosion-driven P and soil losses from cropland area expansion were roughly two and three times greater, respectively, than those from increased corn planting—a difference that reflects substantially higher erodibility and P inputs of croplands relative to uncultivated land, particularly in the marginal, steeper-sloped areas that were converted (e.g., *SI Appendix*, Fig. S6*I*) (37, 59, 60).

Beyond its water quality effects, the RFS substantially increased on-site GHG emissions from cropping systems. We found that greater use of N fertilizer increased nitrous oxide (N_2_O) emissions by 8.3% or 4.1 Tg CO_2_e y^−1^ relative to BAU (Fig. 2*E*). Most of this (68%) can be attributed to intensified corn production on preexisting fields, where emissions increased by 5.7%, with the remainder emitted from the expanded croplands.

In addition to these annual fertilizer application emissions, degradation of ecosystem carbon (C) stocks from cropland expansion led to a substantial pulse of committed GHG emissions. These arise from clearing land for crop production and are typically realized over a period of roughly 30 y unless proactively mitigated (35, 61). We estimate emissions associated with RFS-induced conversion to cropland to be 320.4 Tg CO_2_e [95% CI: 250.5, 384.3], or ∼181 Mg CO_2_e ha^−1^.

Further, reduced rates of cropland retirement—through CRP enrollment or transition to pasture—has reduced C sequestration that would have otherwise resulted from perennial grassland reestablishment and recovery. We estimate this forgone sequestration at 77.3 Tg CO_2_e [95% CI: 30.8, 126.8], assuming that abandoned land would accumulate carbon for 15 y—the standard duration of a single CRP contract—after which its carbon fate becomes contingent upon subsequent management. Combined, the RFS-driven changes in cropland area between 2008 and 2016 caused a total net C flux of 397.7 Tg CO_2_e [313.3, 481.7] to the atmosphere (Fig. 2*C*).

Domestic LUC emissions spurred by the RFS undermine the GHG benefits of using ethanol as transportation fuel. Assuming 30-y amortization, ecosystem C emissions from the RFS-induced LUC equate to 637 g CO_2_e L^−1^ of increased annual ethanol production or an emissions intensity of 29.7 g CO_2_e MJ^−1^ (*SI Appendix*, Table S3). Including on-site annual nitrous oxide emissions from increased fertilizer application further increases these emissions to 831 g CO_2_e L^−1^ or 38.7 g CO_2_e MJ^−1^. These findings stand in stark contrast to the −3.8 g CO_2_e MJ^−1^ of domestic LUC emissions estimated by the RFS regulatory impact analysis (RIA) and surpass the 30.3 g CO_2_e MJ^−1^ estimated by the RIA for international LUC (35).

Substituting our empirically derived domestic emissions for those modeled in the RFS RIA would raise ethanol’s projected life cycle GHG emissions for 2022 to 115.7 g CO_2_e MJ^−1^—a value 24% above baseline gasoline (93.1 g CO_2_e MJ^−1^). The RIA estimate, however, includes improvements in feedstock and ethanol production efficiency that were projected to occur by 2022, such that the GHG intensity of ethanol produced at earlier time periods and over the life of the RFS to date is likely much higher [*SI Appendix*, *Supplementary Results for Greenhouse Gas* (*GHG*) *Emissions from Land Use Change* (*LUC*)].

**Fig. 3.**
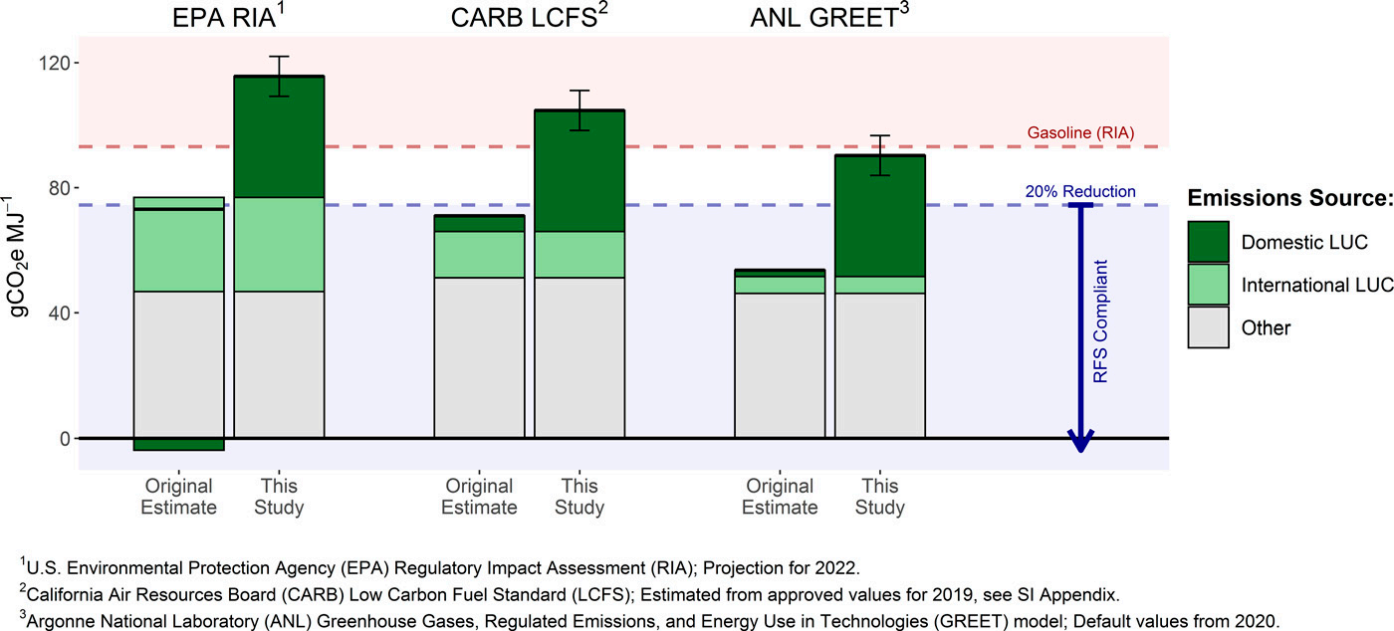
GHG emission intensities for corn ethanol with and without updated domestic LUC emissions. Original estimates reflect GHG intensities of corn ethanol according to the US EPA RIA [projection for 2022 (35)], California Air Resources Board (CARB)’s Low Carbon Fuel Standard (LCFS) [estimated from approved values for 2019 (62); *SI Appendix*], and Argonne National Laboratory (ANL)’s Greenhouse Gases, Regulated Emissions, and Energy Use in Technologies (GREET) model [default values for 2020 (63)]. Revised estimates (this study) replace the estimated domestic LUC emission from each source with those identified in this study. Our domestic LUC emissions estimate includes ecosystem carbon losses (including methane) from land conversion and on-site nitrous oxide emissions from additional fertilizer usage but excludes all other upstream and downstream emissions. Error bars represent 95% CIs for emissions from domestic LUC only (*SI Appendix*).

Incorporating the domestic LUC emissions from our analysis into other fuel program estimates similarly annuls or reverses the GHG advantages they calculate for ethanol relative to gasoline (Fig. 3 and Table 2). However, life cycle GHG emissions accounting requires consistent treatments and system boundaries across analyses (27, 64–66). As such, a full reanalysis, rather than the partial revisions we illustrate here, should be conducted to accurately assess ethanol’s carbon intensity relative to other fuels, particularly given the magnitude of domestic LUC emissions identified. For instance, we likely underestimate total domestic LUC impacts since we consider only the on-site ecosystem C and nitrous oxide emissions but do not account for additional emissions from increased fertilizer production (67) or from water quality–related increases in N, P, and sedimentation, which have been shown to augment GHG emissions in downstream waterways (52, 68, 69).

**Table 2.** GHG emissions intensities for LUC, total ethanol, and reference gasoline

	kg CO_2_e/mmBtu	g CO_2_e/MJ	% change from gasoline
LUC emissions			
This study, domestic	40.9	38.7	—
EPA RIA*, domestic	−4.0	−3.8	—
EPA RIA*, international	31.8	30.1	—
CARB LCFS^†^, combined	20.9	19.8	—
GREET^‡^, domestic	2.1	2.0	—
GREET^‡^, international	5.7	5.4	—
Total ethanol			
RIA*	77.2	73.2	−21.4%
RIA* + this study	122.1	115.7	24.3%
LCFS^†^	74.9	71.0	−23.7%
LCFS^†^ + this study	110.4	104.7	12.5%
GREET^‡^	56.6	53.6	−42.4%
GREET^‡^ + this study	95.3	90.3	−3.0%
Other			
RIA gasoline*	98.2	93.1	0.0%

*US EPA RIA; projection for 2022 (35).

^†^CARB LCFS; approved values for 2019 (62).

^‡^ANL GREET model; default values for 2020 (63).

Furthermore, we assess only the domestic (US) impacts of the RFS and expanded corn ethanol production. However, evidence of such effects reaffirms the likely presence of international LUC in response to the RFS (16, 19, 28, 70). As such, our results should be considered the lower bound for total GHG and other environmental impacts. We also limit our focus to select environmental outcomes but note that interconnected outcomes related to food systems (13, 14), human health (71), and the welfare of different groups of society (72) likely exist. For example, several assessments of the GHG implications of the RFS model a concomitant reduction in global food and feed consumption (13, 19).

Although we describe the incremental effects of the expanded RFS program, our findings are representative of the observed outcomes from corn ethanol development broadly, regardless of the cause. Our estimates imply that for every billion gallons per year (BGY) expansion of ethanol demand, we would expect a 5.6% increase in corn prices; 1.6 and 0.4% increases in the areas of US corn and cropland, respectively; and attendant increases in GHG emissions, nutrient pollution, and soil erosion (Table 1; %Δ per BGY). Our findings are also specific to corn ethanol and do not reflect advanced renewable fuels, which have lower production volumes and are required to meet stricter GHG reduction thresholds. To date, however, most RFS biofuel production has come from conventional corn ethanol, thereby missing much of the policy’s promised emissions savings and potential environmental benefits expected from more advanced feedstocks (2, 35, 73).

Despite the strong environmental tradeoffs under the RFS thus far, biofuels and bioenergy may play a key role in stabilizing atmospheric CO_2_ concentrations and holding global warming below 1.5 or 2 °C, particularly with continued advancements like carbon capture and storage (2, 4, 74–76) and increased productivity from perennial feedstocks grown on marginal lands (77–80). However, our findings confirm that contemporary corn ethanol production is unlikely to contribute to climate change mitigation. Given the current US dependence on this fuel, there remains an urgent need to continue the research, development, and shift toward more-advanced renewable fuels, improved transportation efficiency, and electrification (74, 81–83).

The United States is currently at a bioenergy crossroads. The RFS specifies biofuel volumes through 2022; absent legislative action, the Environmental Protection Agency (EPA) will determine volumes for subsequent years. If conventional biofuel volumes were to increase, it is likely that further increases in crop prices, LUC, and environmental impacts would ensue. Alternatively, a decrease in mandated volumes may have less effect, given the capital investment, established markets, and economic value of producing ethanol at existing levels. More broadly, any increases in demand for corn ethanol from nonfederal jurisdictions, including US states or trade partners like Canada and China, are likely to exacerbate the domestic land use and environmental outcomes identified here.

As policy-makers worldwide deliberate the future of biofuels, it is essential that they consider the full scope of the associated tradeoffs, weighing the GHG and other environmental externalities alongside each fuel’s benefits. By quantifying and attributing the outcomes of policy thus far, our findings provide fundamental evidence to guide this process and set realistic expectations for the contribution that current biofuel technologies can make toward climate mitigation and other environmental goals.

## Materials and Methods

We estimated the domestic environmental effects of the 2007 US RFS by linking a series of empirical and explanatory models. First, we estimated the impacts of the RFS on the prices of corn, soybeans, and wheat. We then simulated, via independent models, the responses of crop rotations and total cropland area to the changes in crop prices. Last, we quantified the associated environmental outcomes by employing models specific to water quality indicators, nitrous oxide emissions, and ecosystem carbon emissions and updated existing life cycle estimates of ethanol’s GHG intensity to reflect these findings.

Overall, our retrospective and purpose-built integrated assessment modeling framework has several advantages over previous projections and more generalized approaches. For example, 1) we utilize observed rather than predicted crop prices and land uses as a baseline factual scenario against which we compare our counterfactual scenario, thereby eliminating one (of the two) sets of assumptions, projections, and uncertainties required for assessment; 2) our estimates of the effects on crop prices and land use are based on empirical assessments of observed changes rather than partial or general equilibrium models that rely heavily on assumptions and prescribed parameters; 3) we use historic changes in crop prices, crop rotations, and cropland area to validate our econometric models’ predictions and show strong temporal and regional fits between projected and observed changes; and 4) we utilize field-level remote sensing data to detect the location of actual LUCs—rather than rely on assumptions about the type, location, and characteristics of converted lands—and use this information to more accurately estimate the environmental impacts of conversion. We also implemented several model-specific advances to improve the resolution, specificity, and performance of each individual component of analysis. We briefly describe each step of our analysis and its integration below and provide the full details in *SI Appendix*.

### Effects on Crop Prices.

We used a partially identified vector autoregression model to assess the effects of the RFS on US crop prices. Our approach closely follows that of Carter et al. (46) to account for competing shocks in demand due to changes in inventory, weather, and external markets and extends the work beyond corn to estimate the impacts of the RFS on soybean and wheat prices. We also incorporate the RFS policy as a persistent shock to agricultural markets rather than a transitory shock, whose price impacts are different (*SI Appendix*, *Estimating Effects on Crop Prices*).

In our analysis, we compare observed market prices to a counterfactual BAU scenario without the expanded 2007 RFS, where BAU ethanol production satisfies only the volume required by the initial 2005 RFS. This volume is roughly equivalent to the amount needed to meet oxygenate requirements for reformulated gasoline under the 1990 Clean Air Act. Our analysis therefore estimates the effects of the 2007 expansion of the RFS program above what would have otherwise likely occurred to meet demand for ethanol as an oxygenate after ethanol replaced methyl tert-butyl ether as the main oxygenate additive. As such, we assume the pre-2007 trend of increasing ethanol use would have continued without the expanded RFS, albeit at a slower rate.

Additional factors such as the Volumetric Ethanol Excise Tax Credit or improved cost competitiveness may have also contributed to ethanol’s growth. Our price effects are scalable, however, such that all land use, environmental quality, and GHG emissions that we report would remain the same on a per volume of ethanol basis, independent of the magnitude of demand change (within reasonable limit) or its source. Thus, our results also reflect observed outcomes from corn ethanol development in general, irrespective of whether such changes were driven by policy, markets, or other factors.

### Effects on Crop Rotations.

After modeling the price impacts of the RFS, we followed the approach of Pates and Hendricks to estimate how changes in crop prices affected crop rotations and the likelihood of planting continuous corn, continuous other crops, and corn–other crop rotations (49, 84, 85). We estimated a set of Markov transition models to separately estimate the probability of planting corn conditional on the crop planted in the prior season. One model estimates the probability of planting corn given corn was the previous crop, and the other estimates the probability of planting corn given a different crop was the previous crop. We then used these transition probabilities to estimate the probability of each crop rotation. To account for price response heterogeneity, we separately estimated these models for each major land resource area (MLRA) and major soil texture group. Advantages of our approach are that it explicitly accounts for the common practice of rotating crops and spatially heterogeneous responses to price across the country, as previous work shows that using aggregate data or ignoring price response heterogeneity can significantly bias estimates (84, 85). Furthermore, our model allows us to assess the location of environmental impacts as they relate to variation in price response.

To estimate the models, we built a spatiotemporal database using field boundary data (86–88) and associated information on annual crop type (89), soil properties (90), and climate (91) as well as crop futures and local spot prices (92). We then calculated the rotation probabilities for all fields greater than 15 acres that were in regions where 1) greater than 20% of the total area was cropland, 2) more than 10% of cropland acreage was planted to corn, and 3) greater than 50% of the cropland not planted to corn was planted to a crop for which prices were available (specifically wheat, soybeans, rice, and cotton). This set of criteria ensured adequate data were available to train each model, and our final sample included 3.6 million fields that accounted for 91.6% of corn acreage in the United States. Based upon results of the price impact modeling, we used a 30% persistent increase in the price of corn and 20% increases in the prices of soybeans and wheat to estimate, for each field, the change in probability of each rotation due to the RFS. We then derived area estimates using field sizes and summed the results across all fields and rotations (*SI Appendix*, *Estimating Effects on Crop Rotations*).

### Cropland Area Changes.

To assess LUCs at the extensive margin, we estimated the probability of transitioning between cropland and pasture or transitioning between cropland and CRP as a function of cropland, pasture, and CRP returns while controlling for soil and climate characteristics. We used a correlated random effects model to reduce concerns about endogeneity because the spatial variation in returns may be correlated with any omitted variables that affect land use transitions. Thus, our model is designed to better isolate the effect of changes in cropland returns on cropland transitions than other approaches that may confound differences in cropland returns across space with other unobserved factors that affect cropland transitions. We also account for the fact that land can only enter CRP when a sign-up is offered and can only exit CRP when the contract expires.

The model uses point-level land use transition data based on observed annual land use transitions in the National Resources Inventory (NRI) from 2000 to 2012. We then used the model to predict the change in transitions between 2008 and 2016 based on changes in prices (39). During this period, we predicted changes for 8 y, with the first transitions occurring between the 2008 and 2009 growing seasons. This approach may thus underestimate the total extensive land response to the RFS, as some land likely came into production prior to the 2009 growing season and after the 2016 growing season. In order to allow for geographic variation in the extensive response of land use to crop prices, we trained independent models for each of seven different land resource regions (LRRs) corresponding to aggregated MLRAs from the Natural Resources Conservation Service (*SI Appendix*, *Estimating Effects on Cropland Area*).

We then mapped observed LUC at field-level resolution during our study period following the general approach of Lark et al. (93) and using updated recommended practices (94, 95) to extend the analysis to 2008 to 2016 (37). These data were used to link the estimated extent of LUC associated with the RFS in each major LRR to specific locations of observed conversion for the purpose of enumerating environmental impacts. Thus, the high-resolution field data (37) were used only to identify the possible locations and characteristics of converted land, whereas the data from the NRI were used to estimate the magnitude of conversion and how much of it could be attributed to the RFS. This hybrid approach thereby combined the high certainty and long-term temporal coverage (prior to any RFS price signals) of the NRI data with the field-level specificity of the satellite-based land conversion observed during the study period (37, 94).

### Nutrient Application and Water Quality Impacts.

Rates of N and P application were developed using county-level estimates of fertilizer and manure application compiled by the US Geological Survey (96, 97), county-level estimates of area planted to specific crops from the Census of Agriculture (98), and typical fertilizer application ratios for the three major crop types (corn, soybeans, and wheat) from university extension publications (99). We then used these nutrient application estimates to drive a process-based agroecosystem model to simulate fluxes of water, energy, and nutrients across our study period for each crop rotation system across the United States as well as for each patch of converted land identified by the land transition model, following the approaches of Motew et al. (100) and Donner and Kucharik (41) (*SI Appendix*, *Estimating Water Quality Impacts*). To determine the impacts of the RFS from crop rotation changes, we multiplied the agroecosystem model outputs for each crop rotation by the change in its probability due to the RFS as determined via the econometric model described in the section* Effects on Crop Rotations*. To estimate the impact from cropland transitions due to the RFS, we assessed the relative differences in ecosystem outputs between cropland and noncropland for each individual transitioned parcel and multiplied each by the proportion of land transitioned within each LRR due to the RFS.

### GHG Emissions.

We modeled changes in N_2_O emissions from fertilizer applications using the nonlinear nitrogen effect model (NL-N-RR) of Gerber et al. (101). For each change in crop rotation or cropland area due to the RFS, we used the associated change in N application to estimate the corresponding change in N_2_O emissions. N_2_O emission estimates were converted to CO_2_e by assuming a 100-y global warming potential of 265 (102).

We estimated the ecosystem carbon emissions associated with RFS-related LUC using the methods of Spawn et al. (36). Carbon emissions from soil and biomass degradation associated with LUC were modeled for all observed conversions to cropland. In addition, a variant of the Spawn et al. model was created to assess forgone sequestration associated with reduced rates of abandonment. This model was structurally similar to that used for conversion to cropland but used a carbon response function (61) for conversion to grassland to estimate expected soil organic carbon accumulation over a 15-y period—the average length of a CRP contract. We thus assumed that any abandoned land would have been retired to the CRP and sequestering carbon for the duration of its contract. To attribute emissions to the RFS, we multiplied the combined net change in emissions from all observed LUC within a given LRR by the percentage of that region’s observed LUC that could be attributed to ethanol under the RFS.

To estimate emissions per liter of increased annual ethanol demand, we followed the approach of the EPA (35) and allocated total ecosystem carbon emissions over a 30-y period. We then added these amortized ecosystem carbon emissions to the annual nitrous oxide emissions from crop rotation and cropland area changes to estimate total annual emissions. We divided total annual emissions due to the RFS by the increased annual demand in ethanol estimated in our price impacts model and subsequently converted to emissions per unit of energy equivalent using a heating value of 21.46 MJ/L (35).

### Estimating Uncertainty.

We quantified uncertainty at multiple points of our causal analysis framework including the price impact analyses, the crop rotation and cropland transition analyses, and the environmental impact modeling (*SI Appendix*, *Estimating Uncertainty*). Except for the price impacts, we propagated the uncertainty results throughout the connected components—from the land use models through to all subsequent environmental outcomes. All results are presented in the main text as 95% CIs, reported as [lower limit (0.025 quantile), upper limit (0.975 quantile)].

## Supplementary Material

Supplementary File

## Data Availability

All national and regionally aggregated data are available in the main text and *SI Appendix*. All underlying field-level data aggregated to counties have been deposited in a permanent repository (https://doi.org/10.5281/zenodo.5794632). Code developed for and used in this study is available on GitHub (https://github.com/gibbs-lab-us/). All other study data are included in the article and *SI Appendix*. Previously published data were also used in this work (https://doi.org/10.5281/zenodo.3905242).

## Footnotes

*See *SI Appendix* results: Our model of key growing regions accounts for 91.6% of corn acres in the United States. If one assumes a similar response in the remaining unmodeled area, then the nationwide change is 3.0 Mha or 8.9% more than the amount expected without the RFS.
