# Supplementary material for: Environmental outcomes of the US Renewable Fuel Standard
Source: Proc Natl Acad Sci U S A. 2022 Feb 14;119(9):e2101084119. doi: 10.1073/pnas.2101084119 (PMC8892349; doi:10.1073/pnas.2101084119)
Supplement: Supplementary File [file pnas.2101084119.sapp.pdf]

PNAS
www.pnas.org

Supplementary Information for
Environmental Outcomes of the U.S. Renewable Fuel Standard

Tyler J. Lark[1*], Nathan P. Hendricks[2], Aaron Smith[3], Nicholas Pates[4], Seth A. Spawn-Lee[5], Matthew Bougie[1], Eric Booth[6], Christopher J. Kucharik[7], and Holly K. Gibbs[5]

*Corresponding author: Tyler J. Lark

Email: lark@wisc.edu

This PDF file includes:

Supplementary Text (SI Materials and Methods)
Supplementary Text (SI Results and Discussion)
Figures S1 to S35
Tables S1 to S23
SI References

Table of Contents

Supplementary Text (SI Materials and Methods) 3
Estimating effects on crop prices 3
Price model input – Estimated ethanol volumes 4
Price model development and estimation 6
Estimating effects on crop rotations 8
Crop rotation model input 8
Crop rotation model estimation 9
Estimating effects on cropland area 10
Cropland transitions model input 11
Cropland transitions model development 12
Cropland transitions model simulation. 14
Estimating specific locations of change 14
Estimating water quality impacts 15
Agroecosystem model input — Soil texture and topography 16
Agroecosystem model input — Historical land-use/land-cover 17
Agroecosystem model input — Fertilizer and manure N and P application rates 18
Agroecosystem model input — Irrigated extent 19
Estimating greenhouse gas (GHG) emissions from land use change (LUC) 19
Integrating models 20
Intensification – environmental effects of crop rotation changes 20
Extensification – environmental effects of cropland area changes 21
Estimating uncertainty 21
Supplementary Text (SI Results and Discussion) 24
Supplementary results for price impacts 24
Supplementary results for crop rotations 25
Supplementary results for water quality impacts from crop rotations 27
Supplementary results for cropland area 28
Supplementary results for water quality impacts of cropland area 30
Supplementary results for greenhouse gas (GHG) emissions from land use change (LUC) 31
Figs. S1-S35. 35
Tables S1-S23. 70
SI References 93

Supplementary Text (SI Materials and Methods)

We estimated the impacts of the 2007 U.S. Renewable Fuel Standard (RFS) on environmental outcomes by linking a series of empirical and explanatory models. First, we estimated the effects of the RFS on the prices of corn, soybeans, and wheat. We then simulated, using independent models, the responses of crop rotations and total cropland area to the changes in prices. Using those estimated changes, we quantified environmental outcomes, employing several models specific to nitrous oxide emissions, carbon emissions, nutrient losses, and water quality indicators. We describe the methods developed for each model component below, followed by the approaches used to integrate the models and estimate uncertainty.

<u>Estimating effects on crop prices</u>

We assessed the effects of the RFS on U.S. corn, soybean, and wheat prices by comparing observed market prices to a counterfactual business-as-usual scenario (BAU) without the expanded 2007 RFS, where BAU ethanol production satisfies only the volume required by the initial 2005 Renewable Fuel Standard — equivalent to the amount needed to meet standards for reformulated gasoline under the 1990 Clean Air Act. Our analysis therefore estimates the effects of the 2007 expansion of the RFS program above what would have otherwise likely occurred to meet demand for ethanol as an oxygenate. Prior to 2007, ethanol use was driven by the oxygenate requirement, which mandated beginning in the 1990s that oxygenate additives be blended into gasoline in regions prone to poor air quality. At first, methyl tert-butyl ether (MTBE) was used as the oxygenate additive, but when it was found to pollute waterways, ethanol replaced it. The initial RFS in 2005 essentially translated the oxygenate requirement into a volume mandate. See the section in Carter *et al.* (2017) titled Incremental Effect of RFS2 on Ethanol Production for further details (1).

Our approach closely follows that of Carter *et al.* to account for competing shocks in demand due to changes in inventory, weather, and external markets (1) and extends the work to estimate the impacts of the RFS on soybean and wheat prices. It also incorporates the policy as a persistent shock to agricultural markets rather than a transitory shock, whose price impacts are different. Specifically, we base our approach on the competitive rational storage model, which is the staple of the literature on the prices of storable commodities (2). Storage is a key feature of these markets because it allows prices to respond differently to a short-lived shock than to a long-lived shock. If there is a one-year demand increase, then market participants can draw down inventory and mitigate the price impact; they can replenish inventories in later years. However, a permanent demand increase cannot be met by drawing down inventory.

Carter *et al.* (2017) show in their Figure 3 and equations (9)-(11) the three fundamental equations of the storage model:

(i) Inventory Supply, i.e., amount of grain this year's market is willing to put into storage as a function of price.
(ii) Inventory Demand, i.e., amount of stored grain next year's market is expected to demand as a function of price.
(iii) Supply of Storage, i.e., the price at which storage firms are willing to store as a function of inventory quantity.

The empirical model is a partially identified vector autoregression (VAR) that estimates these three fundamental equations. The model also includes a fourth variable (index of real economic activity) to account for the role of global commodity demand in driving price cycles (3).

We follow Carter *et al.* by specifying the VAR using the following three assumptions to account for the endogeneity of prices and inventory (1). First, shocks to the relevant commodity market (corn, soybeans, or wheat) do not affect real economic activity within the same year. Second, the marginal cost of grain storage does not depend on the commodity price. Third, the short-run (within-year) elasticity of demand for the commodity is as estimated in Adjemian and Smith (2012)(4). Carter *et al.* also show that replacing the third assumption with the following three assumptions has little effect on the estimates: (i) short-run elasticity of demand for current use exceeds −0.1 in absolute value; (ii) inventory-to-use ratio never exceeds 0.4, which is the sample maximum; and (iii) the elasticity of next year's net supply is not less than the elasticity of current net supply. These assumptions which we adopt as well are further described in Carter *et al.* in the subsection titled "VAR Model and Identification" (1).

In a VAR, any data that do not fit the equations exactly contain an error, or shock. These shocks represent shifts in the relevant curve. The RFS implies shocks to both inventory supply and demand; it constitutes both a reduction in inventory supply and an increase in inventory demand. If we were to set the inventory supply and demand shocks to zero, then we could solve the model for a counterfactual BAU price in the absence of any shocks to those equations.

Instead, we define the counterfactual BAU scenario to include shocks to production for each commodity. For soybeans, we also allow shocks to soybean imports by China, as explained below. We incorporate these shocks as described in equation (27) of Carter *et al.* (2017). To measure these shocks, we use the difference between actual production and imports and the World Agricultural Supply and Demand Estimates (WASDE) that are made in May of each year. The May WASDE report is the first one released in each crop year.

In the subsequent subsections, we describe our model input and background assumptions related to ethanol production volume and demand, crop production and demand, and our price model specification and estimation.

Price model input – Estimated ethanol volumes

Carter *et al.* (2017) estimate that the 2007 RFS increased mandated ethanol use by 5.5 billion gallons (20.8 GL) per year (1). This is further illustrated by Fig. S7, which shows mandated and actual ethanol production since 2000 beside projections made by the United States Department of Agriculture (USDA) in February 2006 and February 2007. The difference between the 2005 and 2007 RFS mandates began at 3.6 bgal (13.6 GL) of ethanol in 2008. It rose to 4.4 bgal (16.7 GL) in 2009 and averaged 5.4 bgal (20.4 GL) in 2010-12.

In February 2006 the USDA projected that ethanol production would be quite similar to the 2005 standard. As noted earlier, this level would meet the oxygenate standard for reformulated gasoline under the Clean Air Act. However, a boom in ethanol production capacity occurred in 2006. By the end of that year, enough ethanol production capacity was under construction to more than double production. To reflect this building boom and the forthcoming RFS expansion, the USDA's February 2007 projections jumped above its February 2006

projections. The anticipated increase in future corn demand from the ethanol industry incented corn producers to store corn to receive higher prices. For this reason, we measure the effect of the 2007 RFS on grain markets beginning in late 2006.

The RFS also requires increased biodiesel use. Our approach to estimating the price effects captures any effects of biodiesel on crop prices. However, we expect the effect of biodiesel on soybean prices to be small. Half of U.S. soybeans are exported whole. By weight, about 80% of each domestic bean becomes meal and the other 20% becomes oil. Of this oil, 30% was used to make biodiesel in 2017, or about 3% of the weight of U.S. soybeans. Another reason we expect the effect of biodiesel on soybean prices to be small is that soybeans produce oil and meal in fixed proportions. An increase in demand for oil means the market gets more meal than it wanted, which lowers the price of meal thereby mitigating the effect on soybean prices.

Price model input – Corn, soybean, and wheat production

The RFS affected corn, soybean and wheat markets in two major ways. First, the mandated increase in ethanol production created additional demand for corn, since nearly all ethanol used in the U.S. is produced from corn. Second, increased demand for corn causes farmers to plant more corn, leaving less land available for other crops. This in turn reduced the supply of soybeans and wheat, causing these prices to increase as well.

Production of corn, soybeans and wheat occupies about two-thirds of U.S. cropland. The top panel of Fig. S8 shows harvested area of these three crops since 1995. In the mid-90s, these crops occupied similar amounts of land. Since then, corn and soybean area has increased and wheat area has declined. Corn area increased by 24% in 2007 as the expansion in the RFS loomed. Much of this increase was in central Corn Belt states such as Iowa, Illinois and Indiana, where corn is typically rotated with soybeans (5). After some reversion towards the mean in 2008, corn area remained high and then trended upward. From 2001-05, average corn area was 1% below soybean area. From 2006-10, average corn area exceeded soybean area by 8%. Soybean area recovered in the later years in response to increased demand for exports to China.

The bottom panel of Fig. S8 shows a steady increase in corn production, aside from the 2012 drought, which affected corn production much more than soybeans or wheat. Soybeans produce fewer bushels per unit of area than corn and wheat, but soybean production increased steadily throughout the sample period; it increased from 2.2 billion bushels in 1995 to 4.4 billion bushels in 2017. Wheat has steadily become less prevalent. Between 1995 and 2017, wheat area declined by 38%. Wheat production declined 20% during the same period because increasing yields offset some of the area decline.

Figure S9 shows the rates of use for corn, soybeans, and wheat in the U.S. since 1995. For corn, the dominant change reflects the increase in ethanol use. About one-third of each corn kernel that enters an ethanol plant is recycled as dried distillers grains (DDG), which are used for animal feed and have a price similar to corn grain. The other two-thirds of the kernel — the starch — is converted to ethanol. Fig. S9 displays these two components separately; the black area in the figure (denoted "ethanol") is the net amount of corn used for ethanol. The amount of corn used directly for food is relatively constant over the period. With the exception of the 2012

drought year, the quantity exported is also relatively stable. Note that the food category for corn and wheat also includes seed and industrial uses, and that these two categories are included in the residual category for soybeans.

Soybean exports grew by a factor of 2.5 between 1995 and 2017. By 2017 half of all U.S. soybeans were exported. Much of this demand came from China, which consumed 22% of the world's soybeans in 2017, up from 8% in 1995. This increase in demand for soybeans created upward pressure on soybean prices in addition to the pressure from the RFS.

Almost all domestically consumed soybeans are crushed (processed into oil and meal) before use. Soybean meal is used predominantly as animal feed and oil is used for human consumption or to make biodiesel. By weight, about 80% of each bean becomes meal and the other 20% becomes oil. Since most of the soybean becomes meal and half of soybeans are exported, the proportion of U.S. soybeans by weight that end up as oil is small, as illustrated in Fig. S9. Soybean oil prices typically run about double meal prices, so while biodiesel would be more prominent by dollar value rather than by weight, it remains a relatively minor source of income for soybean producers.

Most wheat is either exported or used domestically for food, with a small amount employed as animal feed. The quantity used for food is relatively constant from year to year. This suggests that exports and animal feed demand are relatively more elastic and that it is these areas that adjust to accommodate fluctuations in production.

The price of corn increased by about 50% in fall 2006 and has remained at or above that level since (Fig. S10). In the first five years after the RFS signal hit the markets (Sept 2006 – Aug 2011), corn prices were up 77%, soybean prices up 62%, and wheat prices up 62% relative to the last five pre-RFS years (Sept 2001–Aug 2006).

To ascertain how much of these price increases can be attributed to the RFS, we estimated a model that controls for other factors including the business cycle, global commodity demand, and yield fluctuations. All prices spiked around the 2008 commodity boom for reasons related more to the business cycle and global commodity demand than to the RFS. Prices spiked again in 2010-12 as relatively poor yields, especially for corn, coincided with high demand for biofuels and for soybean exports to China. Prices came back from these peaks after the 2012 drought. In summary, the three largest trends in the markets for corn, soybeans, and wheat after 2006 were higher prices, increased corn use for ethanol, and increased soybean exports.

Price model development and estimation

We apply the method in Carter *et al.* (2017), which uses a partially identified structural vector autoregression model to estimate the effect of the RFS on corn prices (1). Here, we update the corn model with data through the 2016-17 crop year, and we also apply the model to soybeans and wheat.

Our model incorporates the fact that the RFS is a persistent rather than transitory shock to agricultural markets. This distinction is important because persistent shocks have larger price effects than transitory shocks. The market can respond to a transitory shock, such as poor growing season weather, by drawing down inventory. This action mitigates the price effect. A

persistent shock, such as an increase in current and expected future demand, cannot be mitigated by drawing down inventory. To identify these two types of shocks, the model uses data on inventory levels and on the term structure of futures prices.

Table S4 summarizes the data used to estimate the model. It includes global real economic activity, which has been shown to be an important driver of commodity prices (3). To represent global economic activity, we use the index developed by Kilian (2009) from dry-cargo shipping rates (1, 6). As Kilian emphasizes, “the proposed index is a direct measure of global economic activity which does not require exchange-rate weighting, which automatically aggregates real economic activity in all countries, and which already incorporates shifting country weights, changes in the composition of real output, and changes in the propensity to import industrial commodities for a given unit of real output” (pg. 1056) (6).

The timeline at the bottom of Table S4 shows when the variables are measured. We measure inventory (*I*) at the end of the crop year. The real economic activity index (*X*), the futures price (*F*), and the spot price (*S*) are all measured at the same point in the middle of the crop year, i.e., in March, which is after the previous crop has been harvested and before the new crop is planted. Winter wheat is the exception, as it is planted late in the fall of the previous year. The arrow indicates that the futures price is the contract for delivery in November or December, which is after the next harvest.

Following (1), we use the futures and spot prices to compute the convenience yield, which is essentially the spot price minus the futures price. In computing the convenience yield, we also adjust the spread for interest and warehousing costs as in equation (13) of (1). Convenience yield provides crucial information for identifying the differing effects of transitory and permanent shocks. For example, in response to poor growing season weather, the spot price increases and inventory decreases, but the futures price does not increase much because traders understand that supplies will be replenished by the new harvest before the futures contract delivers. In such cases, the convenience yield increases. In contrast, persistent shocks such as the RFS cannot be met by drawing down inventory, so spot and futures prices increase by similar amounts and the convenience yield does not increase. Observing both the spot and futures price allows them to be identified separately, whereas observing only one price for a commodity does not.

Unlike corn and soybeans, which are relatively homogeneous, there are several classes of wheat produced in the United States. They vary according to where they are grown, the growing season, hardness, and protein content. Hard red winter wheat (HRW) makes up 40-45% of production in a typical year. It is grown mostly in and around Kansas, and is planted in the fall for harvest in early summer. Hard red spring wheat (HRS) makes up about 25% of production in a typical year and is considered the highest quality class due to its high protein content. It is grown in the Northern Plains states, and is planted in the spring for harvest in late summer. Soft red winter wheat (SRW) provides about 20% of production and most of the rest is white wheat. Robust futures markets exist for HRW in Kansas City, HRS in Minneapolis, and SRW in Chicago. The SRW futures market has a long history and remains the most actively traded, even though it lags behind the other two in production. The HRW and HRS futures markets are newer and have reported viable prices only since the late 1970s. In our analysis, we use SRW prices until March 1976, after which we switch to HRW and HRS futures prices. The results are the same if we instead use a weighted average of the three prices after 1976.

Estimating the incremental effect of the RFS requires an estimate of ethanol use that would have occurred in the absence of the RFS. This business-as-usual amount depends on factors that are difficult to quantify, including the true value of ethanol to the fuel industry and the extent to which, by guaranteeing demand for ethanol, the RFS caused large capital investment in ethanol plants and fueling infrastructure. Thus, rather than estimate the BAU ethanol quantity directly, we estimate the difference between the BAU and observed quantities. For price estimates of each commodity, we follow (1) and fit the model using data prior to the 2006 crop year and use them to project business-as-usual (BAU) prices that would have occurred after 2006 in the absence of the RFS.

To assess the model fit, we compute the Corrected Akaike Information Criterion (AIC_C) and Bayesian Information Criterion (BIC), and we evaluate the impulse response functions for concordance with economic theory. We generate confidence intervals for the impulse response functions using a recursive-design wild bootstrap with 10,000 replications (7). For each bootstrap draw, we estimate the identified parameter set and the range of impulse responses defined by that set. We keep only draws that satisfy our identification conditions. This exercise produces 10,000 bootstrap draws for both the estimated lower and upper bounds of the identified set. For this component of the price impact analysis we set the lower limit of the confidence interval equal to the 0.05 quantile across draws of the estimated lower bound and the upper limit as the 0.95 quantile across draws of the estimated upper bound. This interval, as reported in Figs. S13 and S16, covers the identified set with probability 0.90, because 90 percent of the estimated parameter sets lie entirely inside it.

We estimate business-as-usual prices by simulating from the model what prices would have been if the markets had experienced the same shocks to (i) real economic activity, (ii) U.S. production, (iii) Chinese soybean imports, and (iv) the supply of grain storage that we experienced post-2006, but no other shocks. The average difference between observed prices and these simulated BAU prices provides an estimate of how much the RFS affected prices. Although Garcia *et al.* (2015) show significant decreases in convenience yield since 2006, especially for wheat (8), allowing observed post-2006 convenience yield shocks (supply of storage) to enter the BAU simulation reduces the estimated effect of the RFS on wheat prices by only two percentage points.

Estimating effects on crop rotations

Following estimation of the price impacts of the RFS, we subsequently assessed the response of crop rotations to changes in price. We assumed an estimated 30% persistent increase in the price of corn and 20% increase in the prices of soybeans and wheat (see supplementary results) and followed the approach of Hendricks *et al.* (2014) to estimate how changes in prices affect the likelihood of continuous corn, continuous other crops, and corn-other crop rotations (5, 9).

Crop rotation model input

To estimate our model, we built a spatiotemporal database of U.S. cropland fields, crop types, soil properties, climate data, and observed crop futures and basis prices. To delineate individual fields, we used field boundary data from the publicly available 2008 USDA Common

Land Unit (CLU) produced by the Farm Service Agency (10, 11). If CLUs were not available for a given area, then we used satellite-delineated field boundaries from Yan and Roy (12). Information on annual crop types, soil properties, and climate data for each field were then drawn from the Cropland Data Layer (13), the Soil Survey Geographic Database (SSURGO) (14), and the PRISM climate group (15), respectively.

Crop futures and local cash prices used for the model were obtained from the Bloomberg Terminal (16). In total, the dataset represents local prices from 1,367 corn markets, 1,252 soybean markets, 84 HRS wheat markets, 96 HRW wheat markets, and 123 SRW wheat markets that were continuously observed from 2004-16. National prices for cotton and rice were also included in areas where these crops are relevant alternatives to corn. While we do not observe georeferenced prices of rice or cotton, the production of these commodities is far more localized. The goal in collecting these prices was to construct estimates of the price that producers expect to receive at harvest time while they are making their planting decisions. Since corn planting does not take place before March, this expected price is constructed as the spread between the nearby and harvest futures prices plus the local price, averaged over the months of January and February. Depending on the commodity, this spread will be the difference between the price of a November or December contract and the price of a March contract. The spread between the nearby and harvest futures prices represents the market's expected cost of storing a single bushel from planting time to harvest time. Adding the local price to this spread completely compensates a producer that would store a bushel to sell at harvest time relative to selling at planting time.

Crop rotation model estimation

First, we estimated the impact of corn prices and other crop prices on the probability of planting corn or another crop on a given field. Our regression models are the same as those used in Pates and Hendricks (2021), which follow the frameworks of Hendricks *et al.* (2014) to account for the common practice of rotating crops (e.g., alternating between corn and soybeans)(5, 9, 17). The probability of planting corn if corn was previously planted on the field was estimated as

$$Prob\left(y_{it} = corn | y_{i,t-1} = corn\right) = \Lambda(\beta_{10} + \beta_1^C P_{it}^C + \beta_1^O P_{it}^O + \boldsymbol{\gamma}_1' \boldsymbol{X}_{it}),$$

and the probability of planting corn given that a different crop was previously planted was estimated as

$$Prob\left(y_{it} = corn | y_{i,t-1} = other\right) = \Lambda(\beta_{20} + \beta_2^C P_{it}^C + \beta_2^O P_{it}^O + \boldsymbol{\gamma}_2' \boldsymbol{X}_{it}).$$

The variable y_{it} is a binary indicator if the crop on field i in year t is corn or some other crop, P_{it}^C is the price of corn, P_{it}^O is the price index of other crops, and $\boldsymbol{X}_{it}$ is a vector of controls. To reflect local basis patterns, we derived field-specific prices using an ordinary kriging of observed prices from thousands of locations in the region. The controls in our model include the field's slope, National Commodity Crop Productivity Index (NCCPI), irrigation status, and binary indicators for extreme precipitation conditions during the planting season. We also include a linear time trend to account for technology change. We estimated logistic models as denoted by the function $\Lambda(\cdot)$. We estimated separate models in different Major Land Resource Areas (MLRAs) and soil texture groups to account for the fact that corn area may be more responsive to price in some regions.

We estimated the models for all fields greater than 6 ha (15 acres) that were in regions where (i) over 20% of the total area was cropland; (ii) more than 10% of cropland area was planted to corn; and (iii) more than 50% of the cropland not planted to corn was planted to a crop for which prices were available, specifically wheat, soybeans, rice, and cotton. This set of criteria ensured adequate data were available to train the model. Our final sample included 3.6 million fields that accounted for 91.6% of corn area between 2009-16, inclusive. A complete description of the modeling and data sources can be found in Pates and Hendricks (2021).

Next, we used the estimated model to simulate the impact of a change in prices on the probability of specific crop rotations. The probabilities of planting a corn-corn rotation ($Prob^{\{CC\}ROT}$), an other-other rotation ($Prob^{\{OO\}ROT}$), and a corn-other rotation ($Prob^{\{OC\}ROT}$) were calculated for a given price scenario as follows (Pates and Hendricks, 2021):

$$Prob^{\{CC\}ROT} = Prob^{corn} \times Prob\left(y_{it} = corn | y_{i,t-1} = corn\right),$$

$$Prob^{\{OO\}ROT} = (1 - Prob^{corn}) \times \left(1 - Prob\left(y_{it} = corn | y_{i,t-1} = other\right)\right),$$

$$Prob^{\{OC\}ROT} = \frac{1}{2}\Big[Prob^{corn} \times \left(1 - Prob\left(y_{it} = corn | y_{i,t-1} = corn\right)\right)$$

$$+ (1 - Prob^{corn}) \times Prob\left(y_{it} = corn | y_{i,t-1} = other\right)\Big],$$

where $Prob^{corn}$ is the long-run probability of planting corn calculated as

$$Prob^{corn} = \frac{Prob\left(y_{it} = corn | y_{i,t-1} = other\right)}{1 - Prob\left(y_{it} = corn | y_{i,t-1} = corn\right) + Prob\left(y_{it} = corn | y_{i,t-1} = other\right)}.$$

We calculated the change in probability of each rotation due to the RFS for each of the 3.6 million crop fields as the difference in the probability under the RFS scenario with observed prices and the counterfactual BAU scenario based on our estimates of crop price impacts from our vector autoregression model described in the previous section — 30% higher corn prices and 20% higher soybean and wheat prices. To estimate the change in area of specific crop rotations, we multiplied the change in rotational probability for each field by the corresponding field size. These field-level changes were subsequently aggregated to the county and national level for visualization and reporting, respectively.

Estimating effects on cropland area

We assessed the impact of the RFS on cropland area by estimating changes in the probability of cropland expansion and abandonment. To do this, we estimated the probability of transitions between cropland and both land in pasture or in the Conservation Reserve Program (CRP). These transition probabilities were estimated as a function of cropland, pasture, and CRP returns and trained using point-level data from the National Resources Inventory (NRI) from 2000-12. We then used the model to predict the change in transitions between 2008-16 based on changes in prices associated with the RFS (18).

Cropland transitions model input

We developed our model of cropland area changes based on point-level land use transition data from the USDA National Resources Inventory (NRI) collected by the Natural Resources Conservation Service (NRCS) (18). The NRI provides annual land use data at a sampling of points across the United States from 2000-12. For our analysis, we focused on cropland (cultivated and noncultivated) transitions with either pasture or CRP land. We also used information in the NRI about the land capability classification of each point and its soil texture. If a point was enrolled in the CRP, the NRI indicates the year of the general signup number associated with its enrollment. Because the point-level data from the NRI indicate the county in which a point is located — but not its GIS location — variables constructed from other data sources were then merged into the NRI by county.

We constructed cropland returns as a 10-year discounted stream of expected returns averaged across the relevant crops of the county, assuming a discount rate of 5%. Crops in the calculations include corn, soybeans, winter wheat, spring wheat, rice, cotton, and sorghum. Projected prices for the next 10 years were obtained from the Agricultural Baseline Database from the Economic Research Service (19). These prices are created as part of the USDA's longterm projections report. For expected crop yields, we estimated county-specific trend yields. Costs of production were from Economic Research Service Commodity Costs and Returns (20) and utilized at the Farm Resource Region level or groups of states — ERS has changed its reporting regions over time. We included costs for seed, fertilizer, pesticides, and custom operations, which represent the primary cost differences across commodities. Other cost categories available in the data were excluded because of their minor role and because their definitions have changed over time, which could have improperly distorted the model. For all categories, we assumed costs remain constant over the 10-year projection period for the stream of expected returns. Returns were then averaged across crops for each county, where the weight given to each crop was the five-year moving average of area planted to that crop. Pasture returns were calculated as an estimate of pasture rental rates, which were derived from information about animal stocking densities and the price of hay (21). Pasture stocking densities (measured in animal-unit months) at the county level were obtained from Atwood *et al*. (2005) who extracted the values from the STATSGO soils data and cleaned the data (22). Hay prices were a five-year moving average of prices from NASS (23). Translating animal-unit months into rental rates requires several other parameter assumptions that can vary across states. Instead of making such assumptions about these parameters, we calibrated our rent estimates by state so that our rent estimates were similar in magnitude to 2009-16 pasture rental rates reported by NASS.

Several important variables for the CRP were obtained at the county level through a Freedom of Information Act request. The return from enrollment in the CRP is the rental rate of newly enrolled contracts. While the CRP rental rate data available online report the average rent for all enrolled land, we used only the rental rate of newly enrolled contracts, which better represents the decision variable for farmers. We also utilized data on (i) the average Environmental Benefits Index of land offered — both accepted and rejected — for CRP enrollment; (ii) the area of land with expiring contracts in each year based on the original contract; and (iii) the area of land eligible for early contract release in 2015 (see (24)).

We used climate data at the county level (25), and assumed that farmers make land use decisions based on expected climate conditions and that these climate conditions are

approximated by a 30-year average of weather variables. Weather variables included were the water deficit, water surplus, growing degree days between 10°C and 30°C, and extreme degree days (days above 30°C). Water deficit and surplus were calculated from a daily water balance model. Water deficit represents the amount of reference evapotranspiration demand that cannot be met by available water. Water surplus represents precipitation in excess of evapotranspiration demand. See (25) for details.

In order to allow for geographic variation in the extensive margin response of land use to crop prices, we trained independent models for each of seven Land Resource Regions (LRR) that correspond to aggregated Major Land Resource Areas (MLRAs) from the Natural Resources Conservation Service. For our sample of NRI points to estimate the models, we selected Major Land Resource Regions (MLRAs) where (i) over 20% of total land area is crop production; (ii) over 10% of cropland is planted to corn, soybeans, or wheat; and (iii) more than 50% of total crop area was planted to crops included in our estimate of cropland returns. Fig. S11 shows the regions that met these criteria and were included in our analysis. The region label indicates the letter of the Land Resource Region (LRR). Multiple letters indicate that LRRs were combined. LRR *M* had many more NRI points than other LRRs and included some areas that were very densely cropped while other areas had a substantial portion of grassland. Therefore, we divided this LRR based on whether the Major Land Resource Region (subregions within the LRR) had grassland area less than or greater than 15% of the area of cropland.

Cropland transitions model development

We utilized the NRI data to estimate how changes in land use returns over time affect the probability of transitions between cropland and pasture and between cropland and the CRP (26–30). We estimated only these two transition types because there are very few transitions between cropland and other types of land use in our study region. Notably, between 2000-12 in our region, only 0.01% of cropland became rangeland, 0.02% was changed to forestland, 0.01% of rangeland transitioned to cropland, and 0.01% of forestland became cropland. These represent too small a sample of NRI data to estimate how returns impacted the likelihood of a transition.

The probability of expansion of cropland from pasture was estimated as

$$Prob\left(lu_{nt} = crop | lu_{n,t-1} = pas\right)$$

$$= \Phi(\theta_0^{crop} R_{mt}^{crop} + \theta_0^{pas} R_{mt}^{pas} + \varphi_0^{crop} \bar{R}_m^{crop} + \varphi_0^{pas} \bar{R}_m^{pas} + \boldsymbol{\delta}'_0 \boldsymbol{X}_n)$$

where $Prob\left(lu_{nt} = crop | lu_{n,t-1} = pas\right)$ denotes the probability that NRI point n has a land use of cropland in year t and pasture in year $t-1$ and this probability is a function of the returns to cropland (R_{mt}^{crop}) in county m, returns to pasture (R_{mt}^{pas}), and a vector of other characteristics of the NRI point ($\boldsymbol{X}_n$). The notation $\Phi(\cdot)$ denotes the cumulative normal distribution to indicate that the probability is estimated with a probit model. The probability of abandonment of cropland to pasture was estimated similarly as

$$Prob\left(lu_{nt} = pas | lu_{n,t-1} = crop\right)$$

$$= \Phi\left(\theta_1^{crop} R_{mt}^{crop} + \theta_1^{pas} R_{mt}^{pas} + \varphi_1^{crop} \bar{R}_m^{crop} + \varphi_1^{pas} \bar{R}_m^{pas} + \boldsymbol{\delta}'_1 \boldsymbol{X}_n\right).$$

The controls included in the regression to account for soil productivity include a set of binary variables to indicate if the land capability classification is 1 or 2 or whether the land capability classification is 3 or 4, as well as indicators for five soil texture classifications. Controls to account for the climate of each county included water deficit, water surplus, growing degree days, and extreme degree days. The models were estimated separately for each region in Fig. S5 because we expected that crop returns have a different impact on transitions in different regions.

A key difference between our specification and previous literature is that we controlled for average returns ($\bar{R}_m^j = \frac{1}{T}\sum_t R_{mt}^j$) to account for unobservable variables that may be correlated with returns. This specification is known as the correlated random effects probit model and assumes that, conditional on average returns and observables $\boldsymbol{X}_n$, any remaining unobserved heterogeneity is uncorrelated with returns (31). Intuitively, adding $\bar{R}_m^{crop}$ and $\bar{R}_m^{pas}$ as controls means that we are exploiting changes in returns over time rather than the pure cross-sectional variation in returns. The terms φ^{crop} and φ^{pas} are nuisance parameters to account for unobserved heterogeneity and should not be interpreted as causal parameters. The cross-sectional variation in returns is subject to concerns about omitted variable bias because the NRI points in counties with higher returns may be more likely to convert to cropland but for reasons not fully accounted for in our controls $\boldsymbol{X}_n$. The correlated random effects specification exploits changes in crop returns over time that occurred due to changes in the demand for crops. The correlated random effects model is similar to a fixed effects model but is free from bias from the incidental parameters problem (31). While the correlated random effects model substantially reduces endogeneity concerns, there could still be some remaining endogeneity. One potential source of endogeneity is that additional cropland area could decrease crop prices. This remaining endogeneity is expected to bias our estimates of cropland area response to price downward and understate the environmental impacts of the RFS.

The probability of expansion of cropland from the CRP (i.e., exiting the CRP) was estimated as

$$Prob\big(lu_{nt} = crop | lu_{n,t-1} = CRPexpiring\big)$$

$$= \Phi\big(\theta_0^{crop} R_{mt}^{crop} + \theta_0^{CRP} R_{mt}^{CRP} + \varphi_0^{crop} \bar{R}_m^{crop} + \varphi_0^{CRP} \bar{R}_m^{CRP} + \boldsymbol{\delta}_0' \boldsymbol{X}_n\big).$$

One important note about expansion of cropland from the CRP is that we estimated the model only for NRI points that were enrolled in the CRP the previous year and for which the contract may be expiring. Farmers enrolling in the CRP agree to a multiyear contract — typically 10 years. Therefore farmers make a decision about changing land use only when their CRP contract is expiring. While we cannot know the exact date an individual point expires, we can approximate this date because the NRI data indicate the CRP signup year for each NRI point. Complicating determination of the exact expiration year, however, is that the USDA offered two- to five-year contract extensions for contracts expiring between 2007-10 in order to stagger the expiration of CRP contracts (24). Using this information and the NRI data, we tabulated how often land exited the CRP for each signup year, determined the common exit years, and estimated the model only for points in the respective years of potential exits.

The probability of abandonment of cropland to the CRP (i.e., enrollment into the CRP) is estimated as

$$Prob(lu_{nt} = CRP | lu_{n,t-1} = crop, t = signup\ year)$$

$$= \Phi(\theta_1^{crop} R_{mt}^{crop} + \theta_1^{CRP} R_{mt}^{CRP} + \varphi_1^{crop} \bar{R}_m^{crop} + \varphi_1^{CRP} \bar{R}_m^{CRP} + \boldsymbol{\delta}_1' \boldsymbol{X}_n).$$

We estimated our model of CRP enrollment only in years where there was a signup for general CRP. There were signups for the CRP in 2000, 2003, 2004, 2006, 2010, and 2011. However, actual land use change usually occurs in the year after the signup, therefore we estimated the model of CRP enrollment in years 2001, 2004-07, and 2011-12. We include 2006 because there were two signups in 2006 and one signup was in the spring, and we observed a significant number of land use transitions to the CRP in 2006. Our models of CRP transitions are unique compared to previous literature because we account for the effect of the CRP contract on land use transitions.

The controls in the CRP transition equations were the same as for pasture, but they also included the average Environmental Benefits Index (EBI) of land offered for the CRP in the county. We did not use the EBI of the respective years due to endogeneity concerns — the EBI of land offered for the CRP increases when crop prices are high because less land is offered for enrollment. Instead, we used the average EBI of offered land over time as the control to account for the fact that CRP enrollment is more likely in some counties because of a higher EBI.

Cropland transitions model simulation.

For the simulations, we estimated the area of land that transitioned to and from cropland 2009-16, inclusive, for each region due to the RFS. For transitions with pasture, we first predicted the probability of transitions at each point with observed crop returns between 2009-12. The probability of transitioning was multiplied by the area of land the point represented — this is included in the NRI data — and aggregated to the region level. We then calculated new cropland returns if the price of corn had not experienced a 30% increase and the price of soybeans and wheat had not experienced a 20% increase, and we calculated the predicted area of transition to represent the counterfactual BAU scenario without the RFS. The average annual change in area of transition was then multiplied by eight to predict the total changes in transitions due to the RFS over the course of 2009-16, inclusive.

The same basic simulation approach was used to estimate the change in transitions with the CRP, except that we accounted for expiring CRP area and signups. To predict how much land exited the CRP we calculated the change in the probability of exiting the CRP if the contract was expiring and multiplied this by the total area expiring in a given year. For years 2013-16 that are outside the NRI sample period, we scaled our estimate of CRP exiting by the relative change in the number of CRP contracts with expiring area. The relative change in the number of expiring contracts was calculated from county-level data from the Farm Service Agency. To simulate CRP enrollment, we estimated how predicted enrollment changed in signup years between 2009-16. The only general CRP signups in this period were in 2010, 2011, and 2013. We assume that all points in cropland in 2012 were eligible for CRP enrollment in fiscal year 2013.

Estimating specific locations of change

After estimating the transition areas of cropland with pasture or CRP due to the RFS, we then used high resolution data on the likely locations of cropland transitions in order to spatially allocate and identify for further modeling the characteristics of converted land. To do this, we first mapped observed land use change at the field level during our study period, building upon the approach of Lark *et al.* (32) and using updated recommended practices (33) to extend the analysis up through the 2016 growing season (34). To enumerate environmental impacts, these data were then used to link the estimated extent of land use change associated with the RFS in each major LRR region to specific locations of observed conversion. Thus, while high-resolution data were used to identify the specific field-level parcels and characteristics of converted land, the data from the NRI were used to estimate the magnitude of this conversion that occurred within each region and how much could be attributed to the RFS. This hybrid approach thereby combined the NRI data's high certainty and long-term temporal coverage (prior to any RFS price signals — needed to estimate our probit model) with the field-level detail and specificity of the satellite-based land conversion observed during the study period (33).

Estimating water quality impacts

Determining the impacts of the RFS on water quality indicators due to changes in crop rotations and cropland transitions requires an assessment of the effects of various cropping systems and of recent cropland expansion and abandonment. To do this, we employed a process-based agroecosystem model — Agro-IBIS — to simulate fluxes of water, energy, carbon, nitrogen, and phosphorus across our study period for alternate cultivation scenarios based on the methods of Motew *et al*. (35) and Donner and Kucharik (36).

For both the crop rotation and cropland transition sets of scenarios, we simulated a common historical period, followed by unique simulations for each pathway. The common period was 1750 to 1960 when all state variables of the model (e.g., soil carbon and nitrogen) are "spun-up" to account for the legacy of historical land cover and agricultural practices. The datasets used to simulate this time period included historical land cover, nutrient applications, and irrigation extent. The second period was 1961 to 2016 and was simulated differently for the crop rotation and the cropland transitions impact pathways.

For crop rotations, we simulated five cropping systems uniformly across all agricultural land in the conterminous US (CONUS) under identical initial conditions: continuous corn (CC), continuous soy (SS), corn-soy rotation (CS), continuous wheat (WW), and corn-wheat rotation (CW). The spatial scale for the crop rotations modeling was 2.5 arc-minute grid cells and was performed for all land classified as cropland according to Lark *et al.* (37). To determine the impacts of the RFS, we multiplied the outputs for each cropping system by the change in its probability due to the RFS as determined via the econometric model described earlier. For all non-corn (i.e. "other") crops, including those not modeled, we estimated the water quality impacts as a weighted average of soybeans and wheat based on the planted area ratio of each crop within each county or region.

For cropland transitions, we modeled two land cover scenarios — cropland and noncropland — for the areas determined by the specific cropland expansion and abandonment locations described above. For this set of scenarios, we estimated impacts for each distinct patch of converted land that was classified as expanded or abandoned in the land transition model. We then compared the median patch-level losses of nitrogen, phosphorus, and sediment for 2007-16 between the cropland and non-cropland simulations to estimate the differential impact of cropland

area changes. These per-area differential impact values (or impact intensities) were then multiplied by the estimated areas of land use change due to the RFS within each major LRR region to estimate the total impact of the RFS due to increased cropland expansion and reduced abandonment.

Outputs from both pathway simulations included the median annual field-level losses of nitrogen (via potential nitrate leaching to groundwater or flux past a soil depth of 1.5 m) [kg of NO_3-N/ha], phosphorus (via runoff) [kg/ha], and sediment (via runoff) [tons/km^2] for the years 2007-16, which provided a recent 10-year simulation period that overlapped fully with our period of study (2008-16) as well as two Censuses of Agriculture (2007 and 2012), thereby providing broader representation of data inputs and conditions across the period of interest.

Below, we describe the development of the model inputs and datasets, including those for soil and topography, historic land-use/land-cover (LULC), nutrient application rates, and the extent of irrigation. In general, all inputs were resampled to 2.5 arc-minute resolution for the crop rotation simulations or maintained in their native resolution to determine patch-level characteristics for the cropland transitions simulations.

Agroecosystem model input — Soil texture and topography

We created maps of the major USDA soil texture classes based on the 30m resolution POLARIS dataset (38) which is a probabilistic remapping of the USDA Soil Survey Geographic database (SSURGO). We used values of percent sand, silt, and clay associated with the surface soil layer (0 to 5 cm depth) to predict the USDA textural class based on boundaries defined by the National Soil Survey Center (39).

We created maps of the following variables related to topography and that are needed as inputs to AgroIBIS: land surface elevation, slope, slope length and steepness factor (LS-factor), and slope length. All variables were derived from a 30m resolution, hydrologically conditioned land surface elevation dataset from the USGS Elevation Derivatives for National Applications (EDNA) project (40). We then calculated slope using the nine parameter, second order polynomial method from Zevenbergen and Thorne (41). Slope was then resampled at one arc-second using a simple nearest neighbor calculation.

The LS-factor used in the Modified Universal Soil Loss Equation (MUSLE), which is embedded in Agro-IBIS, was calculated following the method of Panagos *et al.* (42). First, we calculated flow accumulation and specific contributing area using the "Multiple Flow Direction" option in SAGA-GIS (43, 44). We then calculated the LS-factor within SAGA-GIS using the method from Desmet and Govers (45). Lastly, we resampled the LS-factor to one arc-second using a simple nearest neighbor calculation.

To calculate slope length, we first resampled land surface elevation to three arc-seconds using bilinear interpolation and then calculated slope length within SAGA-GIS using the method from Olaya (46). We then developed a method to modify the slope length based on the location of channels as defined by the USGS National Hydrography Dataset Plus (NHDPlus V21) (47). The original intention of the slope length term used in MUSLE (48) was to represent the length of slope before overland flow reaches a channel or some area with substantial deposition. Therefore, we set the value of slope length for grid cells that contain a defined channel to half of the cell-width. To implement this method, we converted the national seamless network flowline from NHDPlus V21 to a three arc-second raster with a value of 45 meters using ArcGIS. We then mosaicked this new raster dataset with the original slope length raster.

Agroecosystem model input — Historical land-use/land-cover

Land cover categories for the agroecosystem modeling were determined based on the vegetation types simulated in Agro-IBIS (Table S5). We used several gridded land cover datasets (Table S6) as well as historical county-level USDA Census of Agriculture data (49) to span this entire time period. Note that for post-1900 land cover and nutrient application rate map creation, we accounted for changing county boundaries over time by using county boundary shapefiles from the National Historical Geographic Information System (50).

We extracted data from each available year and each county of the USDA Census of Agriculture (hereafter referred to as the Ag Census) (49) over the period 1939-2012 including area associated with harvested cropland for each distinct crop type, pasture, irrigated cropland, and irrigated pasture. We then removed outliers and interpolated missing values from each county's time-series for each variable. Next, we grouped variables to create statistics relevant for the land cover map creation (Table S7). Note that the "wheat" category is comprised of wheat, oats, barley, buckwheat, emmer and spelt, rye, and triticale (all members of the Pooideae subfamily).

We used all input datasets from Table S6 to define open water grid cells based on whether they were ever classified as open water regardless of the year or dataset. We did this to avoid the case where open water cells (not simulated by Agro-IBIS) convert from or to land cells. We used a global dataset representing potential natural vegetation created by Ramankutty and Foley (51) to associate with years 1750-1900. This 151-year period with constant vegetation cover was used during part of the biogeochemical spin-up period of the model where rates are artificially accelerated so that a quasi-equilibrium is reached in a more rapid and computationally efficient manner similar to Motew *et al*. (35).

For the period 1901 to 2007, we used a combination of datasets (52–56) that specify land cover types that are natural (e.g., forest, grassland) or broad agricultural (e.g., cropland, hay/pasture), as well as historical county level Ag Census data (49) to allocate crop types and pasture within the broader agricultural land covers. We used a semirandom algorithm that accounts for the relative areas of cropland and crop type within a given county, similar to Hamlin *et al*. (57). Due to the county-level nature of the USDA Census of Agriculture data, we used historical county boundaries available from the National Historical Geographic Information System (50).

For the period 1901-98, we used data from the FORE-SCE model (55, 56) for the years 1938-98 combined with Ag Census data for the years 1939, 1944, 1949, 1954, 1959, 1964, 1969, 1974, 1978, 1982, 1987, 1992, and 1997. First, non-agricultural grid cells were determined based on the FORE-SCE model output and a look-up table. Additional modifications were needed for the "developed" and "mechanically disturbed forests" classes. If a cell was categorized as "developed," we used the developed subclass (high, medium, low intensity, open) from the 2011 USGS National Land Cover Dataset (NLCD) for that cell. Therefore, once a cell was "developed", its subclass did not change over the simulation. For the FORE-SCE model output in the "historical" (56) time period (1993-98), we converted the "mechanically disturbed forest" (i.e., clearcut) classes to the nearest forest subclass from the 2006 USGS NLCD.

Next, we developed a method to estimate agricultural land cover that included major crop types (corn, soy, wheat, alfalfa) at the subcounty scale. Broadly, this method uses the FORE-SCE model output to determine where within a county certain cover types should be located, combined with the Ag Census data to determine the relative proportions of each cover type within a county. In addition, this method addressed the challenge of mapping pasture area within the

FORE-SCE model and modified the dataset so that it was consistent with the pasture areas reported in the Ag Census. To do this, we isolated the grid cells categorized as "cropland" by the FORE-SCE model for each county. We then used the processed Ag Census dataset (see discussion above) to semi-randomly assign corn, soy, and wheat to grid cells based on each crop's area relative to the total cropland area as reported in the Ag Census. If no cropland area was reported in the census data but FORE-SCE simulated cropland for a given cell, then the "hay" class was assigned. Next, we isolated the grid cells categorized as 'hay/pasture land' by FORE-SCE for each county and used the Ag Census data to semi-randomly assign alfalfa, nonalfalfa hay, and pasture, based on each cover type's area relative to the total area of all three cover types as reported in the Ag Census. Following these land cover assignments, we calculated the total pasture area that had been assigned and compared it to the pasture area reported in the Ag Census. If the Ag Census pasture area value was greater than that which was currently assigned, then we randomly assigned a portion of grassland and shrubland within the county to pasture so that the areas matched. For our modeling purposes, we used the 1938 land cover for the years 1901-37.

Lastly, we used a nearly identical method for the period 1999-2007 using NLCD land cover data (52, 53) instead of the FORE-SCE model output. We used NLCD 2001 for years 1999-2003 and NLCD 2006 for years 2004-07. For the period 2008-17, we used the USDA-NASS Cropland Data Layer (CDL) and a look-up table to convert CDL land cover classes to vegetation types simulated by AgroIBIS. Look-up table values for crops not simulated by AgroIBIS were made based on the closest plant functional type with corn as the default in ambiguous cases.

Agroecosystem model input — Fertilizer and manure N and P application rates

Following completion of the land cover dataset, we used county-level estimates of nitrogen (N) and phosphorus (P) inputs to the land surface developed by the U.S. Geological Survey (58–63) for the period 1945-2012 for fertilizer and 1982-2012 for manure (Table S8). We determined crop-specific rates of fertilizer N and P application based on the total mass of fertilizer N and P applied at the county-scale and an assumption of constant ratios between fertilizer rates for corn and those for the other agricultural land covers (soy, wheat, alfalfa, nonalfalfa hay, and pasture). We assumed that total fertilizer mass for a given county (m_{fert}) could be calculated using the following equation:

$$(1) \qquad m_{fert} = F_{corn} \times A_{corn} + F_{soy} \times A_{soy} + F_{wheat} \times A_{wheat} + F_{alfalfa} \times A_{alfalfa} + F_{hay} \times A_{hay} + F_{pasture} \times A_{pasture}$$

where F_x is the county-average fertilizer application rate for a given crop x and A_x is the area devoted to crop x within that county. The constant ratios (Table S9) were determined based on current recommendations from several university extension publications (64, 65).

We used estimates of county-level fertilizer N and P mass from Alexander and Smith (58) for years 1938-85 (using 1945 values for the missing years of 1938-44), Gronberg and Spahr (60) for years 1986-2006 (using 1987 values for the missing year of 1986), and Brakebill and Gronberg (59) for years 2007-17 (using 2012 values for the missing years of 2013-17).

For manure N and P application rates, we used county-level estimates from Ruddy *et al*. (63) based on several Ag Census years (1982, 1987, 1992, and 1997) and applied them to the nearest year for the time period 1980-99. Similarly, we used manure data from Mueller and Gronberg (62) based on the 2002 Ag Census and applied it to the years 2000-04; and manure

data from Gronberg and Arnold (61) based on 2007 and 2012 Ag Census data applied to the years 2005-17.

For each year and county, the manure N and P application rates were determined by dividing the total manure mass by the total area devoted to cropland, hay, and pasture (as specified by the land cover data). Thus, manure application is assumed to be uniform across all cover types that could potentially receive manure in each county.

Agroecosystem model input — Irrigated extent

Maps of irrigated agriculture (cropland and pasture) for 1938-2017 were created based on the Moderate Resolution Imaging Spectroradiometer (MODIS) Irrigated Agriculture Dataset for the United States (MIrAD-US) (66) for the years 2002, 2007, and 2012, and processed historical data from the Ag Census (see above). First, we created a maximum irrigation extent map that included all grid cells that were identified as irrigated in 2002, 2007, or 2012. Next, for each county and year in the period 1938-99 we extracted processed Ag Census data from the nearest census year on the fraction of cropland and fraction of pasture that were irrigated. Using the fraction of cropland that was irrigated, we calculated the number of grid cells that would need to be classified as irrigated cropland based on the total cropland area from the land cover map for the current year and county. We then determined which grid cells were both within the maximum irrigation extent for a given county and classified as cropland for the current year. If the number of grid cells needing to be classified as irrigated cropland was greater than or equal to the number of overlapping cells (irrigation extent and those classified as cropland), then all overlapping cells were classified as irrigated. If the number was less than the number of overlapping cells, then cells were randomly drawn from the pool of overlapping cells so that the total area of irrigated cropland was satisfied. An identical method was then implemented for irrigated pasture. For the period 2000-17, we used the closest MIrAD-US dataset (e.g., the 2002 dataset for years 2000-04) to classify cells as irrigated if they were also classified as cropland or pasture.

Estimating greenhouse gas (GHG) emissions from land use change (LUC)

We used the nonlinear nitrogen effect model (NL-N-RR) of Gerber *et al.* (67) to estimate nitrous oxide (N_2O) emissions. For each change in crop rotation or cropland area due to the RFS, we used the associated change in N application data based on the agroecosystem model above and estimated the corresponding change in N_2O emissions. We converted our N_2O estimates to CO_2e by assuming a 100-year global warming potential of 265 (68).

We used the methods of Spawn *et al*. (2019) to estimate the ecosystem carbon emissions from RFS-related land conversion (69). Carbon emissions from soil and biomass degradation associated with land conversion were modeled for all observed cropland expansion, including methane (CH_4) emissions from conversion of organic wetlands. The model has been validated against field observations of post-conversion SOC change from Sanderman (70) and accurately predicts SOC emissions throughout the US for conversion subsequently managed with conventional tillage. Model predictions are also similar to those observed after conversion of CRP land when managed with conventional tillage (71).

In addition, a variant of the Spawn *et al.* model was created to assess forgone sequestration associated with reduced rates of cropland abandonment. This model was structurally the same as that used for conversion to cropland but used a carbon response function (72) for conversion of cropland to grassland to estimate expected soil organic carbon

accumulation over a 15 year period — the average length of a CRP contract. We thus assumed that any abandoned land would have been retired to the CRP and sequestered carbon for the duration of its contract. To attribute emissions to the RFS, we multiplied total emissions from all observed land conversion within a given LRR by the percentage of that region's observed conversion that could be attributed to the RFS, as estimated by the econometric model above.

Using the estimate of total cumulative emissions due to the RFS, we then calculated emissions per liter of increased annual ethanol demand. To do so, we first allocated total ecosystem carbon emissions over a 30-year period following the approach of the U.S. Environmental Protection Agency (EPA) (73). While most of the emissions associated with conversion to cropland are likely to be emitted near the start of the time period, this approach accounts for the uncertain timing and permanence of those emissions. As noted by (73), utilizing a longer annualization period would decrease the apparent emissions from the RFS, while a shorter period would increase apparent emissions. To this annualized value of ecosystem carbon emissions, we added the annual nitrous oxide emissions from crop rotation and cropland area changes. We then divided these combined annual emissions associated with the RFS by the increased annual demand in ethanol estimated from our price impacts model, and subsequently converted to emissions per unit of energy equivalent using a heating value of 21.46 MJ/L (73).

Integrating models

We estimated the overall effects of the RFS on environmental outcomes by summing the results of two independent pathways of influence—intensification and extensification—that integrated results from the price, land use, and biophysical models. The intensification pathway captured the impacts of the RFS as manifested through changes in crop rotations, whereas the extensification pathway captured those due to cropland area changes. A summary of model integration for each pathway is provided below, with details for each step and component available in the corresponding sections presented above.

Intensification – environmental effects of crop rotation changes

We estimated the RFS-induced effects of crop rotation changes on environmental outcomes by integrating outputs from the crop price, crop rotation, water quality, and nitrous oxide emissions models. For each individual crop field mapped and modeled in our study, we multiplied the probability of each rotation under the RFS and BAU scenarios by the area of the field and the per-area environmental impact. Total losses from nitrate leaching, soil erosion, phosphorus runoff, and nitrous oxide emissions on existing cropland for both the RFS and BAU scenarios were thus estimated as

$$L = \sum_i a_i \sum_m L_{m,i} \;\; \Pi_{m,i}$$

where a_i is the area of field i in hectares, $L_{m,i}$ denotes the 10-year median (2007-16) loss of nitrate, soil, phosphorus, or nitrous oxide per year for crop rotation m on field i, and $\Pi_{m,i}$ denotes the probability of planting a given crop rotation m on field i. The change in losses due specifically to the RFS was subsequently calculated as

$$\frac{\partial L}{\partial RFS} = \sum_i a_i \sum_m L_{m,i} \sum_j \frac{\partial \Pi_{m,i}}{\partial p^j} \frac{\partial p^j}{\partial RFS}$$

where p^j denotes the price of crop j and $\frac{\partial p^j}{\partial RFS}$ represents the change in crop price due to the RFS as estimated by our price impact model.

Extensification – environmental effects of cropland area changes

We estimated the RFS-induced effects of cropland area changes on environmental outcomes by integrating outputs from the crop price, cropland transitions, water quality, nitrous oxide emissions, and ecosystem carbon emissions models. We used the estimated change in crop prices due to the RFS and the marginal probabilities of transitioning between noncropland and cropland due the changes in crop prices to estimate the total area of transitions within each LRR. The area of transition within each LRR was subsequently allocated equally across all specific locations of observed land use change within each LRR, such that each field-level parcel of cropland expansion or abandonment was assigned a proportion of change that was due specifically to the RFS. This data fusion approach allowed us to utilize the long time period of the NRI data to estimate the response of land use to price while employing the high-resolution remote sensing data to determine the likely locations of these transitions that are important for understanding the environmental impacts.

To assess environmental impacts, we multiplied the proportion of change due to the RFS for each parcel by the parcel's area and its location-specific environmental effects. For the water quality indicators, we multiplied the RFS-attributed change by the per-area differential impact values (or impact intensities) for nitrate leaching, soil erosion, and phosphorus runoff. For GHG emissions, we multiplied the RFS-attributed change by the estimated nitrous oxide or ecosystem carbon emissions (mass per hectare) associated with each specific parcel of land.

Estimating uncertainty

We estimated uncertainty at multiple points of our causal analysis framework. Except for the price impacts, we propagated the uncertainty results throughout the connected components—from the land use models through to all subsequent environmental impacts. The methods for deriving each component of uncertainty are described below, with all results presented in the main text as 95% confidence intervals, reported as [lower limit (0.025 quantile), upper limit (0.975 quantile)].

First, to understand the range of plausible price responses to the RFS, we used the approach of Carter *et al.* (1) to independently estimate 95% confidence intervals for the changes in price from BAU for corn, soybeans, and wheat. In order to constrain the subsequent estimates and preserve computational tractability, we then specified the price impacts of the RFS to be equal to the median percent increase in corn, soybean, and wheat prices and used these results for simulation of the remaining models. Thus, while we estimate a range of plausible price impacts of the RFS, our environmental outcomes reflect only those due to the median estimated price effects.

Next, we estimated uncertainty in our crop rotation model using a clustered wild score bootstrap. This bootstrap works by multiplying the score function by a random variable, called a perturbation, with a zero mean and unit variance. Our econometric model is comprised of a set of

logit regressions, estimated via maximum likelihood. The expected value of the score function is zero at the solution to the maximum likelihood estimation procedure. This means that the product of the score function value and a mean-zero unit-variance perturbation has the same mean and variance as the score function itself. This allowed us to simulate random draws for our model parameters by drawing pseudo-score values. Because most of the variation in crop prices is temporal, we clustered our bootstrap by year to avoid underestimating the amount of uncertainty. In practice, we do this by drawing a random perturbation term for each year and applying it to every observation within the respective year and re-estimate our model parameters. We repeated this process 1000 times to produce our parameter distributions that are used to calculate 1000 different sets of crop rotation probabilities in the BAU and RFS scenarios. To ensure we have enough possible distinct sets of random draws, we used the perturbation distribution developed by Webb (74) which allows for six distinct perturbation values.

For our cropland transition model, uncertainty in the magnitude of cropland area change was estimated via a clustered bootstrap routine with 1000 replications. For each region of the model, the NRI points were resampled with replacement and the predicted change in cropland area due to the RFS was estimated for each replication. The standard error of the change in cropland area was estimated as the standard deviation across the bootstrap replications. Our estimate of uncertainty was robust to heteroskedasticity between points and autocorrelation for a given point since we clustered the bootstrap by NRI point. Previous analysis indicates minimal bias to the standard errors through spatial correlation because the NRI points are sufficiently sparse (26).

We then combined the bootstrapped replicate estimates from both the crop rotation exercise and that for cropland area with the biophysical model outputs to quantify uncertainty in terms of environmental indicators. For the crop rotation-based (i.e. intensification) estimates, we multiplied each of the replicate probability bootstraps for a given field by the area of that field and the impact intensity of each environmental variable. For each bootstrap, we summed the effects across all CONUS fields to generate a distribution (n = 1000) of total nationwide impact estimates for each environmental indicator from which we summarized the mean and 95% confidence intervals.

For cropland transition estimates (i.e. extensification), since bootstrapped estimates represented the RFS-induced change in cropland area within larger LRR regions, we used a variant of the crop rotation procedure that utilized observed patterns of land use change (37) to further account for the more nuanced geographies of change that underly these aggregated estimates. Because each bootstrap represented an estimated area of change within each LRR region, for each estimate we then randomly selected patches of observed land use change from Lark *et al.* (37) within the corresponding region until the sum of selected patch area was equal to that of the bootstrap's value. We then also summed the modeled environmental impacts associated with each of the selected patches. Repeating this procedure for each of the 1000 bootstraps of each LRR region provided a distribution of the estimated total environmental impacts within each LRR region and, when summed across regions, the nation.

Our approach did not capture uncertainty stemming directly from the biophysical and emissions models nor their inputs, as neither group of models natively quantify this uncertainty. To help address this limitation, however, we estimate the range of environmental outcomes due to uncertainty in both the magnitude and location of the underlying land use changes—thereby providing an indication of the corresponding variability in environmental outcomes. Nevertheless, the uncertainty ranges we report for emissions and other biophysical outcomes are likely still conservative estimates of total system uncertainty. In summary, across the study, we quantify

uncertainty associated with the price impacts of the RFS, as well as that arising from the magnitude of crop rotation and cropland area changes, their spatial locations, and the associated variations in environmental impacts.

Supplementary Text (SI Results and Discussion)

Supplementary results for price impacts

We estimated the effects of the RFS on corn, soybean, and wheat prices by comparing observed prices in the 2006-10 crop years to the BAU projections for those years. Table S1 shows that corn prices exceeded the BAU by 31%, soybean prices by 19%, and wheat prices by 20% in 2006-10. These estimates include 95% confidence intervals of [5%, 70%] for corn, [-8%, 72%] for soybeans, and [2%, 60%] for wheat. Thus, there is a wide range of plausible price effects in the model, but the point estimates round to 30% for corn and 20% for soybeans and wheat.

The observed and BAU prices for all 2006-16 crop years are presented in Fig. 1 of the main text, with the detailed outputs for our soybean and wheat models included in Tables S10-S13 and Figs. S12-S17. For the corresponding output for the corn model, see Tables 2-3 and Figures 5-7 of Carter *et al.* (1). As the criteria do for the corn model, the AIC_C and BIC indicate that the soybean and wheat models fit best when using a single lag, and the impulse response functions conform to predictions of economic theory.

The models contain two distinct identifying assumptions: one provides point identification while the other provides set (partial) identification. Because the assumptions differ, there is no reason that the point identified parameter should lie within the identified set. In all cases, the estimated price effects are very similar whether we use the point estimate or the identified set. The vertical bars of Fig. 1 are 95% confidence intervals that capture uncertainty in the identified set for the model parameters. The parameters are estimated using data from the 1961-2005 crop years and so are subject to sampling error.

As additional validation, we compared the detrended prices of corn, soybeans, and wheat that we use in our analysis with the predicted prices from the VAR model as written in equation (23) of Carter *et al.* (2017)(Fig. S18). These are predictions of the current-year price given current-year values of the other variables and prior-year values of all the variables. The R^2 values for log future prices in the price models depicted in Fig. S18 are 0.81, 0.81, and 0.57 for corn, soybeans, and wheat, respectively, for the period of 1962-2005 and 0.89, 0.86, and 0.89 for the period of 2006-2016. Thus, the model fits are somewhat higher in the post-RFS period than before. These predictions show how well our model estimates prices overall, and we believe that their congruence with observed prices helps demonstrate the validity of the model across time and crops.

Our main results show that corn prices jumped in 2006 and increased even more the next year as traders stored additional corn in preparation for the impending ethanol boom (Fig. 1). Note that in the figure and text, years refer to crop years, and thus a price jump in 2006 refers to the 2006-07 crop year and therefore to a price for the crop harvested in fall 2006, which we measure in March 2007. BAU prices also increased during this initial period due to strong global commodity demand. The relative effect of the RFS was lower than average in the 2008-09 crop years as the financial crisis and the corresponding crash in oil prices and gasoline demand caused a drop in demand for corn from ethanol producers. Then in 2010-11, along with worse-than-expected crop yields, increasing ethanol demand caused corn prices to rise again significantly above the BAU values. In these two years, we estimate that corn prices were more than 50% higher than they would have been without the RFS-induced shocks.

In the BAU scenario, the market would have reduced corn inventory in 2010-11, making the market more vulnerable to the 2012 drought than occurred in actuality. In the real world, the presence of persistent high ethanol demand prevented inventories from being depleted too much and thus made the market more resilient entering the 2012 crop year. As a result, the drought hit BAU corn prices harder than observed prices, and the observed 2012 spot price was 30% above the BAU price. Good weather through the remainder of our study period produced large crops, so corn prices have declined from their peak values, but these declines also occur in the BAU scenario.

The patterns for corn are mirrored in soybeans and wheat, especially in the 2006-10 period. The 2012 drought had much smaller effects on these commodities than on corn. There was a relatively small yield decline for soybeans and a yield increase for wheat in that year, so the BAU price does not spike for them as it does for corn. In the last few years, both the observed and BAU prices declined.

Our BAU projections become less credible as time passes. These projections assume only four sources of price shocks, and other potential sources of shocks became apparent after 2010. In particular, a severe drought in South America reduced soybean production from Argentina and Brazil in the 2011 crop year (i.e., the harvest that occurred in the early part of calendar year 2012). These two countries produce half of the world's soybeans. This event pushed soybean prices up significantly, but it is not accounted for in the BAU projections. As a result, the BAU soybean prices after 2011 may be too low.

Similarly, for wheat, the 2012 drought caused an increase in the demand for animal feed due to the reduction in available corn. As a result, wheat prices rose. This shock is not included in the BAU projection, so the BAU wheat prices after 2011 may be too low. Thus, if we were to estimate the effect of the RFS by averaging all years from 2006-16, we would obtain estimates for soybeans and wheat that are larger than 20% but that are likely biased upwards. Therefore we conservatively used the average for 2006-10, which best captures the effects of the RFS while reducing interference and uncertainty from events that occur much later.

For comparison, Carter, Rausser, and Smith (1) report an alternative estimate of the effect of the RFS on corn prices. They impose a permanent demand shift of 1.3 billion bushels on the model and find a new equilibrium with 31% higher prices (90% CI: 0.05, 0.95). Their 1.3 billion bushel corn demand increase is equivalent to 20.8 billion additional liters of ethanol, which matches our posited incremental effect of the RFS. Thus, the similarity of the estimate from (1) to ours in Table S1 further supports our BAU approach.

Supplementary results for crop rotations

The validity of the crop rotation model is assessed by comparing predicted values to observed values. We find the model predicts well. Figure S19 shows the predicted corn area compared to the observed corn area over time while figure S20 shows the same for each crop rotation. The predicted area of corn and each crop rotation changes similarly with the observed areas over time. On average, our prediction is within 1.8% of the observed area for corn. Figure S21 shows the performance of the model at predicting differences in the area of each crop

rotation across space. The model does a good job of predicting more area of a corn-corn rotation in regions with a larger observed corn-corn rotation area, and similarly for other crop rotations.

Table S14 reports crop rotation elasticities with respect to prices. Elasticities indicate the percent change in area for each rotation with respect to a 1 percent increase in price, holding all else constant. As expected, the model indicates that an increase in the price of corn increases the area of a corn-corn rotation and corn-other rotation and decreases the area of other-other rotation. We also find that an increase in the price of other crops decreases the area of rotations with corn and increases the area of an other-other rotation. Corn-corn and other-other rotations are elastic with respect to the price of corn, in part because the area of corn-corn and other-other rotations are smaller than a corn-other rotation.

Table S15 reports the aggregate corn acreage elasticity. We estimate a long-run own-price elasticity of 0.574 and a cross-price elasticity of -0.467. In other words, a 10% increase in the price of corn increases corn acreage by 5.74% across the nation, holding constant the price of other crops. The cross-price elasticity is similar in magnitude to the own-price elasticity, but of the opposite sign.

We estimate that the 2007 RFS increased annual corn extent by 2.8 Mha across our modeled region, which included about 91.6% of corn area in the U.S. Assuming a similar increase in the remaining areas of the country suggests there were approximately 3.0 Mha of additional corn each year following the RFS. According to the USDA, farmers planted an average of 37.0 Mha of corn annually between 2009-16. Therefore our estimate implies that farmers would have planted only 34.0 Mha of corn on average during this period had the RFS not occurred, which suggests that about 8.2% of the current corn extent is attributable to the policy. More broadly, the area planted to corn increased 5.1 Mha between the periods 1999-2006 and 2009-16. Thus, while several factors played a role in this expansion, we attribute roughly 60% of the recent increase to the 2007 expansion of the RFS. Absolute increases in corn area were largest in North Dakota, South Dakota, and northwestern Minnesota (Fig. S1, a-c). Relative to existing corn extent, the Mississippi Alluvial Plain and the Columbia Plateau region of Oregon and Washington experienced the greatest transformations, with more than a 100% increase due to the RFS (Fig. S1, d-f). In other words, roughly half of the current corn area in these locations can be attributed to the RFS.

This proliferation of corn occurred through changes in its rotation pattern relative to other crops. For example, the area of continuous corn rotations (i.e. corn planted as successive crops) increased by 2.1 Mha [95% CI: 1.8, 2.3] due to the RFS or 47% [36%, 63%] relative to BAU, with greatest influence in the Upper Midwest (Fig. S1). To accommodate this increase in corn monoculture, the area of non-corn (i.e. other) crops planted in back-to-back years decreased by 3.4 Mha [2.6, 4.2] or 10.8% [7.8%, 13.6%]. The area of corn planted in rotation with other crops varied by region throughout the US. In core agricultural locations, where rotation with other crops was already common (e.g., Iowa, Illinois, and Nebraska), there was a reduction in corn-other rotations associated with the shifting trend toward increased continuous corn production. On the other hand, in areas previously dominated by other crops, like soybeans and wheat (e.g., North Dakota, South Dakota, and the Mississippi Alluvial Plain), more corn was added to the landscape via rotation with other crops. In total across the study region, the area of corn-other rotations increased by 1.4 Mha [0.8, 1.9] or 4.6% [0.8%, 8.3%].

Supplementary results for water quality impacts from crop rotations

Continuous corn cropping systems generate on average 163% more nitrate leaching than continuous soybean and 145% more than continuous wheat systems (Table S16). These larger losses of nitrate for continuous corn are driven primarily by the larger amount of nitrogen fertilizer inputs for corn compared to the other cropping systems (Table S17). Patterns of nitrate leaching across the US (Fig. S22) reveal higher values for all cropping systems within the Corn Belt and the Mississippi Alluvial Plain, where substantial mineralization of nitrogen occurs due to soils rich in organic matter, where historical applications of nitrogen fertilizer were high, and where additional high inputs are currently applied. Nitrate leaching is also high in heavily irrigated regions in Nebraska and the Mississippi Alluvial Plain where nitrogen is more easily mobilized via irrigation-induced drainage. Lastly, areas with coarser soils and more precipitation are also subject to heightened drainage and leaching of nitrate (e.g. northern part of the Mississippi Alluvial Plain).

Regarding soil erosion and phosphorus runoff, continuous corn also leads to the largest impacts compared to other cropping systems, with 56% and 71% more soil loss and 58% and 40% more phosphorus runoff compared to continuous soy and continuous wheat systems, respectively. This is mainly due to the relatively high erosion risk associated with continuous corn relative to the other systems, and partially due to high P input for continuous corn relative to the other systems.

Variation in soil erosion and sediment loss (Fig. S23) is primarily driven by slope and runoff, with finer-grained soils and higher precipitation also contributing to this susceptibility. Phosphorus is bound to soil, so steeper slopes, finer-grained soils, and higher precipitation also lead to higher phosphorus losses. In addition, phosphorus can be lost downstream in dissolved form and the amount is dependent on its concentration in soil at the ground surface. Overapplication of phosphorus can build up this surface soil concentration and lead to higher risk of dissolved and soil-bound phosphorus. Spatial patterns of phosphorus runoff (Fig. S24) thus show higher values in areas with steeper slopes (e.g., western Iowa) and historical legacies of heavy application of fertilizer and manure phosphorus.

We calculated the differential impact of continuous corn versus the four other cropping systems to visualize where the impacts of continuous corn may be both greatest and least substantial (Table S18 and Fig. S25-S27). Maps of this difference (continuous corn vs. other systems) reveal a variable pattern where the vast majority of areas show much higher differential impacts while very few areas show slightly less. For nitrate leaching, the largest differences (i.e., where continuous corn has greatest impact) occur in the regions with the highest nitrate leaching values (Corn Belt and Mississippi Alluvial Plain), which shows that the impacts of changes in crop type are highest in the areas of most intense production. These high differential impacts are primarily driven by more nitrogen inputs for continuous corn relative to the other cropping systems (Table S17 and Fig. S28). The only area to show a slightly lower impact from continuous corn is in the south-central Mississippi Alluvial Plain, with wheat. Here the difference in primary production between corn and wheat is the largest (corn higher than wheat) and this leads to both relatively large differences in nitrogen uptake (more uptake by corn) and slightly more water use (less drainage in the corn system). Thus nitrate leaching is slightly less for continuous corn compared to continuous wheat even though the nitrogen inputs for wheat are less than 50% of that for corn.

The differential impact maps for phosphorus are also quite variable, however most areas show continuous corn to have greater impacts than the other cropping systems. This is primarily driven by two factors: inputs of P are higher for corn (Fig. S29), and corn is more susceptible to erosion compared to the other cropping systems. The highest differences occur in regions where the slope is relatively steep (e.g., southwestern Iowa). The only regions where continuous corn has slightly less phosphorus impact than other systems are in northwestern Iowa and the southern part of the Mississippi Alluvial Plain. In northwestern Iowa, high manure P inputs lead to high surface soil P concentrations for both corn and wheat but slightly less for corn because primary production is higher than wheat. In the southern Mississippi Alluvial Plain, higher primary production for corn leads to more mining of soil P and lower surface soil P concentrations relative to wheat. Low slopes in this region also minimize the differences in soil loss (and soil-bound P) between corn and wheat.

Supplementary results for cropland area

The validity of the cropland transition model was also assessed by comparing predicted versus observed land use. Figure S30 shows the predicted area of cropland compared to the observed area over time and figure S31 shows the same for the area of cropland transitions. The predicted area follows the same pattern as the observed area over time. The largest difference was in 2001 where the National Resources Inventory indicated a large transition from cropland to pasture that is not predicted by the model. On average, the predicted area of cropland is within 0.2% of the observed area. Figure S32 indicates that the model does well at predicting the Land Resource Region where cropland transitions occurred as well. The model predicts large transitions from CRP to cropland between 2007 and 2012 in Land Resource Regions where large transitions were observed.

Table S19 reports cropland transition elasticities. These elasticities indicate the percent change in transitions due to a 1 percent increase in the price of crops, pasture rent, or CRP rent. The elasticities are relatively large because they represent the percent change in transitions—not the percent change in final land use—and there are relatively few transitions. The price of crops has no significant impact on aggregate transitions between cropland and pasture. Elasticities of cropland transitions with CRP all have the expected sign and are statistically significant. When crop prices increase by 10%, the transitions from CRP to cropland increase by 73.5% and transitions from cropland to CRP decrease by 12.18%. An increase in CRP rental rates decreases exits from CRP to cropland and increases enrollment of cropland to CRP.

The five-year elasticities of aggregate cropland area with respect to prices are reported in table S20. Five-year elasticities are reported as a medium-run elasticity relevant to the time frame of our modeling. We estimate an elasticity of cropland with respect to crop prices of 0.071—a 10% increase in crop prices increases cropland area by 7.1%. This elasticity of 0.071 is likely larger than the national cropland elasticity implied by our model because we only consider the areas of the United States with substantial corn growing area and these areas are likely more responsive to crop prices than areas outside our analysis. Our estimate is similar in magnitude to other estimates from the literature. Previous estimates include the following: 0.07 (Li, Miao, and Khanna, 2019 (75)); 0.16-0.20 (Claassen, Langpap, and Wu, 2016 (30)); 0.05 for a 5-year elasticity (Ahmed, Hertel, and Lubowski (2009)(76) using the model estimates of Lubowski, Plantinga, and Stavins (2008)(26)); and 0.03 (Barr, et al., 2011)(77). Langpap and Wu (2011)

estimate an elasticity with respect to corn price of 0.059 in the Corn Belt and 0.142 in the Lakes States (29). The estimate of Langpap and Wu (2011) would be even larger if it included an increase in all crop prices as we estimate. Our estimate of the elasticity of cropland with respect to pasture rent is the opposite sign that was expected, perhaps because we do not have as accurate a measurement of pasture rents. The effect of CRP rental rates on cropland area is negative, as expected, and more inelastic than the effect of crop prices.

Overall, across transitions between cropland and noncropland (including pasture and CRP), we found that cropland expansion increased by 1.8 Mha [95% CI: 1.5, 2.1] and abandonment decreased by 0.4 Mha [0.1, 0.6] due to the RFS (Table S21). Combined, this resulted in a net increase of 2.1 Mha of cropland area that can be attributed to the RFS for years 2009-16. Note that for the model simulation and all related results, we predicted changes for eight conversion years, with the first transitions occurring between the 2008-09 growing season and the final transitions occurring between 2015-16. This approach may thus underestimate the total extensive margin land response to the RFS, as some land likely came into initial production prior to the 2009 growing season and after the 2016 growing season. Each of these aggregate changes in cropland area due to the RFS were significant at the 5% level. The largest increase due to the RFS was in the region *Mgrass* where expansion grew by 0.76 Mha and abandonment decreased by 0.26 for an overall increase of 1.0 Mha. Region *F* also saw an increase of 0.63 Mha and region *H* had an increase of 0.38 Mha due to the RFS.

Specific examinations of the subset of transitions between cropland and pasture revealed no statistically significant evidence that the increase in cropland returns due to the RFS increased conversion of pasture to cropland (Table S22). However, in region *Mgrass* we estimated an increase in conversions of about 0.12 Mha, which is about an 18% increase in the average number of conversions. Some of the estimates of cropland expansion show an unexpected negative sign, but only one is significant at the 10% level.

Instead, we found stronger evidence that the increase in cropland returns decreased the amount of cropland that transitioned to pasture. In region *Mgrass*, we estimated that about 0.21 Mha that were not abandoned would otherwise have been in the absence of the RFS. This effect is statistically significant at the 5% level. We also found significant evidence of reduced abandonment in the *KL* region. Our net estimate is that cropland area increased by only 0.06 Mha through transitions with pasture due to the RFS. However, the impacts differed by region and there was an 0.33 Mha increase in cropland in the *Mgrass* region due to transitions with pasture that is significant at the 5% level.

In contrast to the transitions with pasture, we found large and statistically significant impacts of the RFS on cropland conversions specifically with the CRP (Table S23). The largest increases in cropland expansion from the CRP occurred in regions *F* and *Mgrass* where they increased by over 0.63 Mha in each region due to the RFS. Region *H* also saw an increase in conversions of 0.32 Mha.

The increase in crop prices not only increased cropland expansion from CRP but also decreased the area of associated cropland abandonment (i.e., enrollment into CRP). Enrollment of cropland into CRP decreased by about 0.05 Mha in regions *F* and *Mgrass* and about 0.10 Mha in region *H*. Overall, net cropland area increased by 2.1 Mha due to the RFS from changes in transitions between cropland and the CRP.

We can compare our aggregated results to national-level data from the NRI to estimate the relative contribution of the RFS to all land use changes observed over the study period to put each change in perspective. Of note, cropland area had been trending downward from 1982-2007. Had the most recent trend from 1992-2007 continued, cropland area would have been 7.8 Mha lower than it actually was in 2015. Instead, cropland area increased nationally by 3.0 Mha from 2007-15, in part due to the RFS, but also due to several other factors. One estimate of cropland area without the RFS is shown as the point in Fig. S33 and is calculated as the 2015 NRI cropland area minus the impact of the RFS — simulated between 2008-15 for consistency with the NRI endpoints. This indicates that about 24% of the difference between trendline cropland area and actual 2015 area is due to the RFS, or that the increase in cropland area in 2015 was 32% greater than it would have been in the BAU.

Another way to assess the relative changes is to look at the contribution of the individual components of cropland area change, i.e., cropland expansion and reduced abandonment. From 2007-15, the NRI reports total cropland expansion of 8.7 Mha or an average of 1.1 Mha yr^{-1}. We estimate 1.8 Mha or 0.2 Mha yr^{-1} of expansion due to the RFS, which is 21% of the total observed by the NRI and 26% larger than what would have occurred without the RFS (Table S2). In a similar fashion, the NRI identified 5.6 Mha or 0.7 Mha yr^{-1} of abandonment 2007-15. We estimate this had been lessened by 0.4 Mha or 0.04 Mha yr^{-1} due to the RFS, which is 6.3% of the amount identified by the NRI or 5.9% less than would have occurred without the RFS.

<u>Supplementary results for water quality impacts of cropland area</u>

The water quality impact of recent cropland expansion and abandonment depends on the amount of land converted as well as the spatially variable impact intensity (impact per unit area) of converting from cropland to non-cropland and vice versa. We calculated county-level average impact intensities (loss per unit area) to visualize the county-specific differential impacts between cropland and non-cropland (Fig. S34). These impact intensities reveal substantial spatial variability for nitrogen, phosphorus, and sediment. Across all three variables, however, almost all counties had a higher impact associated with cropland than with non-cropland (shades of red in Fig. S34).

Areas with higher impact intensity for nitrate leaching tended to have coarser grained soils that are more susceptible to high drainage and leaching and have larger inputs of N fertilizer. High sediment yield impact intensities largely accompanied areas of steeper slopes (e.g., Appalachia and the Driftless Area of southwestern Wisconsin). Spatial patterns of phosphorus runoff intensities were similar to soil erosion due to the connection between erosion and sediment-bound phosphorus. However, higher phosphorus inputs to cropland (e.g. southeast US) also contributed to higher phosphorus impact intensities. The only region with slightly negative phosphorus impact intensities was the southern Mississippi Alluvial Plain where corn and soybeans are highly productive and able to uptake and reduce soil surface phosphorus concentrations more than non-cropland. Thus, the lower soil surface P and its low slope (very minimal erosion) led to lower P losses for cropland.

While the impact intensity results were primarily used to assess the parcel-level impacts attributed specifically to the RFS (see main text results), we also present here the total impacts – due to the RFS or otherwise – of all recent cropland expansion and abandonment to help understand the broader underlying trends and spatial patterns. Net impacts at the county level for all cropland expansion and abandonment were calculated for nitrate leaching, soil erosion, and

phosphorus runoff by accounting for the total impact (intensity x area) associated with cropland expansion and subtracting the total impact associated with abandonment. We then divided this total net impact per county by the total county land area to create a normalized net impact (mass per county unit area) for visualization. Nationwide net impacts for all cropland expansion and abandonment for nitrate leaching, phosphorus runoff, and soil erosion were 81.2 Gg, 0.931 Gg, and 903 Gg, respectively. Areas of high net impacts for nitrate leaching included the eastern Dakotas, northeastern Nebraska, southern Iowa, western Kentucky, and western North Carolina (Fig. S35). High net impacts for sediment and phosphorus yield occurred mostly in southern Iowa, western Kentucky, southwestern Wisconsin, and western North Carolina.

Supplementary results for greenhouse gas (GHG) emissions from land use change (LUC)

We found total ecosystem carbon emissions of 397.7 Tg CO_2e associated with the 2.1 Mha of additional cropland due to RFS. Following the approach of the EPA's regulatory impact analysis (RIA) (73), we amortize these emissions over a 30-year period, which equates to annualized emissions of 13.3 Tg CO_2e yr^{-1}. These emissions were induced by a modeled 20.8 billion liters per year increase in ethanol demand due to the RFS, which suggests emissions of approximately 637 g CO_2e per liter or a domestic ecosystem carbon LUC emissions factor of 29.7 g CO_2e MJ^{-1}.

In addition to these one-time emissions associated with land conversion, there are additional, ongoing emissions of nitrous oxide from the annual fertilizer applied to the additional cropland extent. We estimate these emissions at 1.3 Tg CO_2e yr^{-1}, which equates to an emissions intensity of 61.4 g CO_2e per liter of increased annual ethanol demand or 2.9 g CO_2e MJ^{-1} (Table S3). Including nitrous oxide emissions from crop rotation changes due to the RFS further raise land use nitrous oxide emissions to 4.1 Tg CO_2e yr^{-1}, 194.6 g CO_2e per liter, and 9.1 g CO_2e MJ^{-1}.

Several factors may cause these LUC GHG emissions estimates to be conservative, particularly for those associated with changes to cropland extent. First, recently expanded croplands are typically planted on lower quality land because the highest quality land is already in production (32, 78). Thus, the yields of corn planted on new croplands are lower, leading to lower yields of ethanol and higher emissions per volume of ethanol produced. New croplands planted to corn during the study period yielded, on average, 8% less than the national average (37), suggesting that the emissions per liter of ethanol produced from new croplands may be higher than that for average croplands reported here.

Second, we attribute 2.1 Mha of cropland area change 2008-16 to the 20.8 billion liter increase in annual ethanol demand from the RFS. However, it is likely that some land was converted to cropland due to the RFS prior to and following this period, thereby increasing the total area of LUC and emissions that should be attributed to the policy and associated ethanol demand.

Third, we quantify only the emissions from ecosystem carbon fluxes and onsite nitrous oxide due to fertilizer application. However, we also show substantial increases in nitrate leaching, phosphorus runoff, and sedimentation, each of which has been shown to increase GHG emissions from rivers, lakes, or other water bodies (79–81). Accounting of such downstream emissions would thus further increase emissions associated with RFS-induced LUC.

Conversely, our model of ecosystem carbon losses is notably agnostic towards management practices used after conversion and may therefore overestimate losses in some instances. Ecosystem C losses, particularly those sourced from soil organic matter, often play out over several decades. While the general trajectory tends to be that of C loss when natural ecosystems are converted to cropland and C gain from the opposite transition, there exist some ensuing management practices that can alter these trajectories to varying degrees by enhancing rates of C sequestration or slowing rates of C loss. Reduced-, conservation-, and no-tillage practices, for example, have been shown in some cases to minimize or even reverse soil C losses from some production systems (82). Non-conventional tillage regimes, however, are still not yet widely used in the United States, with only 37% of U.S. croplands adopting any type of reduced tillage in 2017 (83). Furthermore, rates of long-term no-till adoption remain significantly lower (84), and field studies suggest that even intermittent tillage can entirely undermine the C gains attained during intervening periods of no-till (85, 86). Lastly, the activity of converting grassland to crops frequently entails at least initial tillage to break up soil prior to subsequent cultivation. Because the largest relative C losses tend to occur in the year(s) immediately following conversion – before the effects of ensuing management might flatten the emissions curve – it is therefore likely that the act of conversion itself is more influential than ensuing management decisions in terms total C impacts of conversion. Thus, while our results collectively reflect the most common management and C outcome from land conversion, it is possible that emissions could be reduced or amplified based on subsequent management decisions.

Our GHG emissions analyses are designed to be comparable to those of the EPA Regulatory Impact Analysis (RIA) (73), yet our findings differ in both the magnitude of estimated LUC area as well as the net impact on emissions. For example, the EPA's RIA scenario for ethanol production uses the FASOM model to estimate that by 2022, there would be 0.36 Mha of increased cropland area, primarily coming from land classified previously as cropland-pasture. However, there are also simultaneous increases in forest pasture by 0.08 Mha acres and a decrease in forestland by 0.01 Mha. Though individual land use change contributions to emissions are not identified in the RIA, it is likely that the relatively small magnitude of predicted domestic cropland extensification along with forest increases attributed to the RFS are at least in part responsible for the unlikely net sequestration estimated for domestic LUC by the RIA.

Along with those changes to broad land use areas, the RIA estimates shifts in crop planting patterns and associated N_2O emissions. For example, the RIA estimated an increase of 1.5 Mha of corn and a decrease of 0.5 Mha in soybeans, as well as changes in other crop extents. FASOM was then used to sum all emissions associated with agricultural land (CO_2 and N_2O from cropland, pastureland, CRP land) and forestland (CO_2 from biomass, soil, and forest products) between the years 2000-22 for the control and their fuel-specific scenarios. Again, individual LUC contributions to emissions are not enumerated, but rather the difference between the control and baseline scenarios represents the change in total GHG emissions due to domestic LUC, and cumulative emissions are distributed across a 30-year time horizon after 2022 (with a 0% discount rate) to account for the variable timing of LUC GHG impacts. From all shifts in domestic land use – from both broad agricultural area and crop planting patterns – the RIA estimates emissions of -4.0 kg CO_2e $mmBtu^{-1}$ or -3.8 g CO_2e MJ^{-1} for corn grain ethanol (p. 362) (73).

Looking more broadly at the overall emissions identified in the RIA, it is worth noting that the primary estimate upon which the regulatory compliance of corn ethanol was determined reflects projected improvements in feedstock production and refining processing that were anticipated to occur by 2022. Similar estimates were also made for the GHG intensities of corn

ethanol production for the years 2012 and 2017. For example, the estimated carbon intensities (CI) for a base plant (corn ethanol dry mill with dry DDG and using natural gas for its process energy source) were already 33% and 10% higher than gasoline, respectively, and would rise to 79% and 56% higher after incorporating our results of domestic LUC (73). As such, the average CI of corn ethanol produced over the life of the RFS program from its inception to present day is likely higher than that projected for 2022. We focused on results for 2022, however, as these projections received the most vetting during the regulatory review process, formed the basis of the fuel compliance decisions, and most closely represent current conditions and other recent benchmarks.

Other models and assessments provide additional points of comparison for the LUC-associated GHG emissions of corn ethanol production. The California Air Resources Board, or CARB, implements the Low Carbon Fuel Standard (LCFS). In its original modeling in 2009, the LCFS estimated a LUC CI for U.S.-produced corn ethanol of 30 g CO_2e MJ^{-1}, which included emissions from both domestic and international LUC combined. In its updated modeling for 2015 and 2019, this LUC CI factor was reduced to 19.8 g CO_2e MJ^{-1}. This estimate is calculated using the GTAP-Bio-AEZ model, and its results are included in the California version of the Greenhouse Gases, Regulated Emissions, and Energy Use in Technologies (CA-GREET) (87). Using a representative simulation of the GTAP-Bio-AEZ model used in CA-GREET, we estimate 25.5% (5.0 CO_2e MJ^{-1}) of the total LUC emissions modeled by the LCFS occur domestically, with the remaining 14.8 CO_2e MJ^{-1} attributable to international land use change.

A more general version of GREET developed and distributed by the Argonne National Laboratory has been widely used by independent researchers due in part to its noteworthy ease of use (88–90). This version of GREET relies on LUC projections generated using the GTAP-BIO computable general equilibrium model parameterized with a host of *a priori* assumptions and user-selected emissions factors to predict LUC emissions associated with the demand for ethanol. Pertaining to domestic (U.S.) LUC associated with corn ethanol, GREET includes two LUC scenarios from which users can choose: (i) the “Corn Ethanol 2011” scenario which predicts 2.1 Mha of LUC with 55% LUC affecting “cropland-pasture”—a land use type equated to lands enrolled in the CRP—and (ii) the “Corn Ethanol 2013” scenario which predicts 1.9 Mha of LUC in total, 92% of which displaces “cropland-pasture”. Assumptions underlying the latter scenario which predicts less LUC have been called into question and shown to almost certainly underestimate LUC emissions (91, 92). Note, however, that the total extent of domestic LUC predicted by both of these GREET scenarios falls within the 95% confidence interval of our independent estimates of gross cropland expansion (1.5-2.1 Mha) and net cropland extensification (1.8-2.5 Mha). Thus, the primary difference between these estimates and ours stems from the estimated emissions associated with LUC.

Depending on the emissions factors applied to these LUC projections, GREET-based estimates of domestic LUC emissions can range from -2.3 g CO_2e MJ^{-1} using GREET’s “CENTURY/COLE” emissions factors with the Corn Ethanol 2013 scenario (the negative value, here, indicates net sequestration, rather than emission), to at least 9.5 g CO_2e MJ^{-1} when GREET’s “Woods Hole” emissions factors are used in conjunction with the LUC predictions of the Corn Ethanol 2011 scenario. Note however, that the “CENTURY/COLE” emissions factors responsible for the lowest estimates assume that cropland-pasture conversion—the most common form of predicted conversion—sequesters carbon, an assumption that is not supported by field observations nor independent modeling (92). Note also that the larger emission estimate generated using the Woods Hole emissions factors are definitively an underestimate since they inexplicably omit all emissions from cropland-pasture conversion. If the cropland-pasture

emission estimate generated using the GREET's "Winrock" emissions factors (5 g CO_2e MJ^{-1}) were used in place of the missing Woods Hole equivalent, estimated emissions would rise to 14.5 g CO_2e MJ^{-1} (84). GREET also provides separate estimates for international land use change ranging from 5-5.5 g CO_2e MJ^{-1} based on the Winrock emisisons factors (incomplete, smaller estimates are also provided based on the Woods Hole emissions factors without explanation).

While both GREET and CA-GREET domestic emissions estimates are lower than ours, we note that, with the exception of the questionable "CENTURY/COLE" emissions factors, the generalized GREET domestic emissions factors for each land cover type simply represent the national average carbon stocks of those lands as inferred from either literature review or sparse field inventories and are relatively agnostic to the geographies of land use change. By contrast, the approach we use integrates the latest high resolution data on vegetation and soil organic carbon stocks with highly resolved patterns of observed LUC to better reflect realized outcomes. Validation of our emissions estimates shows good agreement with independent field observations, particularly those from grasslands converted to conventionally tilled croplands (69).

It should also be noted that induced LUC, such as that modeled in our study and the others referenced here, is just one way of assigning a cost to the use of land. Others have found, for example, that if corn devoted to biofuels were replaced with the global average carbon cost of producing corn, the induced LUC emissions would be 200 g CO_2e MJ^{-1} (Supplementary Table 4 of reference (93)). Such references provide a helpful point of comparison, as any estimate of LUC less than this value suggests that either an equivalent amount of that crop will not be replaced or that it will be replaced at a fraction of the global average cost of production (93, 94). Estimates in both the RFS RIA and CARB LCFS, as examples, assume that at least a portion of the displaced crops is not replaced within the food supply (94).

Figs. S1-S35.

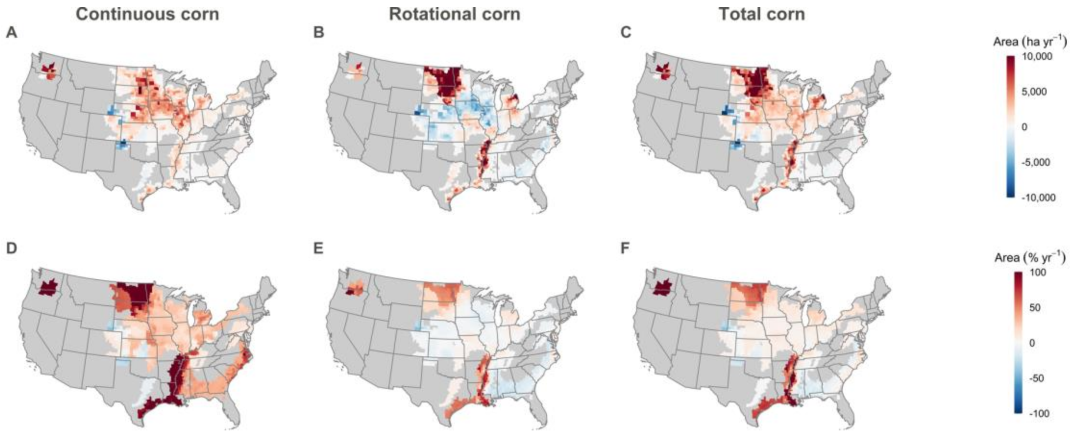

Fig. S1. Changes in crop rotations due to the RFS. (A-C) Absolute changes in crop rotation area within each county. **(D-F)** Relative changes in crop rotation area, represented as a percent of the rotation area in the BAU. Continuous corn represents cropland planted to corn in sequential years. Rotational corn represents cropland planted in rotation between corn and another crop. Total corn area is equivalent to continuous corn area + ½ rotational corn area.

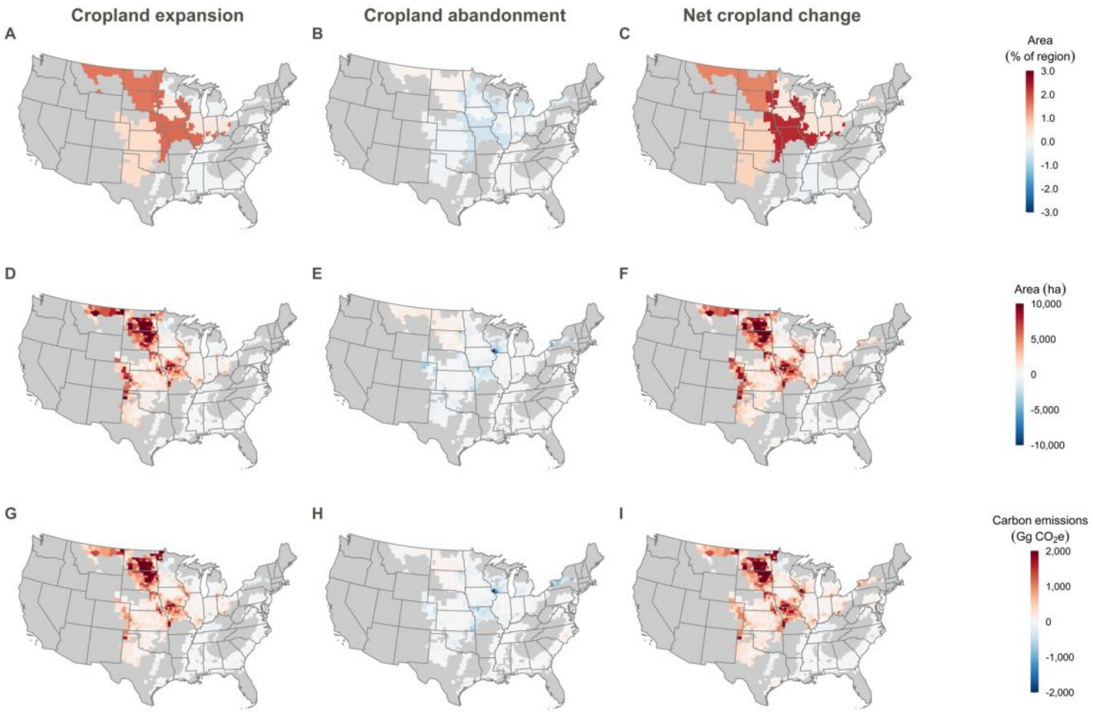

Fig. S2. Changes in cropland area and associated carbon emissions due the RFS. (A-C) Changes in cropland area as a percent of the total area within each aggregated MLRA region. **(D-F)** Absolute changes in cropland area within each county. **(G-I)** Changes in associated ecosystem carbon emissions.

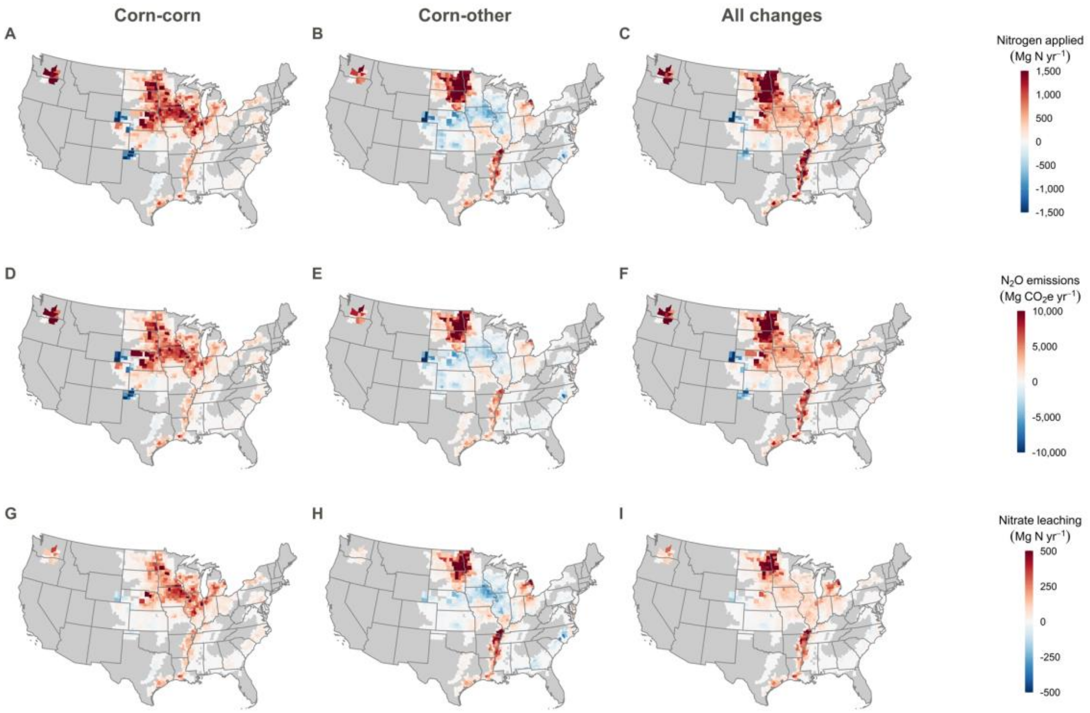

Fig. S3. Changes in nitrogen-related outcomes due to crop rotation changes under the RFS. (A-C) Changes in total applied nitrogen. **(D-F)** Changes in nitrous oxide (N_2O) emissions. **(G-I)** Changes in nitrate (NO_3^+) leaching.

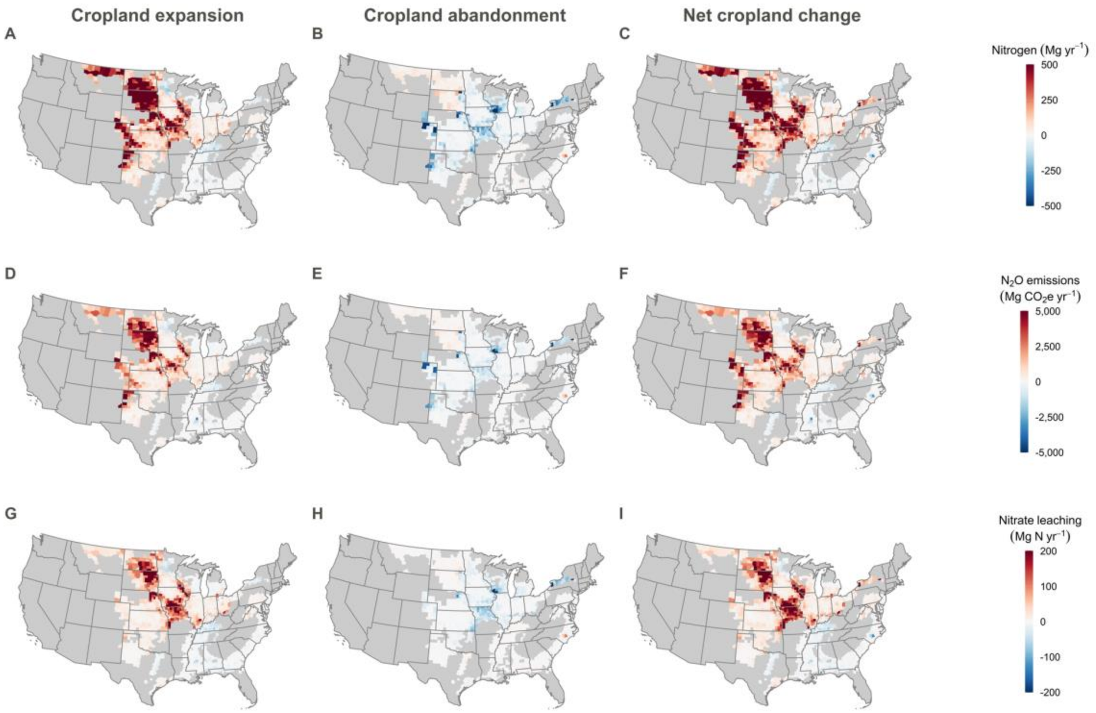

Fig. S4. Changes in nitrogen-related outcomes due to cropland area changes under the RFS. (A-C) Changes in total applied nitrogen. **(D-F)** Changes in nitrous oxide (N_2O) emissions. **(G-I)** Changes in nitrate (NO_3^+) leaching.

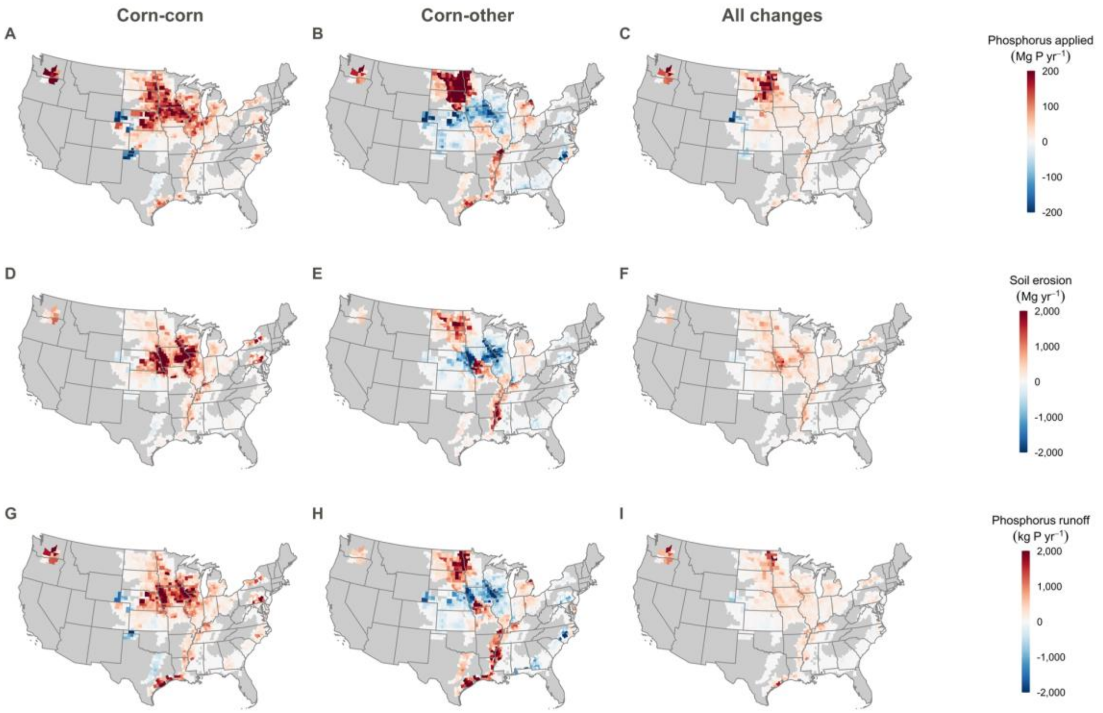

Fig. S5. Changes in phosphorus and erosion-related outcomes due to crop rotation changes under the RFS. (A-C) Changes in total applied phosphorus. **(D-F)** Changes in soil sediment loss. **(G-I)** Changes in total phosphorus runoff.

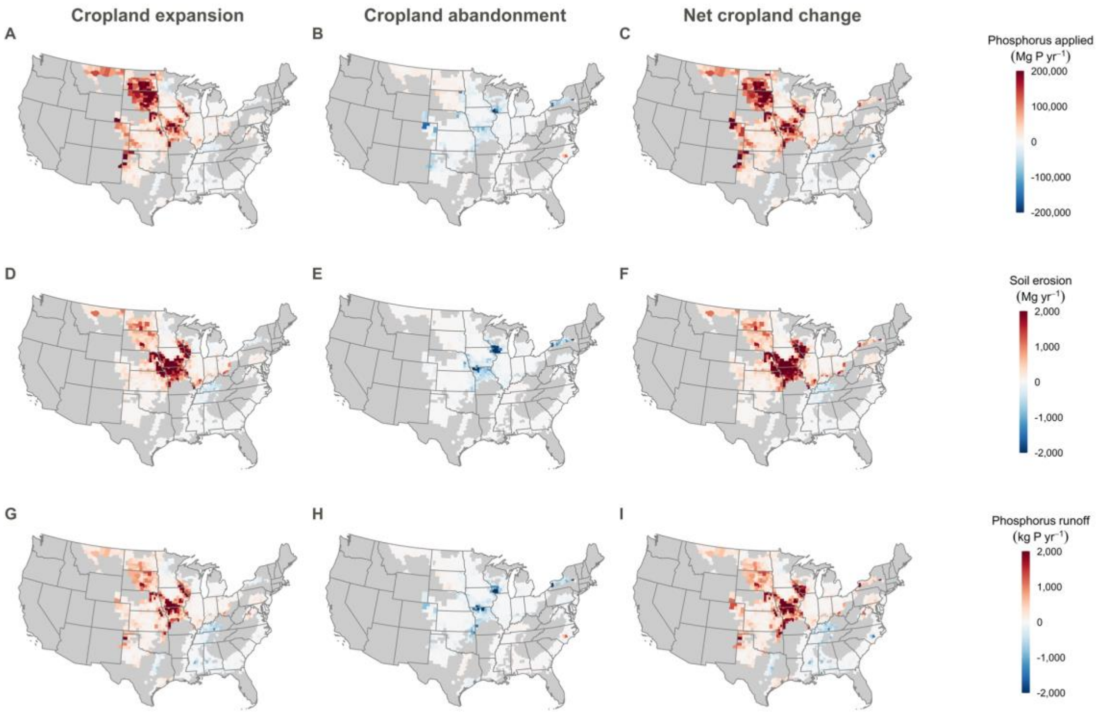

Fig. S6. Changes in phosphorus and erosion-related outcomes due to cropland area changes under the RFS. (A-C) Changes in total applied phosphorus. **(D-F)** Changes in soil sediment loss. **(G-I)** Changes in total phosphorus runoff.

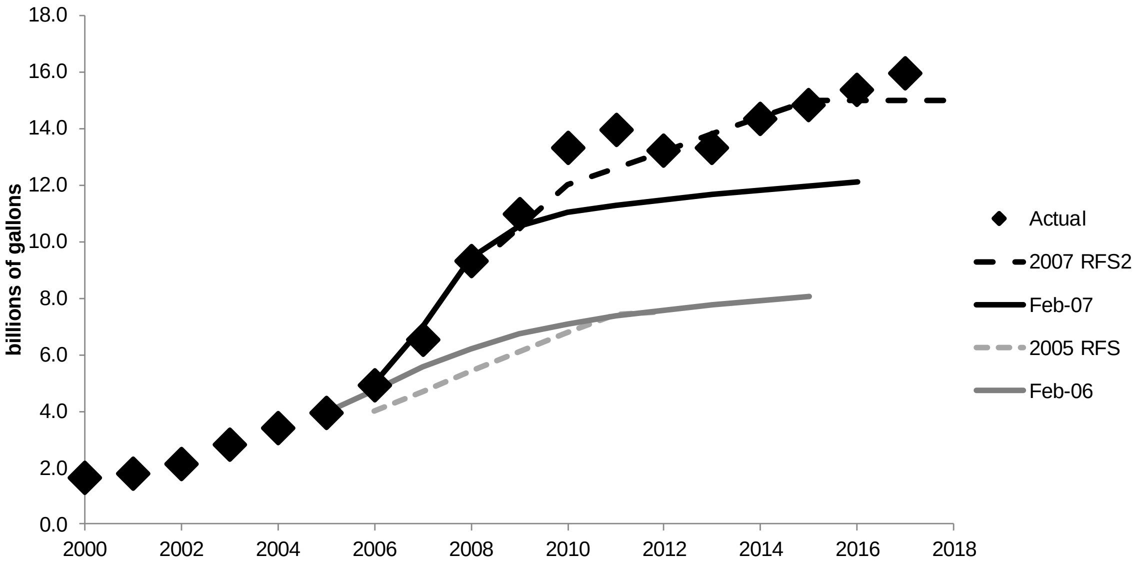

Fig. S7. Projected, mandated, and actual ethanol production. Dashed lines represented the amount of conventional renewable fuels mandated by the 2005 and 2007 versions of the RFS. Solid lines represent the amount of production projected by the USDA in February 2006 and February 2007.

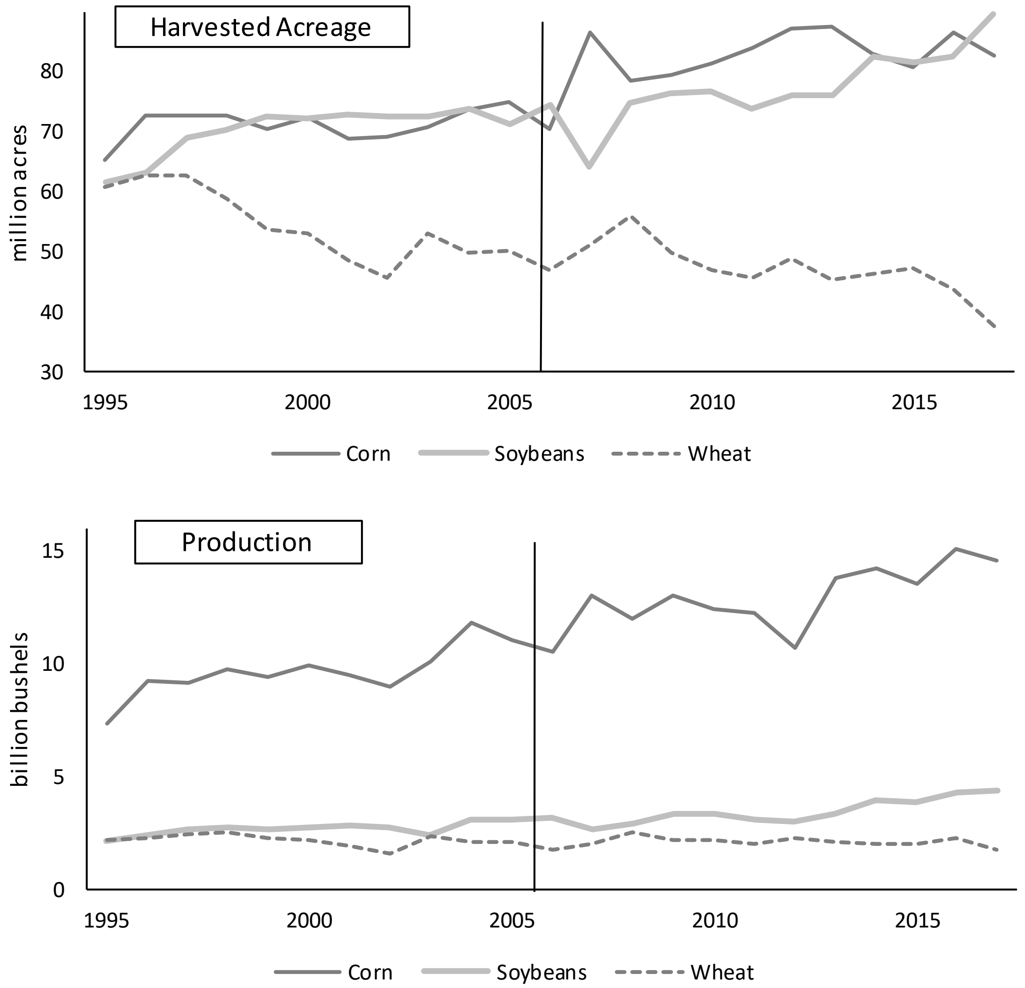

Fig. S8. Supply of corn, soybeans, and wheat. Vertical line at 2006 indicates when the 2007 RFS first affected grain markets. Data from USDA (23).

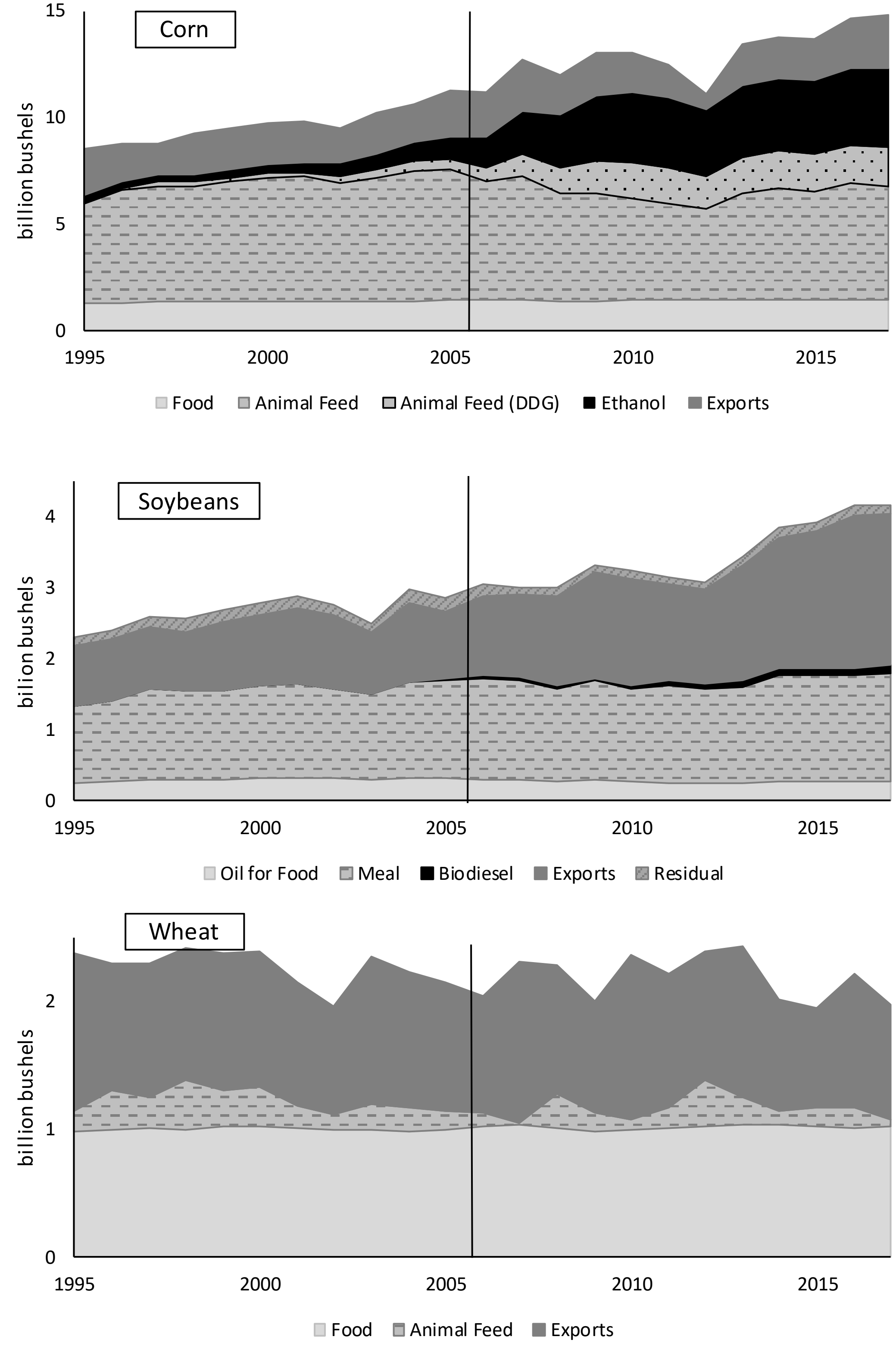

Fig. S9. Uses of corn, soybeans and wheat in the US. Vertical line at 2006 indicates when the 2007 RFS first affected grain markets. Data from USDA (23).

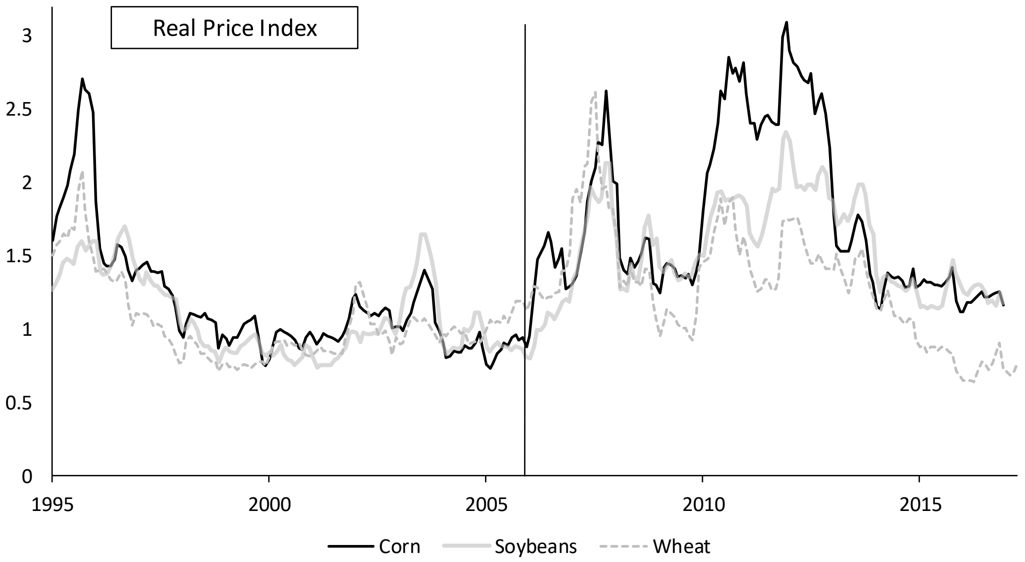

Fig. S10. Real price indexes for corn, soybeans and wheat in the U.S. Monthly prices deflated using the U.S. consumer price index for all items and indexed to average a value of one across the 2001-05 crop years. Corn and soybean prices are Central Illinois cash bids. Wheat prices are Kansas City hard red winter cash bids. Vertical line at 2006 indicates when 2007 RFS first affected grain markets. Time reflects the crop year, i.e., the label 1995 denotes September 1 of that year. Data from USDA (23).

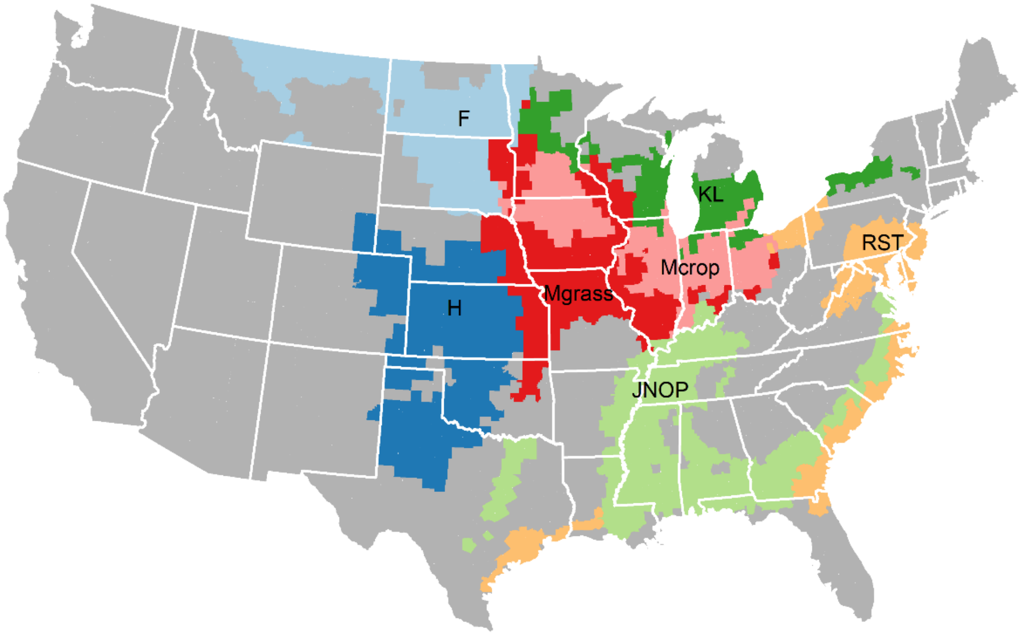

Fig. S11. Map of regions used in the econometric analysis of cropland transitions. Separate models were estimated for each region, with the region label indicating the letter of the Land Resource Region (LRR). Multiple letters indicate that LRRs were combined. LRR M had many more NRI points than other LRRs and included some areas that were very densely cropped and other areas that had substantial portions of grassland. Therefore, we divided this LRR based on whether the Major Land Resource Area (a subregion within an LRR) had grassland area less than (pink) or greater than (bright red) 15% of the area of cropland.

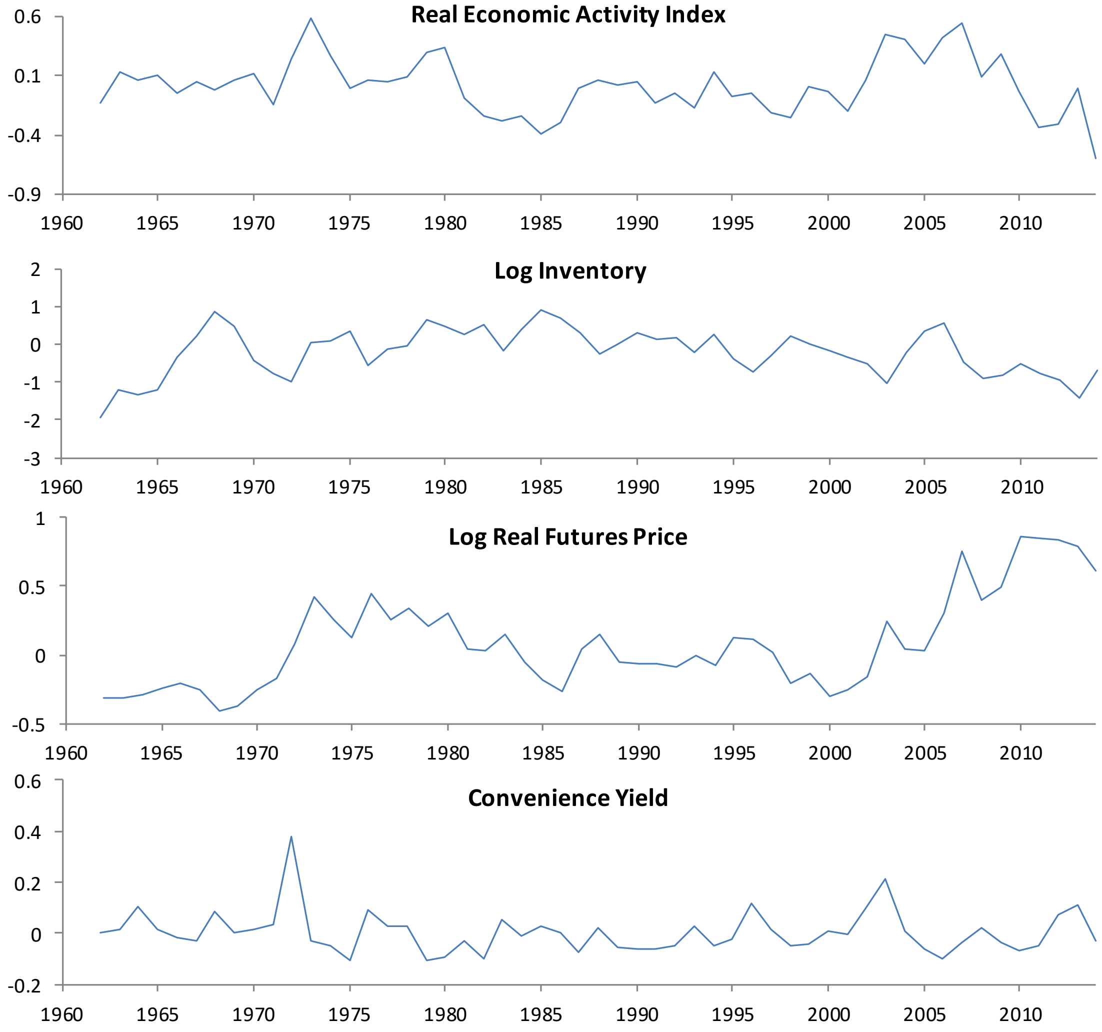

Fig. S12. Detrended data for key variables in soybean model. For clarity, this figure shows linearly detrended series, where we estimate the trend in the pre-RFS period (1961-2005). For the VAR estimation, we use the actual series and include a constant and linear trend in each equation of the model.

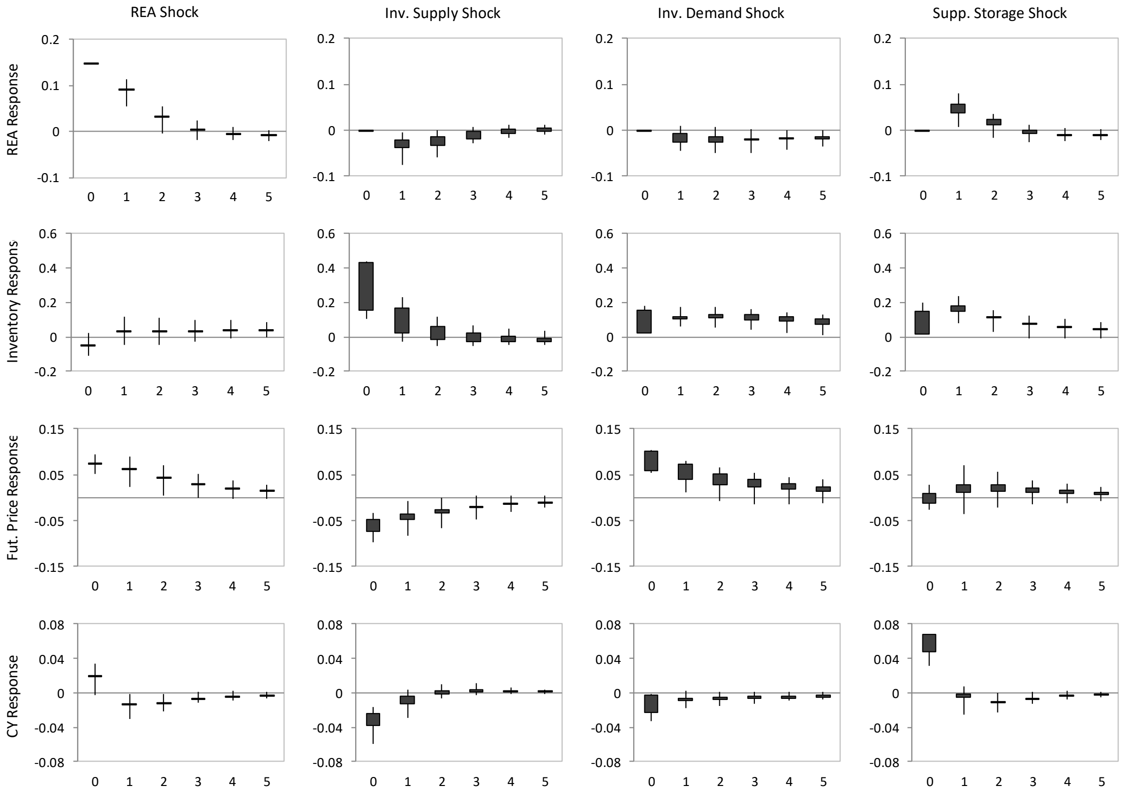

Fig. S13. Impulse response functions for soybeans. Responses to one-time one standard deviation shocks for the two-lag model. The dark boxes indicate the range of impulse responses in the identified set. The vertical bars indicate estimated confidence intervals that cover the true parameter with probability greater than 0.90. We obtain these intervals using a recursive-design wild bootstrap following the approach of Carter *et al.* (1).

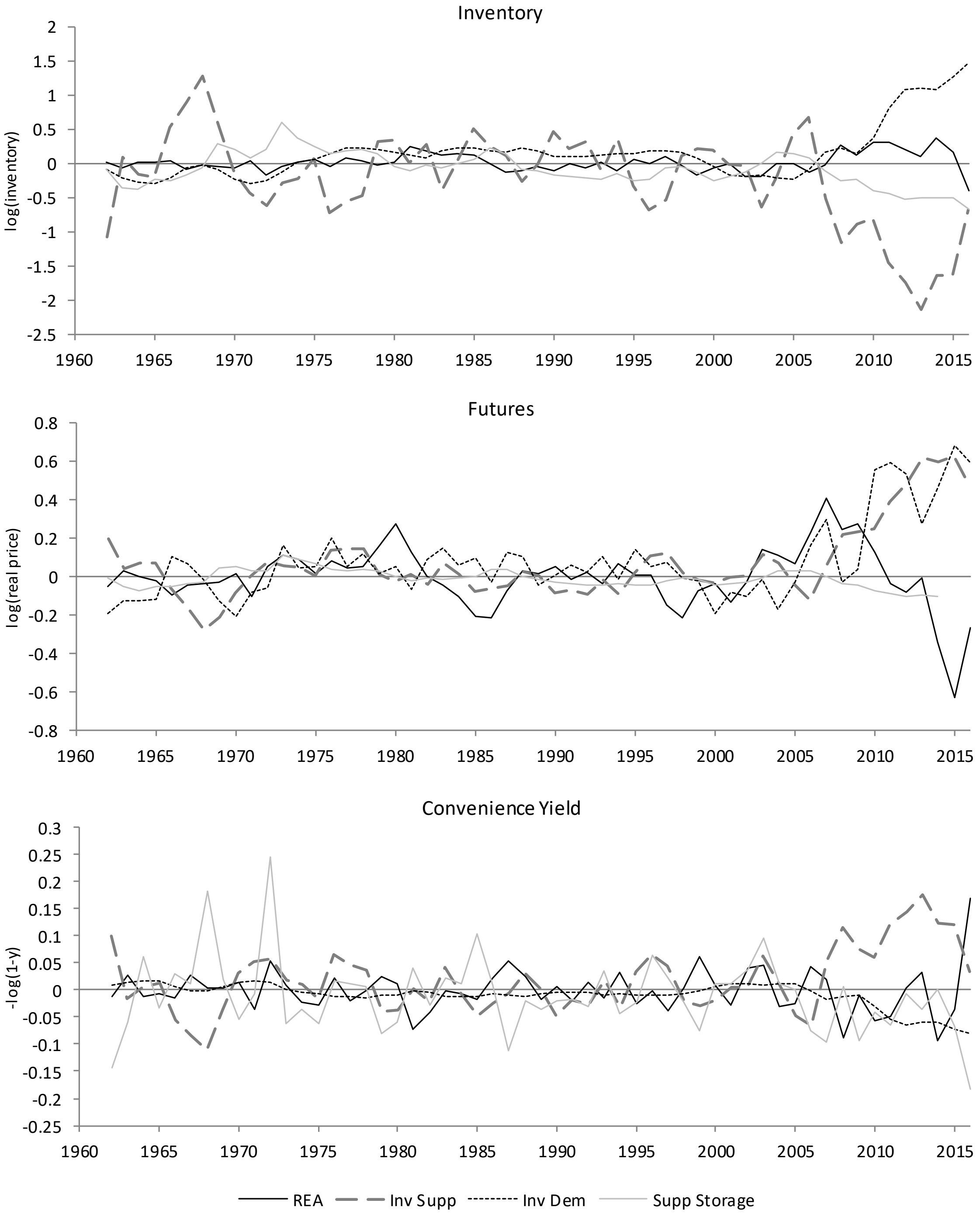

Fig. S14. Historical decomposition for soybeans. Figures show contributions of each shock to the relevant series for the one-lag model. The sum of the contributions equals the observed data (net of trend).

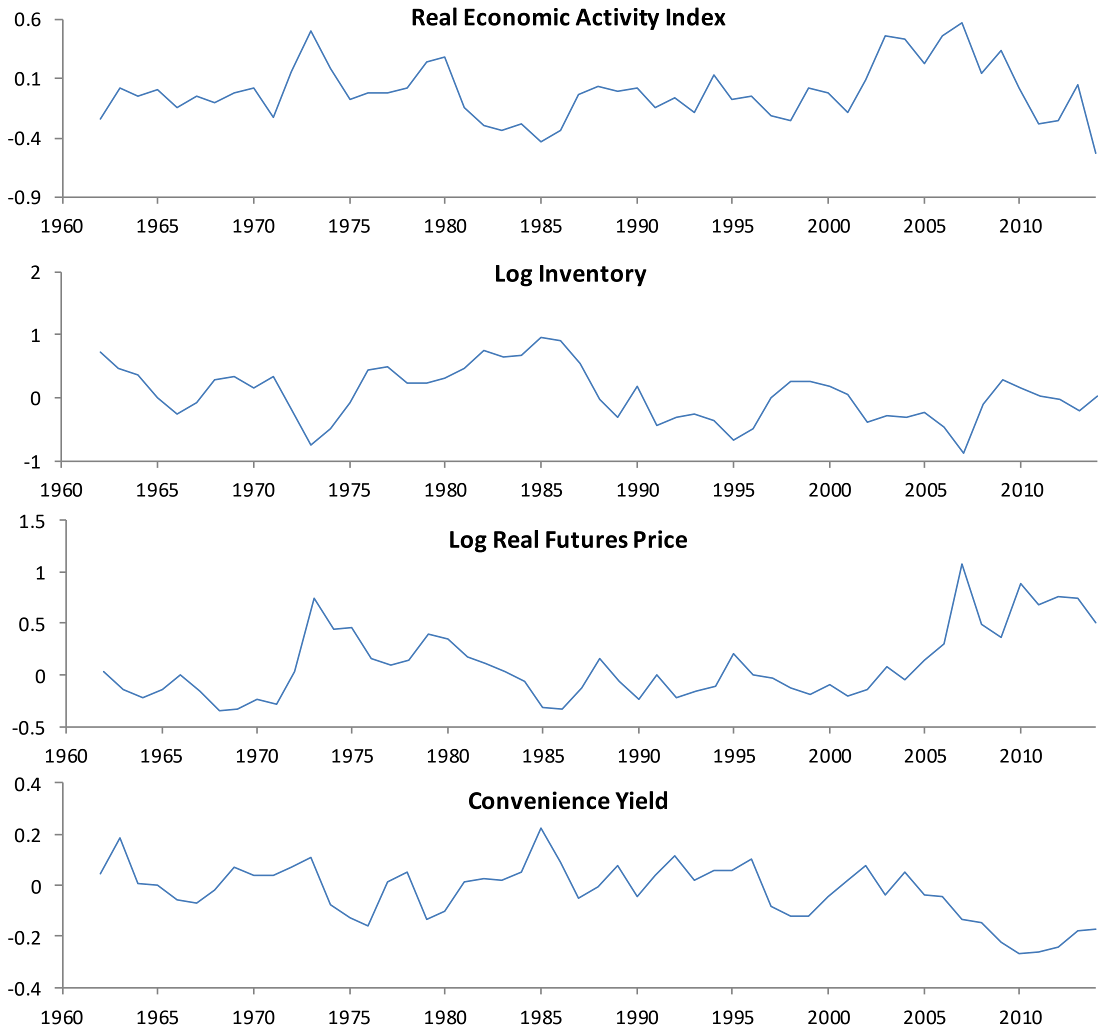

Fig. S15. Detrended data for key variables in wheat model. For clarity, this figure shows linearly detrended series, where we estimate the trend in the pre-RFS period (1961-2005). For the VAR estimation, we use the actual series and include a constant and linear trend in each equation of the model.

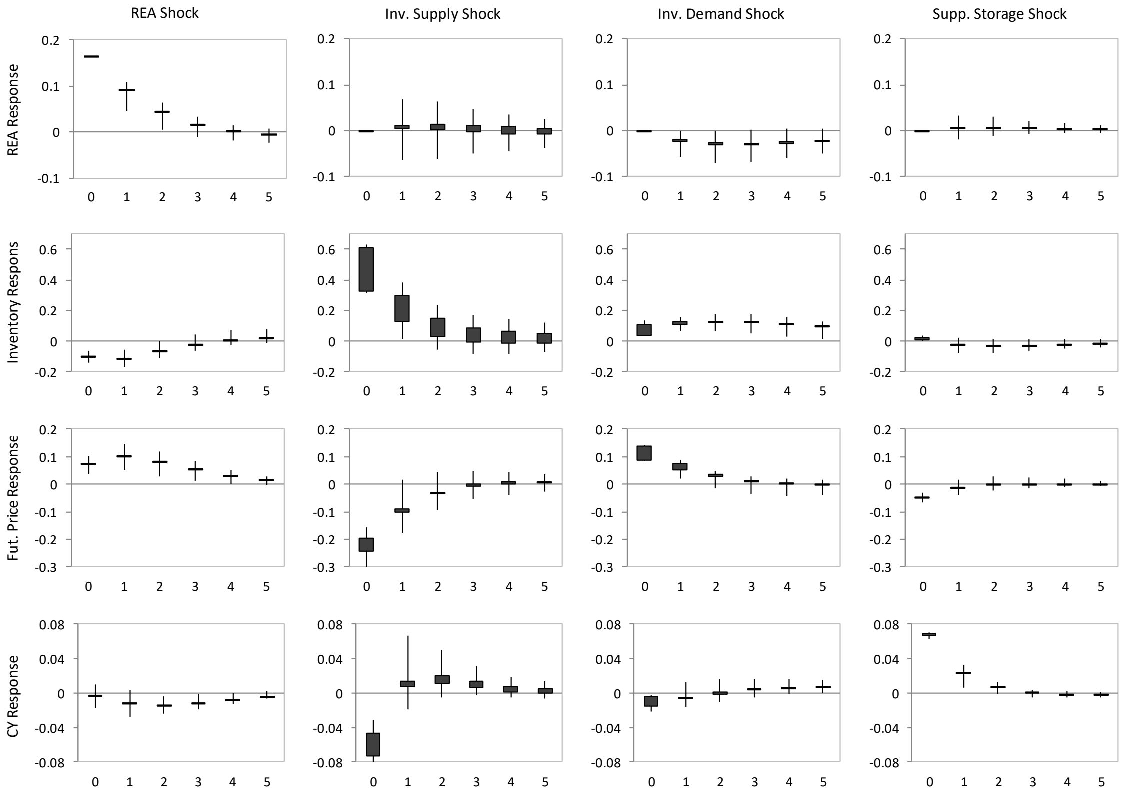

Fig. S16. Impulse response functions for wheat. Responses to one-time one standard deviation shocks for the two-lag model. The dark boxes indicate the range of impulse responses in the identified set. The vertical bars indicate estimated confidence intervals that cover the true parameter with probability greater than 0.90. We obtain these intervals using a recursive-design wild bootstrap following the approach of Carter *et al.* (1).

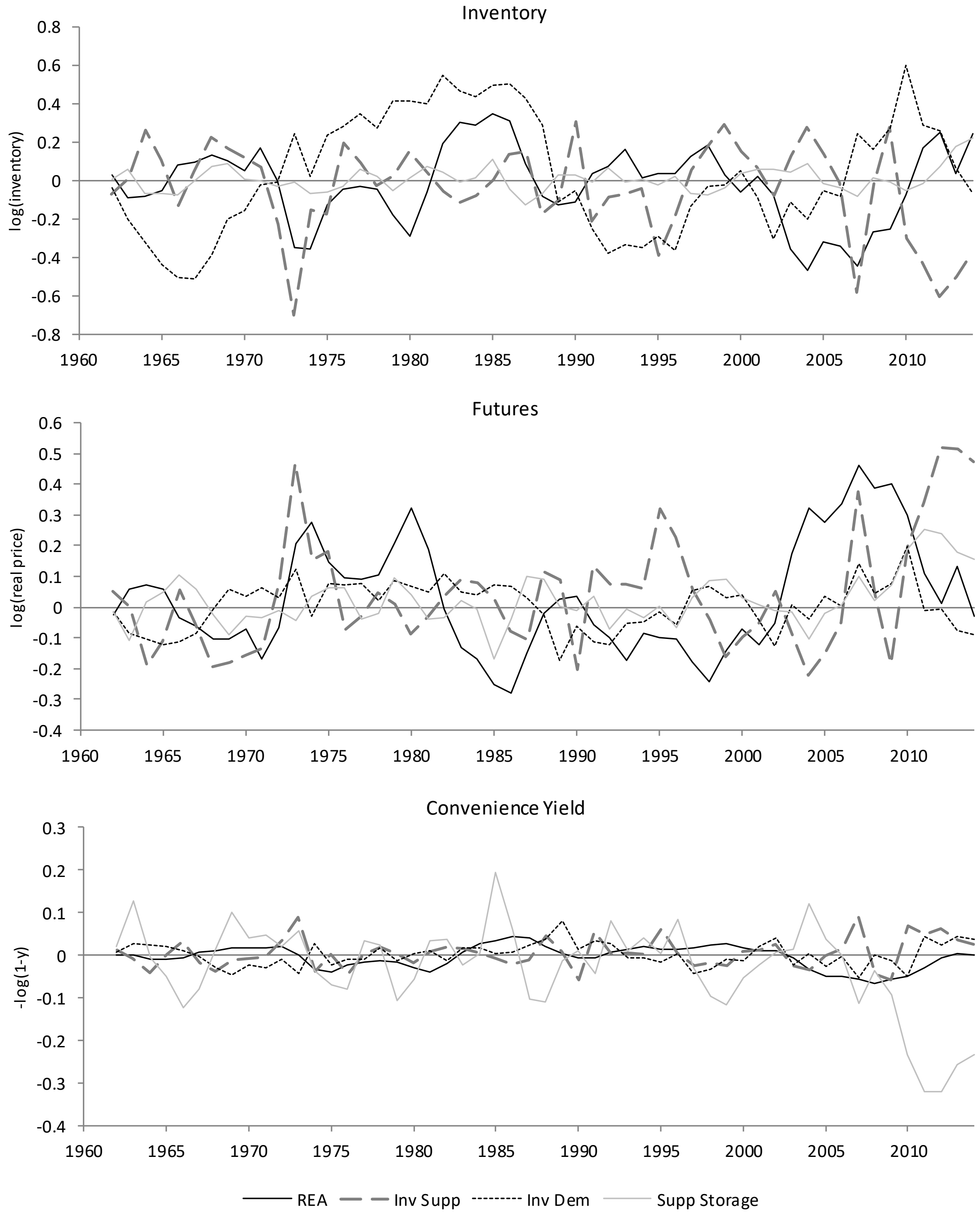

Fig. S17. Historical decomposition for wheat. Figures show contributions of each shock to the relevant series for the one-lag model. The sum of the contributions equals the observed data (net of trend).

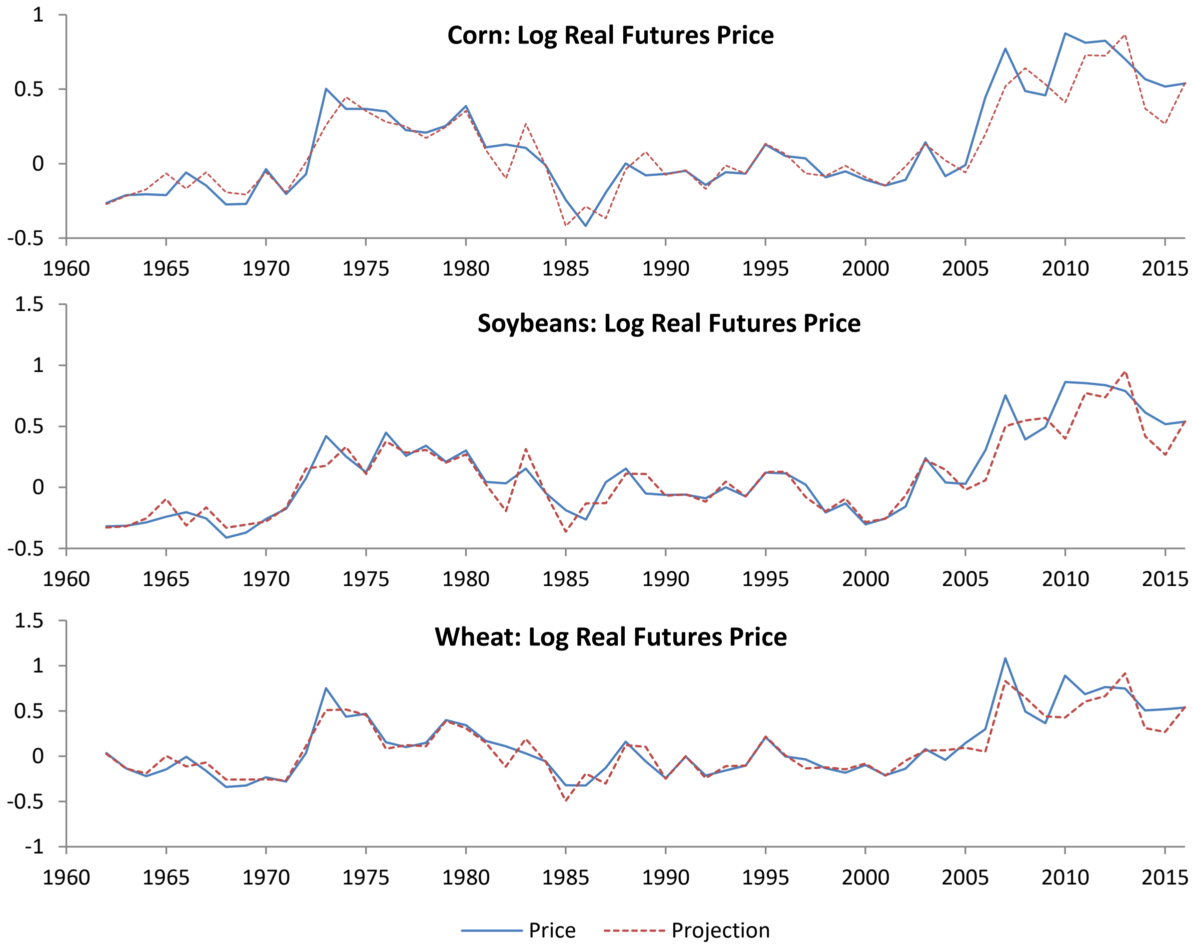

Fig. S18. Predicted crop prices versus observed crop prices over time. Figure shows detrended market prices, as predicted by the VAR model compared to observed prices. Predictions are of the current-year price given current-year values of the other variables and prior-year values of all the variables.

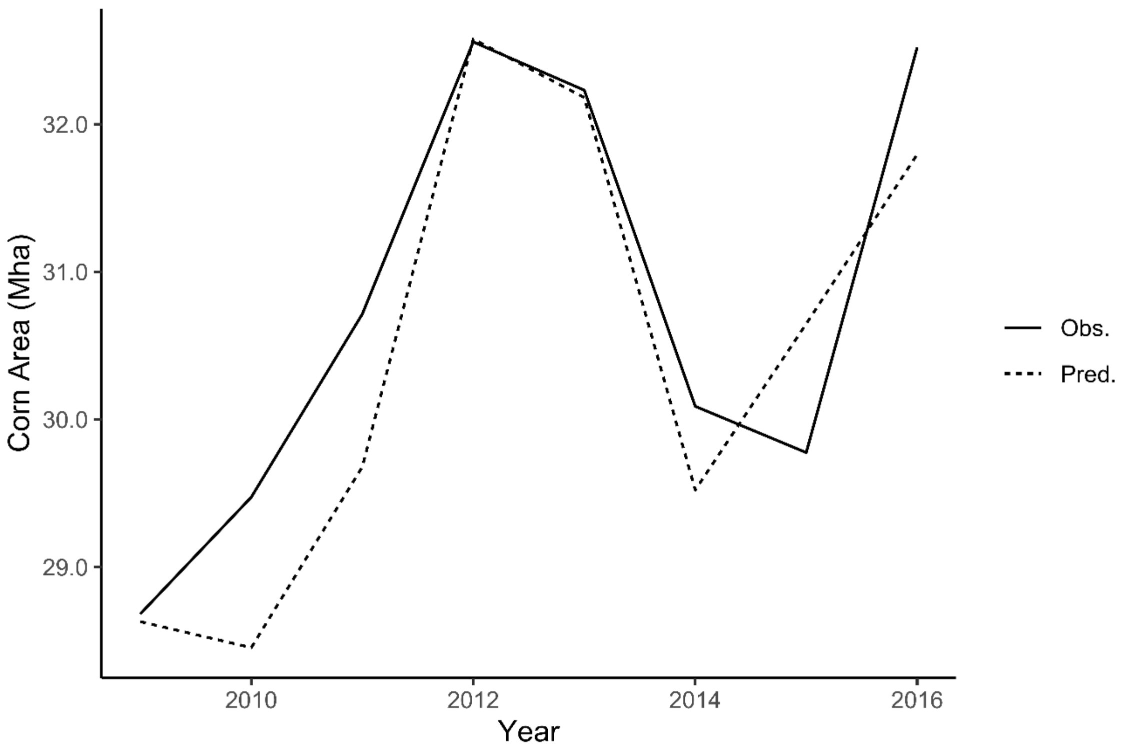

Fig. S19. Predicted corn area versus observed area over time. Figure shows the predicted corn area from the crop rotation model (dashed line) compared to the aggregate area from the Cropland Data Layer (solid line) from 2009 to 2016. We begin the graph in 2009 because the Cropland Data Layer was available for the entire nation starting in 2008 and a one-year lag is used in the modeling. The predicted and observed areas only represent the regions used in our econometric model and not the entire nation.

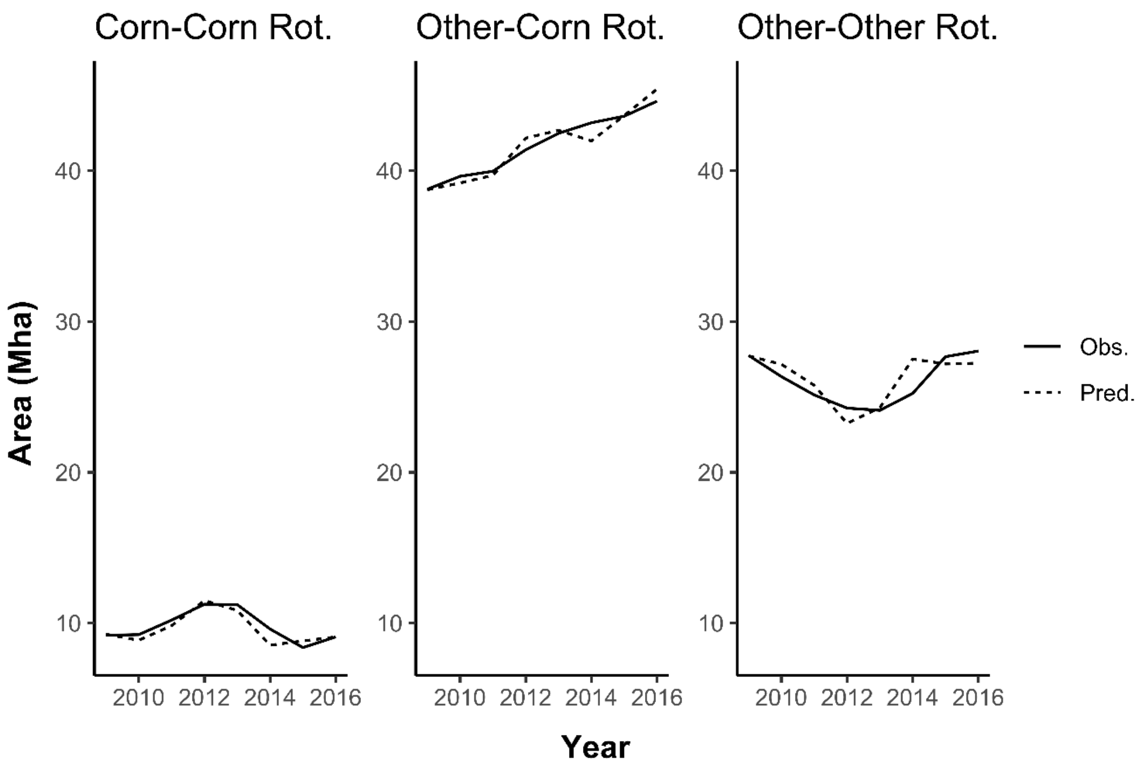

Fig. S20. Predicted area of each crop rotation versus observed area over time. Figure shows the predicted area of each rotation from the econometric model (dashed line) compared to the aggregate area from the Cropland Data Layer (solid line) from 2009 to 2016. We begin the graph in 2009 because the Cropland Data Layer was available for the entire nation starting in 2008 and a one-year lag is used in the modeling. The predicted and observed areas only represent the regions used in our econometric model and not the entire nation.

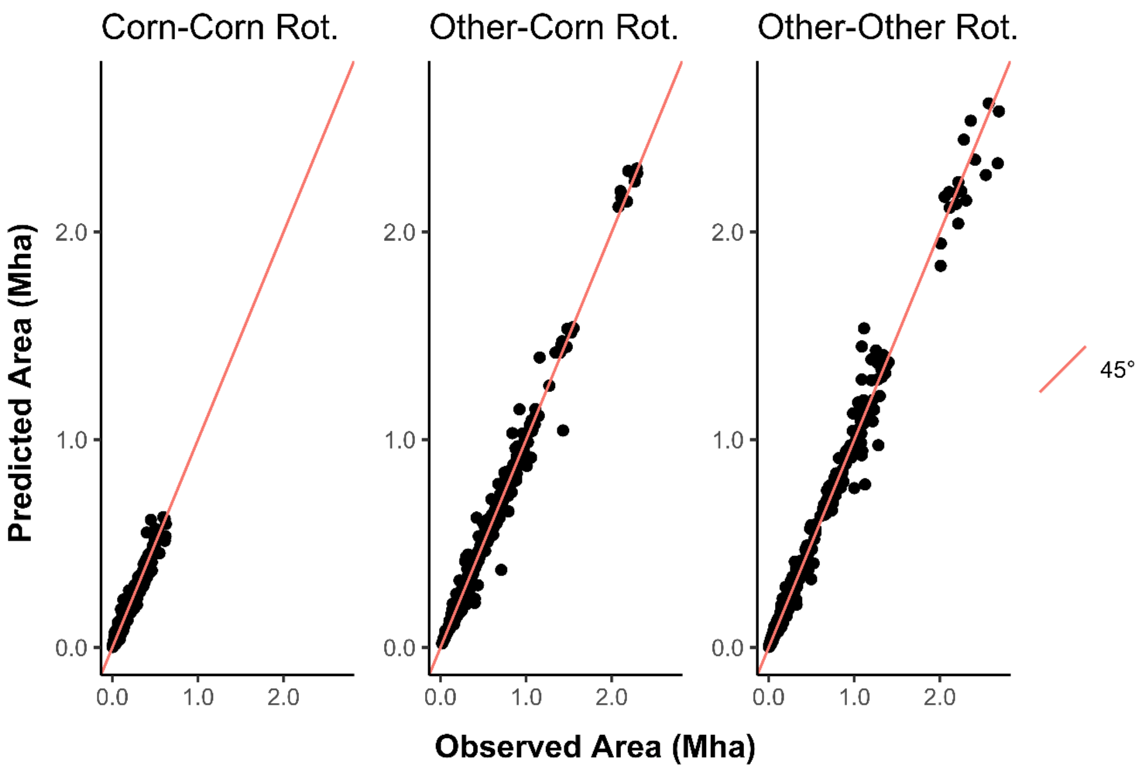

Fig. S21. Predicted area of each crop rotation versus observed area across Major Land Resource Areas. The points in the figure show the predicted area of each rotation from the econometric model and the area from the Cropland Data Layer for each Major Land Resource Area (MLRA) in each year. In other words, each dot represents an MLRA-year pair. The red line starts at the origin with a slope of 1 and indicates the line of perfect fit.

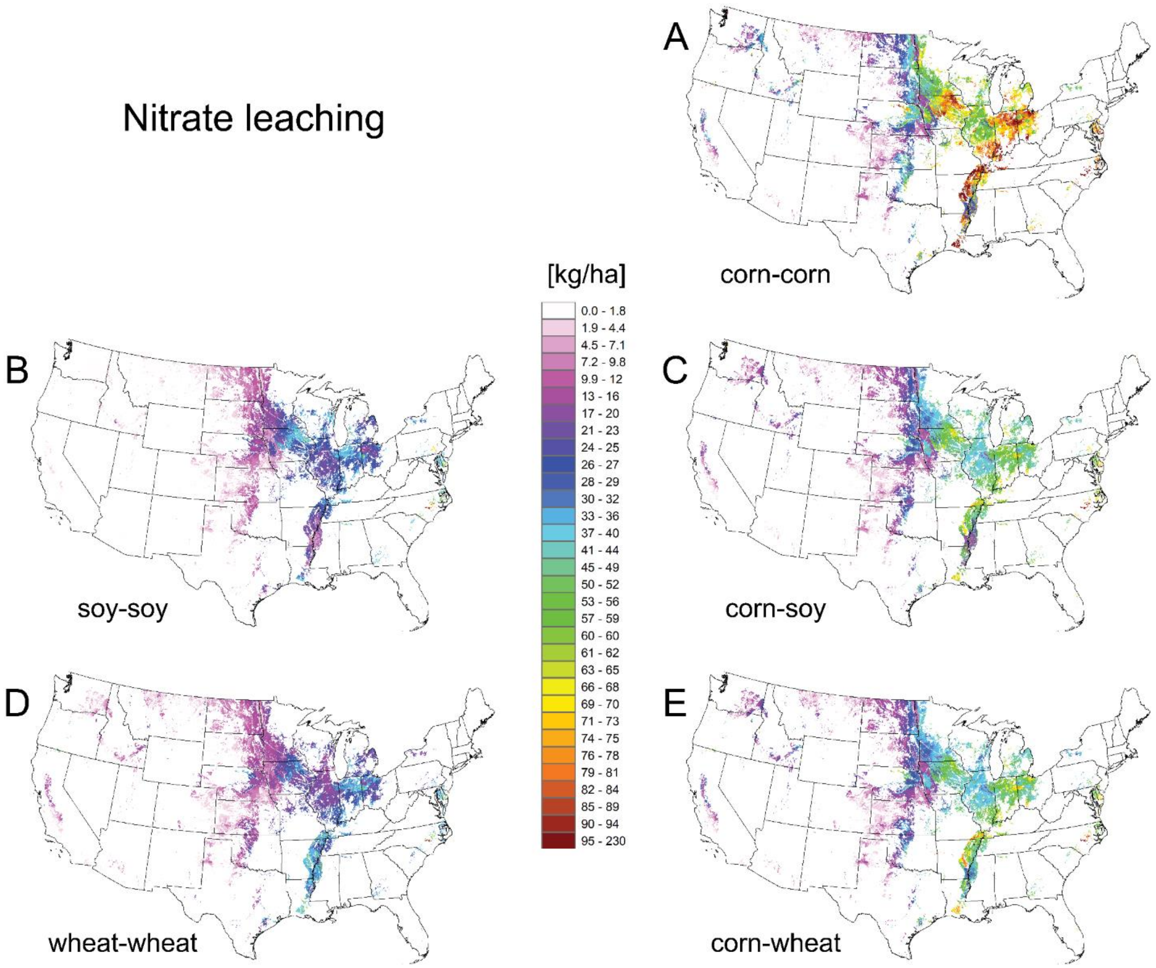

Figure S22. Median annual field-level nitrate leaching. Results shown for the five cropping rotations modeled across all cropland during the 2007-16 time period.

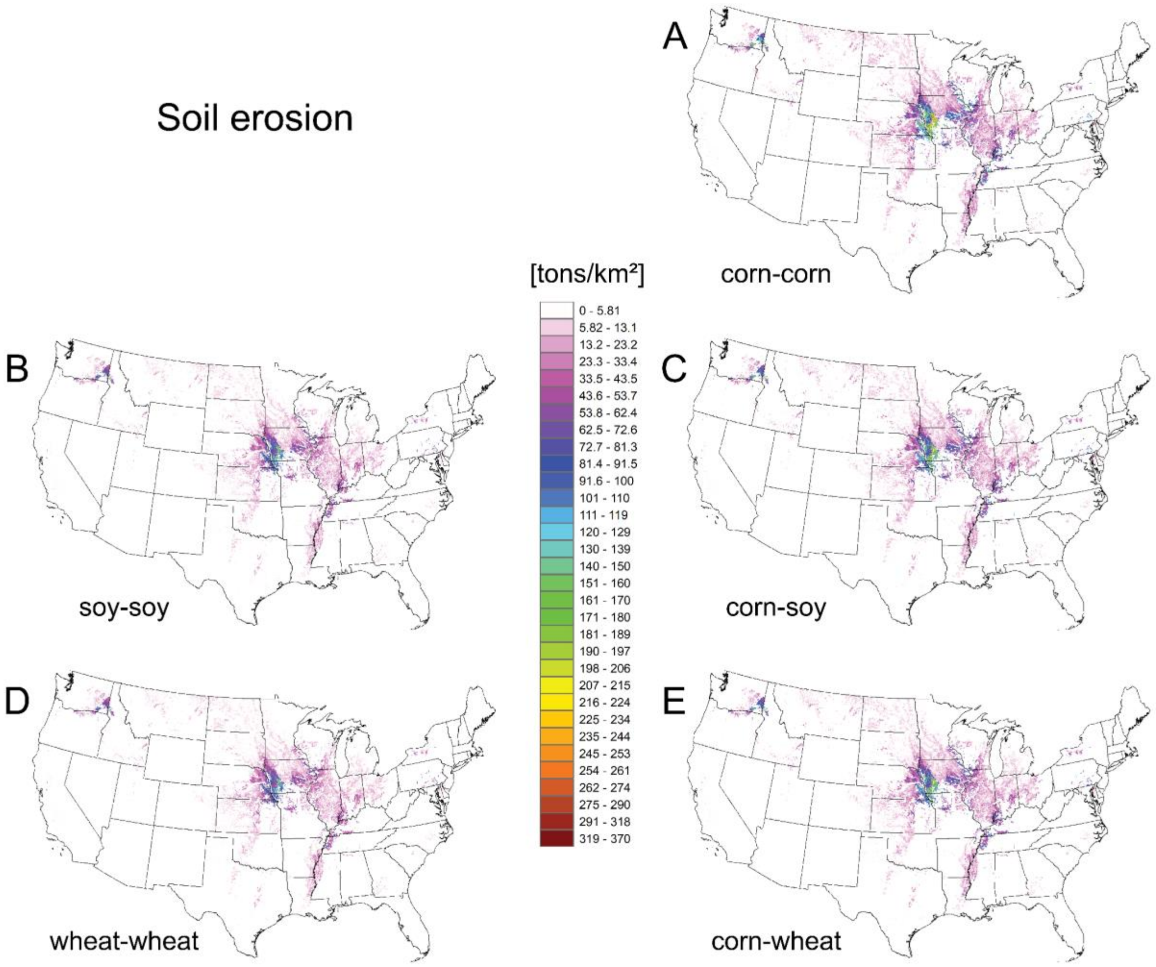

Figure S23. Median annual field-level soil erosion. Results shown for the five cropping rotations modeled across all cropland during the 2007-16 time period.

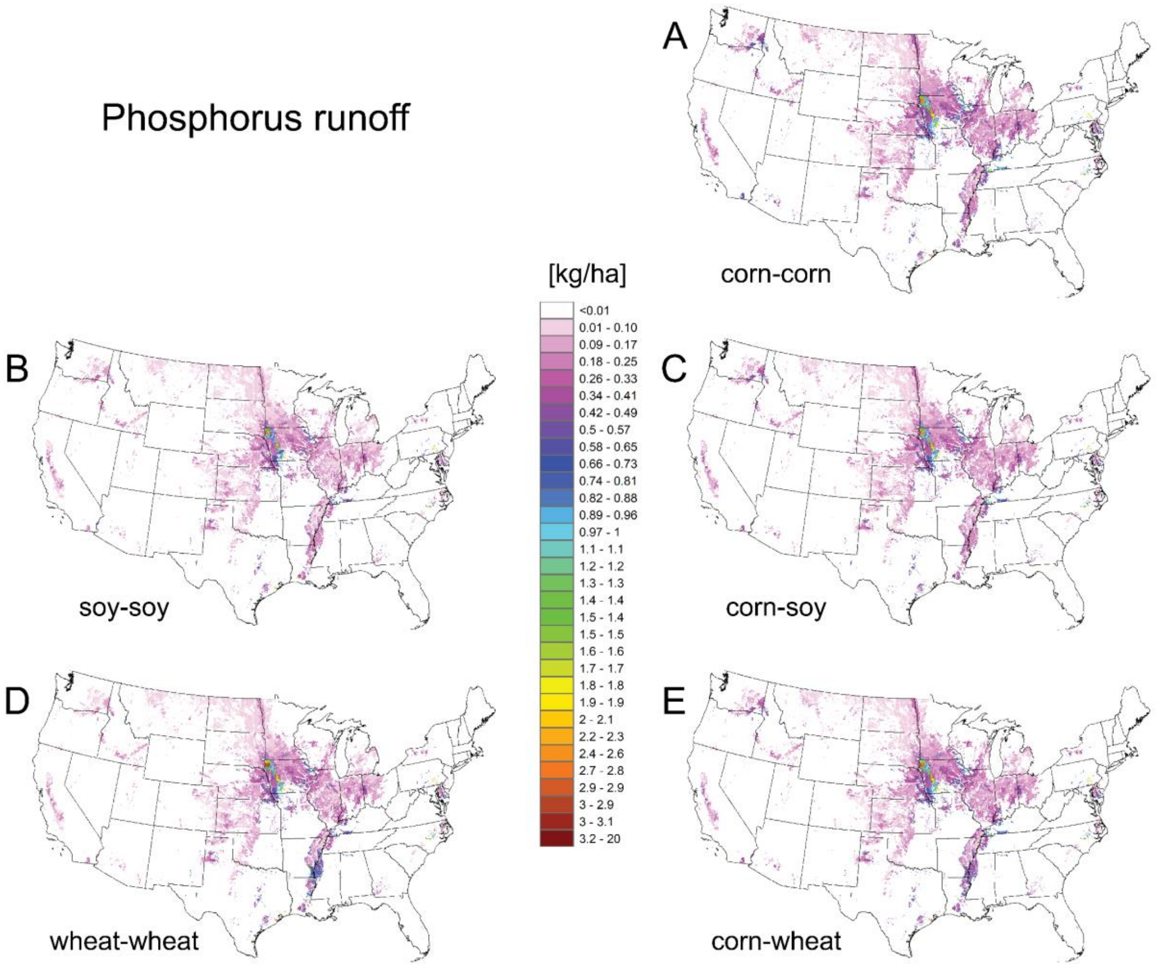

Figure S24. Median annual field-level phosphorus runoff. Results shown for the five cropping rotations modeled across all cropland during the 2007-16 time period.

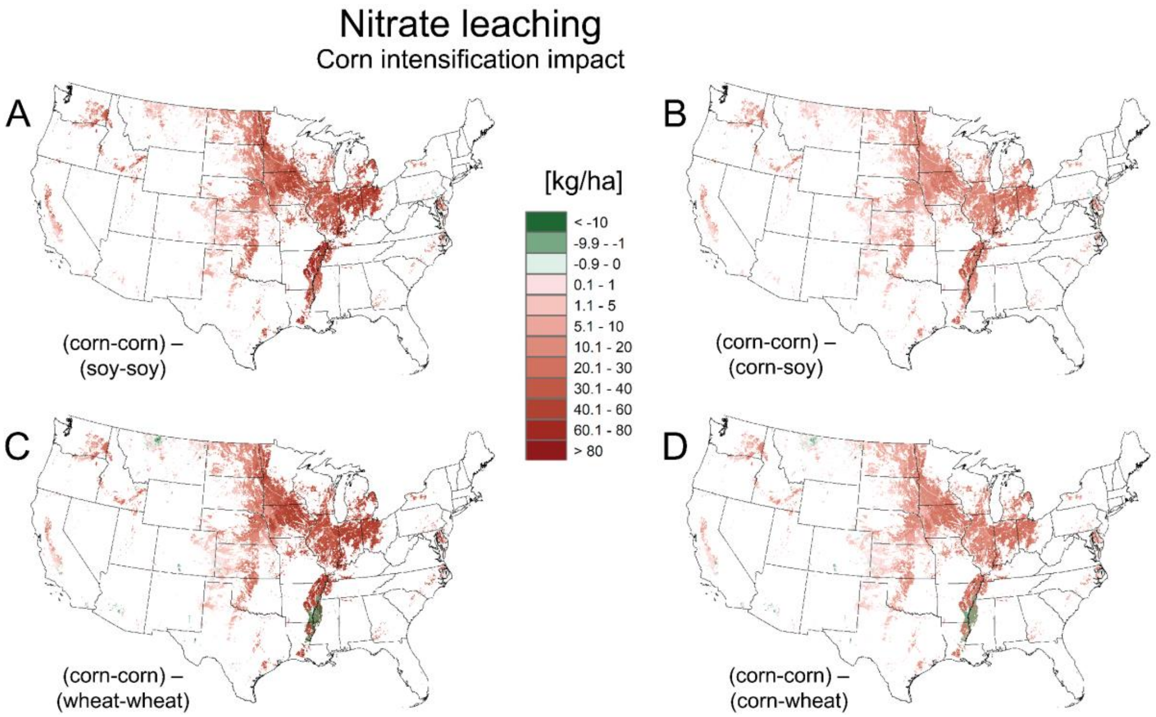

Figure S25. Corn intensification impact. Difference in median annual (2007-16) nitrate leaching loss between continuous corn and other cropping rotations (green indicates continuous corn has less impact; red indicates continuous corn has more impact).

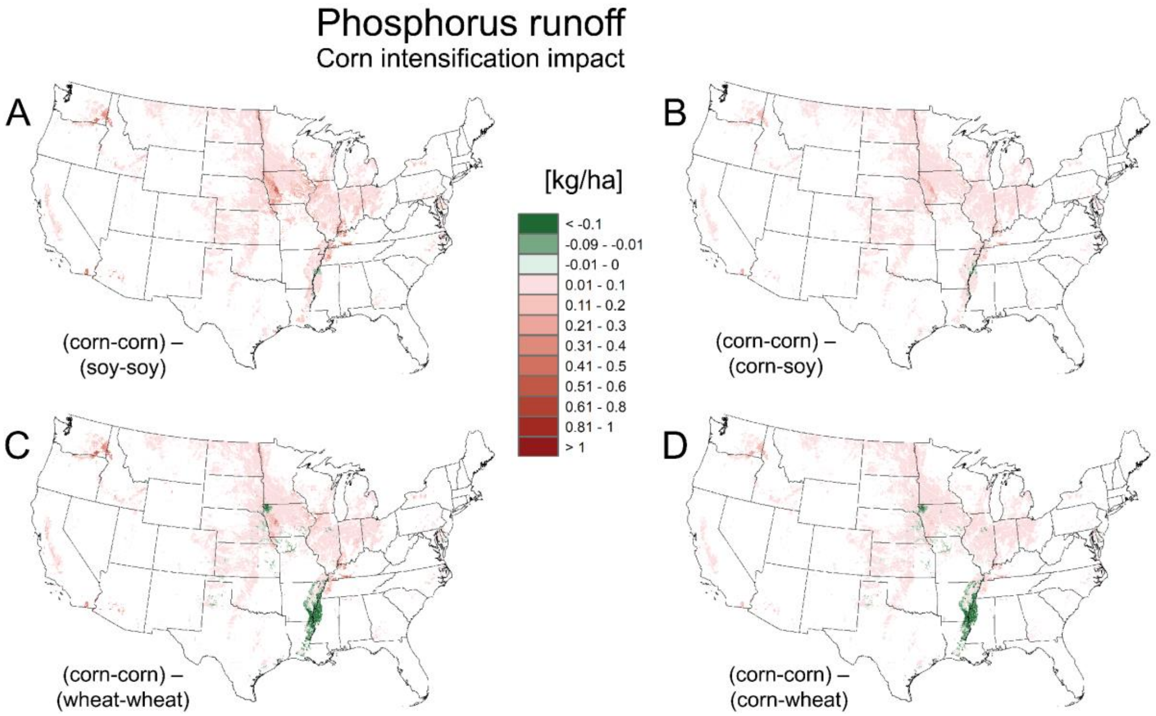

Figure S26. Corn intensification impact. Difference in median annual (2007-16) phosphorus runoff loss between continuous corn and other cropping rotations (green indicates continuous corn has less impact; red indicates continuous corn has more impact).

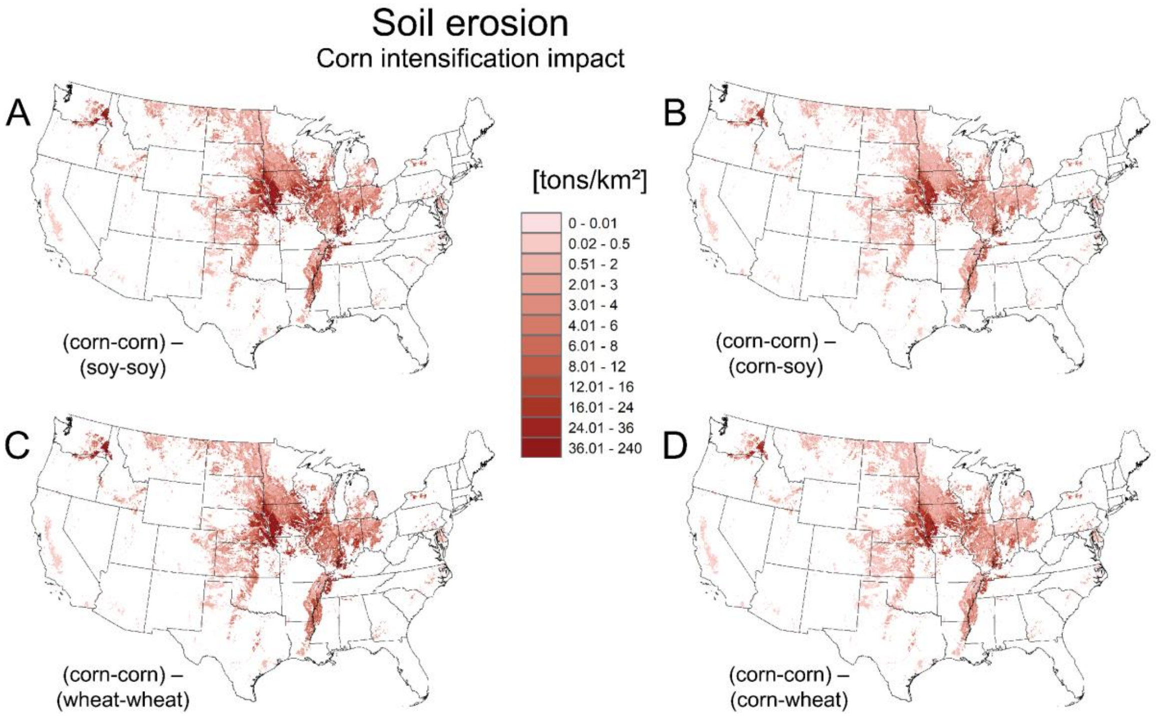

Figure S27. Corn intensification impact. Difference in median annual (2007-16) soil erosion loss between continuous corn and other cropping rotations (green indicates continuous corn has less impact; red indicates continuous corn has more impact)

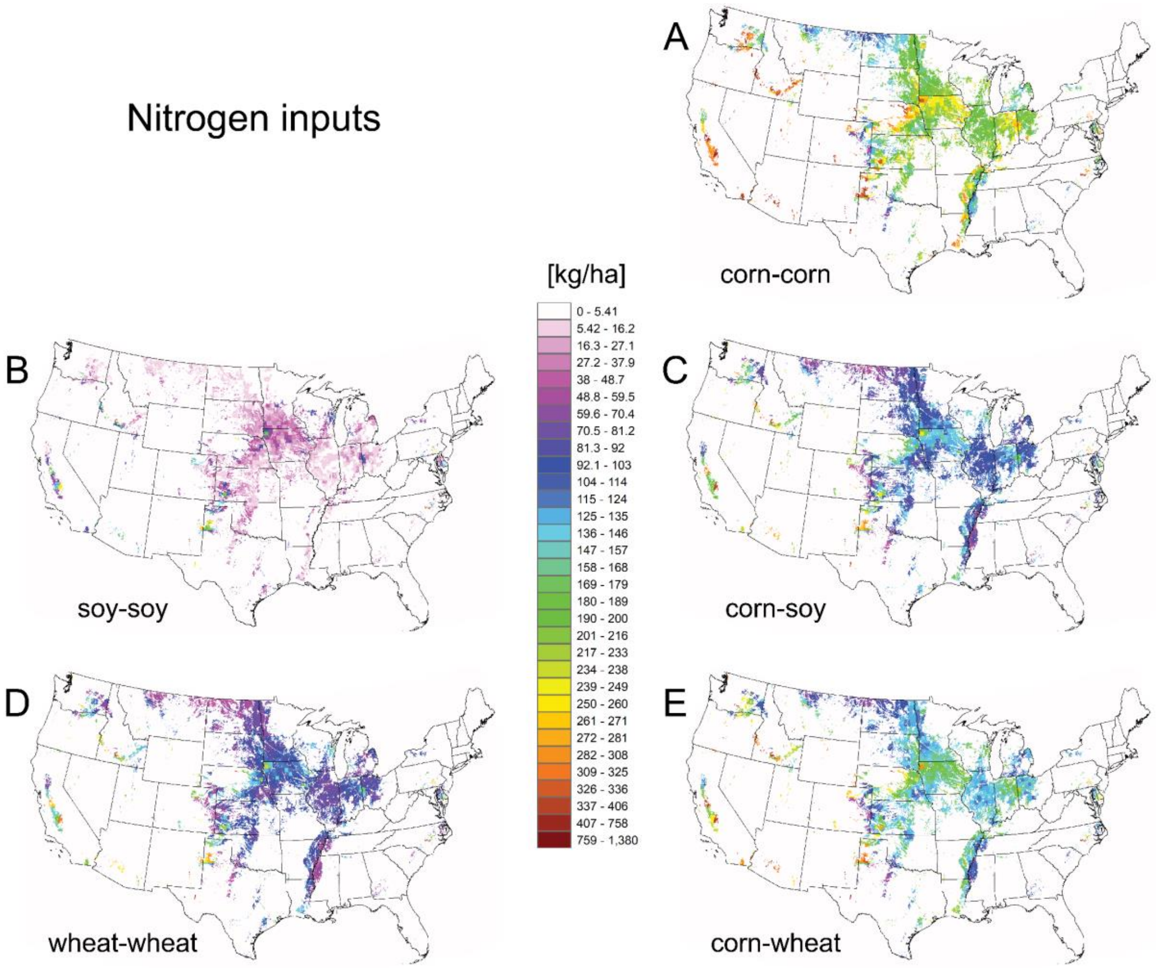

Figure S28. Total nitrogen inputs for scenarios. Mean total (fertilizer and manure) nitrogen inputs over the 2007-16 period for each of the five cropping rotations modeled.

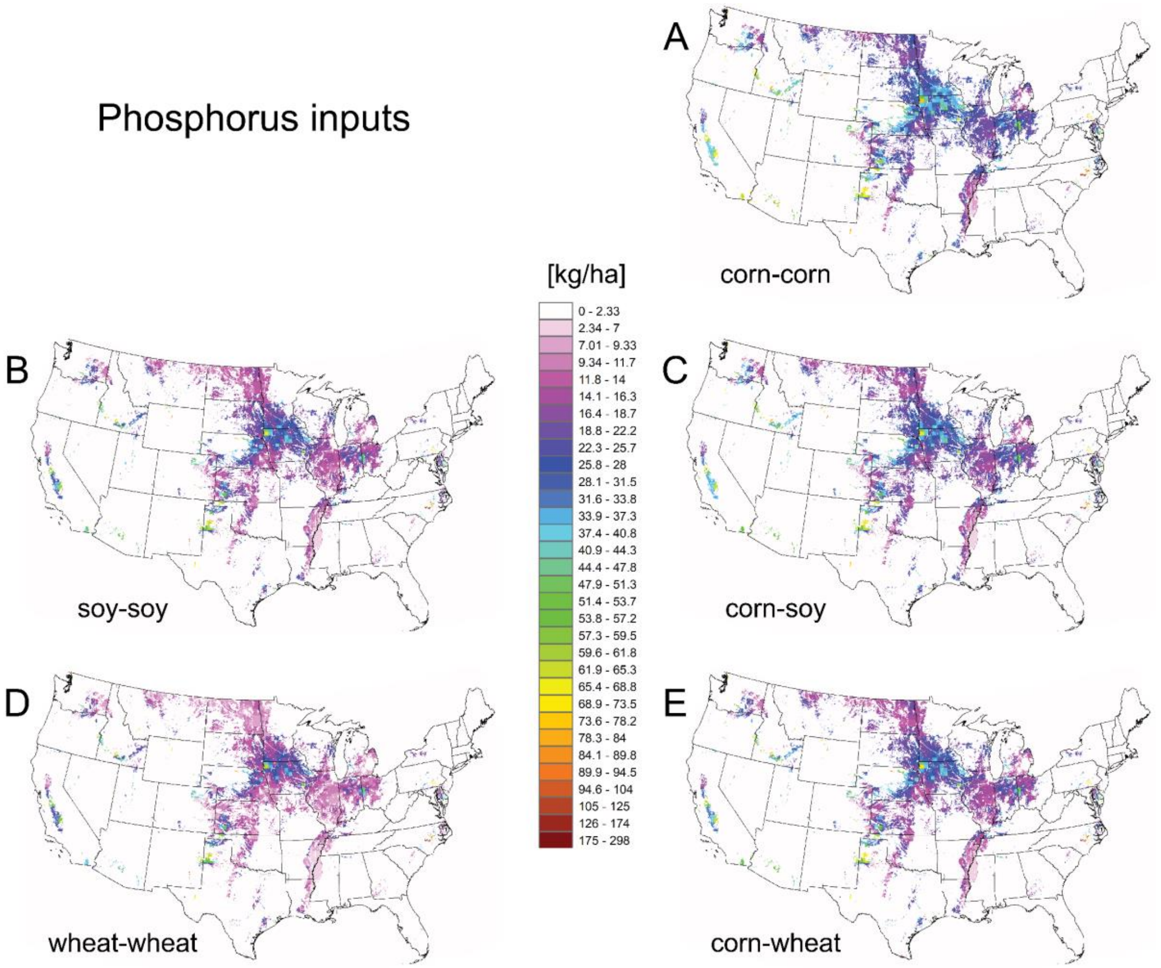

Figure S29. Total phosphorus inputs for scenarios. Mean total (fertilizer and manure) phosphorus inputs over the 2007-16 period for each of the five cropping rotations modeled.

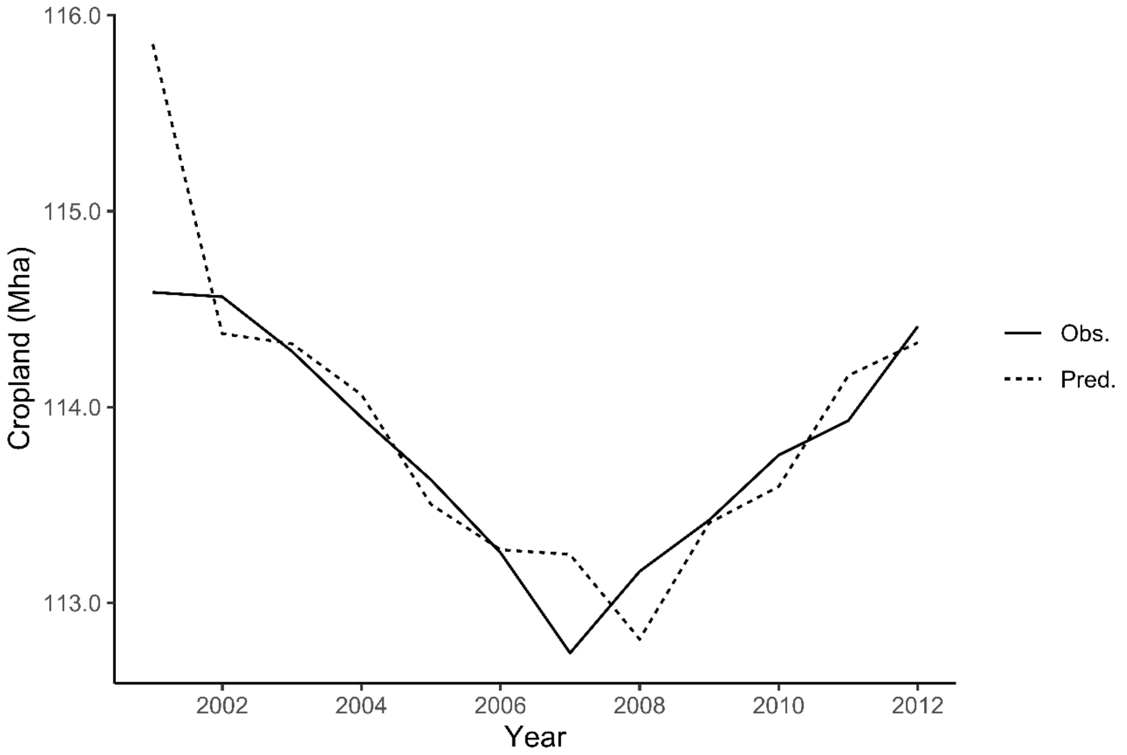

Fig. S30. Predicted area of cropland versus observed area over time. Figure shows the predicted area of cropland from the econometric model for cropland transitions (dashed line) compared to the aggregate area from the National Resources Inventory (solid line) from 2001 to 2012. We begin the graph in 2001 because a one-year lag is used in the modeling. The predicted and observed areas only represent the regions used in our econometric model and not the entire nation.

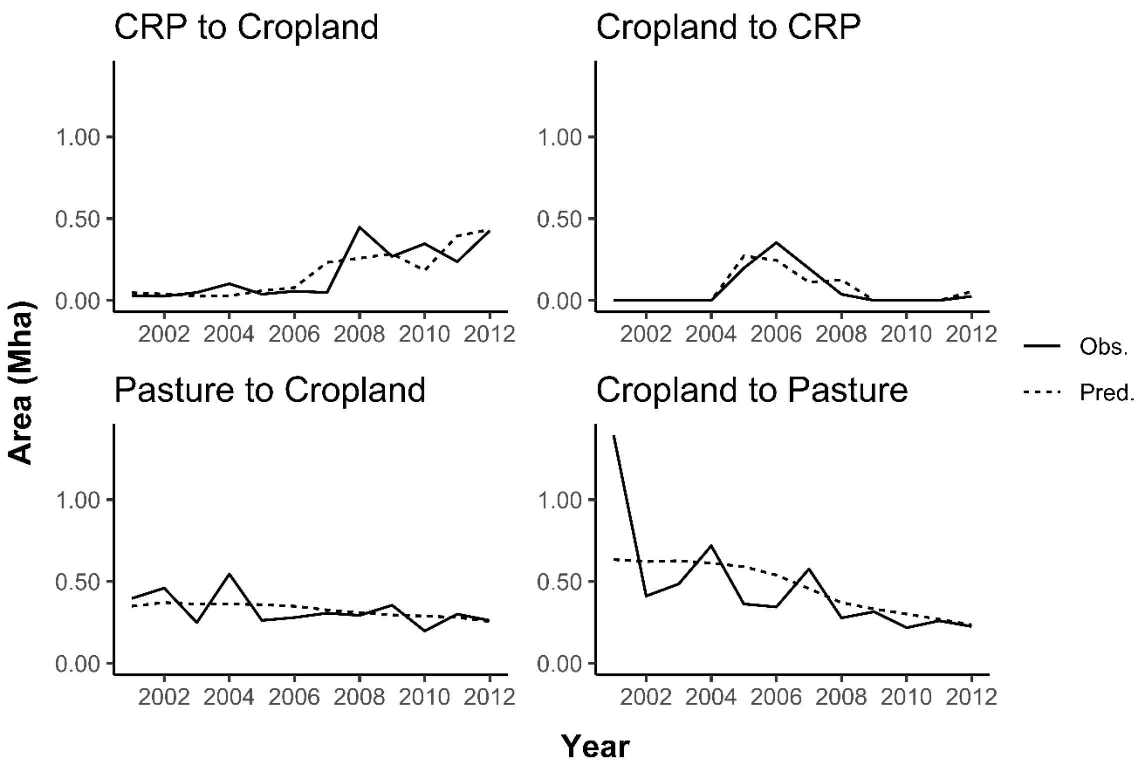

Fig. S31. Predicted area of each cropland transition versus observed area over time. Figure shows the predicted area of each cropland transition from the econometric model (dashed line) compared to the aggregate area from the National Resources Inventory (solid line) from 2001 to 2012. We begin the graph in 2001 because a one-year lag is used in the modeling. The predicted and observed areas only represent the regions used in our econometric model and not the entire nation.

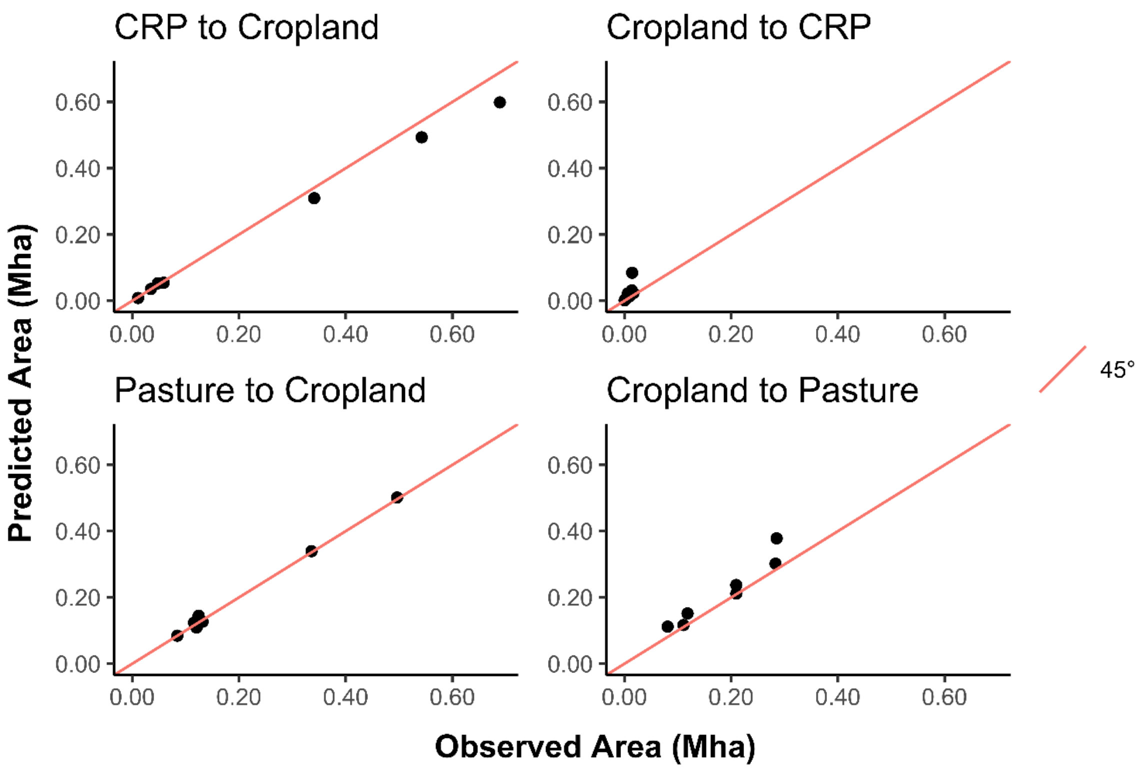

Fig. S32. Predicted area of each cropland transition versus observed area across Land Resource Regions. The points in the figure show the predicted area of each transition from the econometric model and area from the National Resources Inventory (NRI) for each Land Resource Region group modeled. Areas are averaged for transitions between 2007 and 2012. The red line starts at the origin with a slope of 1 and indicates the line of perfect fit.

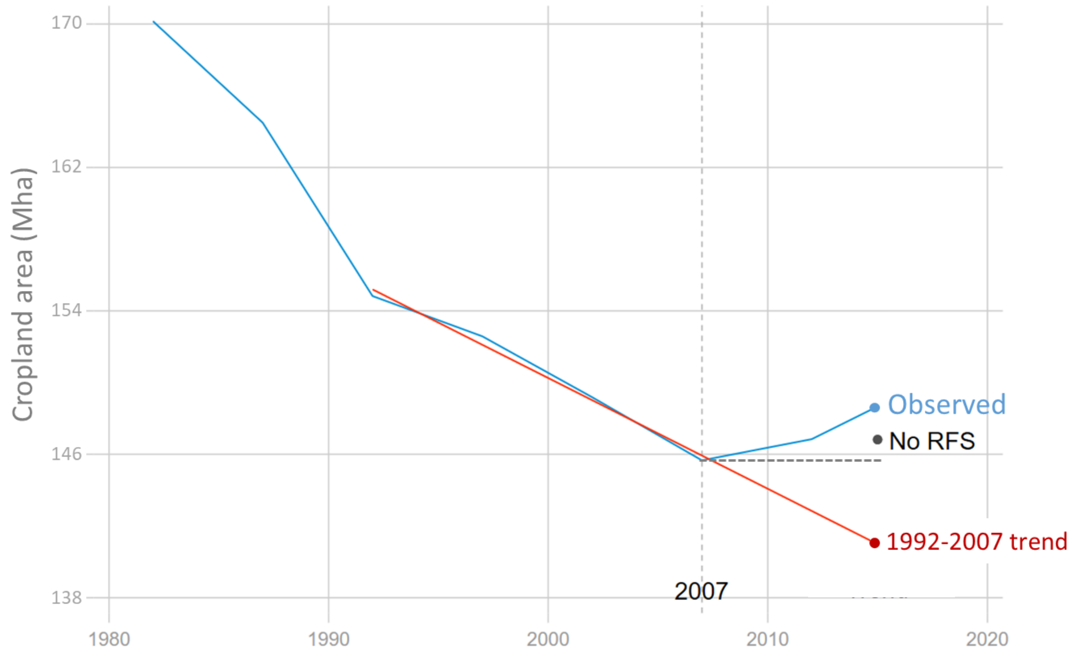

Figure S33. National cropland area over time. The blue line shows total observed cropland area according to the NRI data. The red line represents an extension of the 1992-2007 NRI trend. Point estimate of "No RFS" reflects the actual NRI data minus our estimated impact of the RFS.

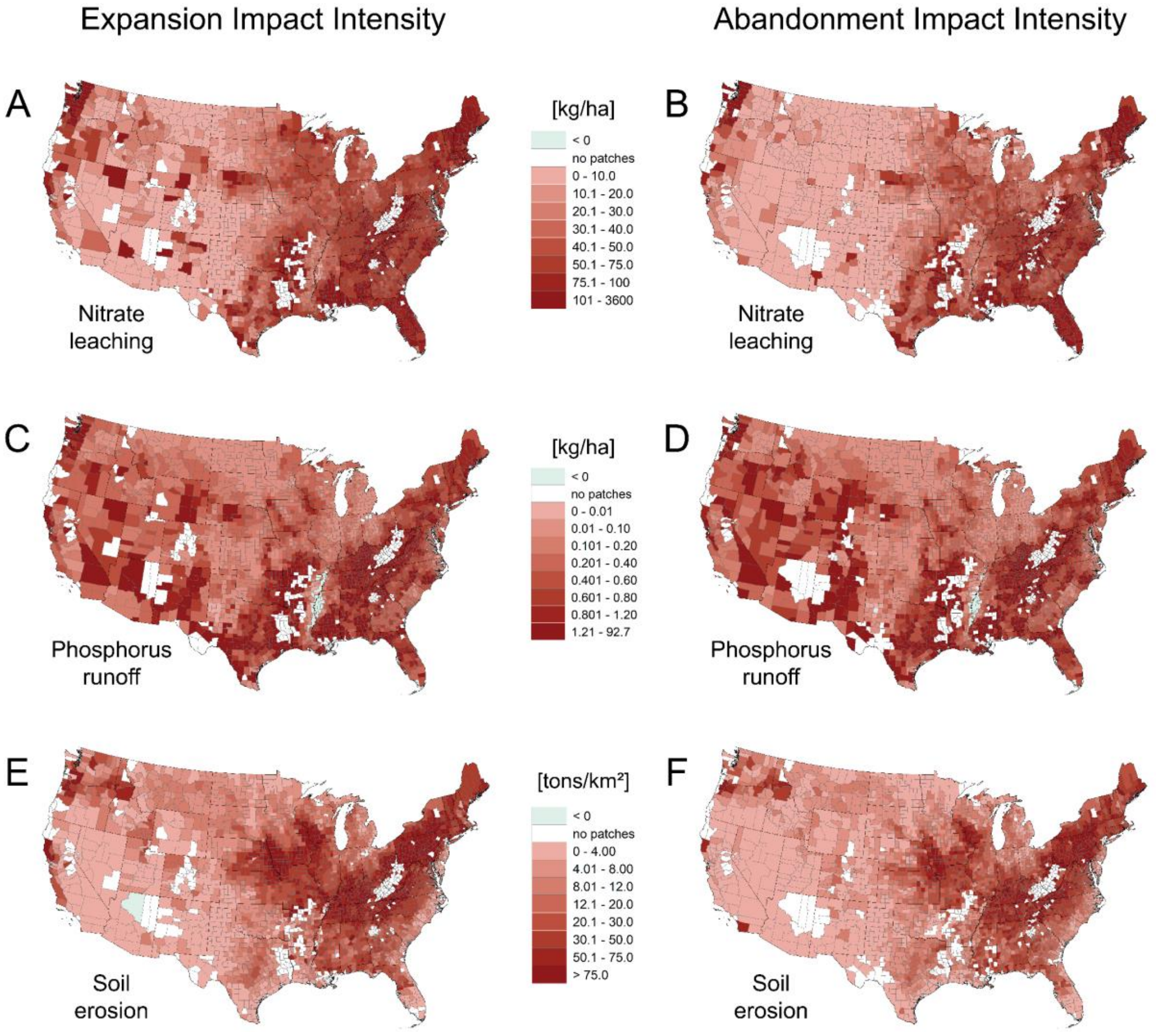

Figure S34. County-averaged impact intensity for nitrate leaching, phosphorus runoff, and soil erosion. Results separated into land use patches that underwent cropland expansion (left column) and abandonment (right column). The impact intensities for all maps (A-F) represent the differences in values for cropland minus noncropland.

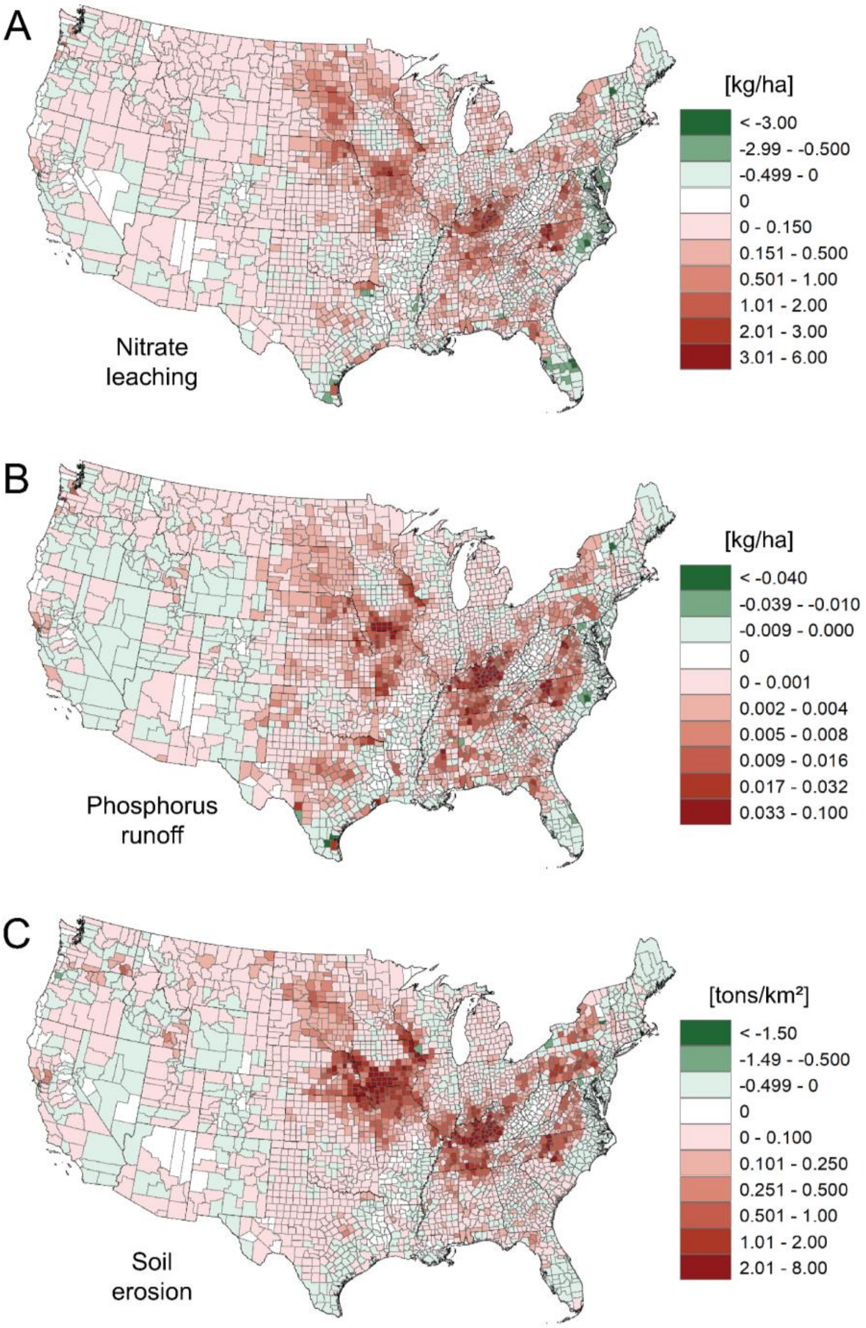

Figure S35. Net impacts for nitrate leaching, phosphorus runoff, and soil erosion for all cropland expansion and abandonment, 2008-16. Net impacts reflect the total impacts (intensity x area of conversion) from cropland expansion minus those from abandonment within each county. Net impact values are divided by total county area for normalization and visualization. Note that for this figure only, results reflect impacts from all recent land conversion, not only the subset due to the RFS, and are included to document the underlying trends and data used to estimate RFS-specific water quality impacts.

Tables S1-S23.

	Corn		Soybeans		Wheat	
Dollars per bushel						
2001-05	2.15		5.96		4.03	
2006-10						
Observed	3.81		9.67		6.52	
BAU	2.90		8.11		5.45	
(95% CI for BAU)	2.24	3.65	5.61	10.56	4.07	6.39
2006-10 percent increase relative to …						
… 2001-05	77%		62%		62%	
… BAU	31%		19%		20%	
(95% CI for BAU)	70%	5%	72%	-8%	60%	2%

Table S1: Observed vs. business-as-usual (BAU) spot prices. The BAU prices are produced from the model in Carter *et al.* (1) using data updated through the 2016-17 crop year. The model projects the natural log of prices. To obtain the BAU value, we took the average projected difference between the observed and BAU log prices during 2006-10. These differences were 0.27 for corn, 0.18 for soybeans, and 0.20 for wheat, which correspond to 31%, 19%, and 20% respective differences and imply that the observed prices were 31%, 19%, and 20% above the BAU for the three commodities. The point estimates come from the point identified parameters in the model and the confidence intervals are generated from the identified set (see **Tables S10-S13**).

	NRI total cropland Δ		Due to RFS (this study)		RFS contribution	
	2007-15 (Mha)	Annual Ave. (Mha)	2008-16 (Mha)	Annual Ave. (Mha)	% of NRI	%Δ from BAU
Expansion	8.68	1.09	1.80	0.22	20.7%	26.1%
Abandonment	5.64	0.71	-0.35	-0.04	-6.3%	-5.9%
Net	3.04	0.38	2.15	0.27	70.7%	240.9%

Table S2. Relative contribution of the RFS to cropland expansion and abandonment. Percent contributions of the RFS calculated from mean annual changes to account for different endpoints between this study and the NRI dataset used for comparison.

Emissions	Total	Annualized	GHG Intensity		
	Gg CO_2e	Gg CO_2e / yr	g CO_2e / L	g CO_2e / MJ	kg CO_2e / mmBtu
Ecosystem carbon	397,659	13,255	636.67	29.66	31.29
--Cropland expansion	*320,380*	*10,679*	*512.94*	*23.90*	*25.21*
--Forgone abandonment	*77,279*	*2,576*	*123.73*	*5.76*	*6.08*
N2O	-	4,052	194.60	9.07	9.57
--Crop rotations Δ	-	*2,773*	*133.20*	*6.21*	*6.55*
--Cropland extent Δ	-	*1,279*	*61.42*	*2.86*	*3.02*
Total domestic LUC	**-**	17,307	831.27	38.73	40.86

Table S3: Greenhouse gas (GHG) emissions by source. Annualized ecosystem carbon emissions based on a 30-year amortization period (73). GHG intensities calculated using a 20.82 billion liter modeled change in annual production, an ethanol heating value of 21.46 MJ / L, and a conversion factor of 947.82 MJ / Btu.

		Corn	Soybeans	Wheat
Global Commodity Demand (X)	variable	Real economic activity index	Real economic activity index	Real economic activity index
	Source	Kilian (2009)	Kilian (2009)	Kilian (2009)
	timing	March	March	March
Inventory (I)	variable	Total ending stocks (bu)	Total ending stocks (bu)	Total ending stocks (bu)
	source	USDA	USDA	USDA
	timing	September	September	June
Futures Price (F)	variable	CBOT Dec contract	CME Nov contract	CBOT Dec contract (1960-1976), KCBOT Dec contract (1977-2017)
	source	quandl	quandl	quandl
	timing	average daily price in March	average daily price in March	average daily price in March
Spot Price (S)	variable	Central IL cash bid	Central IL cash bid	St Louis SRW cash bid (1960-1976), Kansas City HRW cash bid (1977-2017)
	source	USDA AMS	USDA AMS	USDA AMS
	timing	average daily price in March	average daily price in March	average daily price in March

Annual Timeline

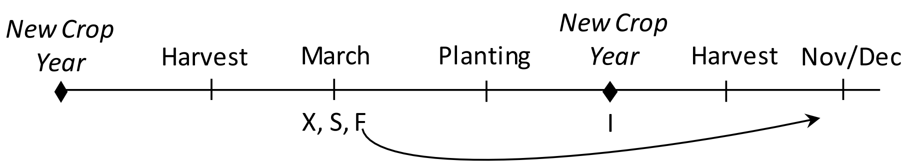

Table S4: Data used in price impact model. All variables measured annually from 1960-2017. All prices deflated by the March Consumer Price Index for all items. The price and inventory variables enter the model in logs.

Value	Vegetation Type in AgroIBIS
1	Tropical broadleaf evergreen tree
2	Tropical broadleaf drought-deciduous tree
3	Warm-temperate broadleaf evergreen tree
4	Temperate conifer evergreen tree
5	Temperate broadleaf cold-deciduous tree
6	Boreal conifer evergreen tree
7	Boreal broadleaf cold-deciduous tree
8	Mixed Forest
9	Savanna
10	Grassland
11	Dense Shrubland
12	Open Shrubland
13	Tundra
14	Desert
15	Polar Desert
16	Corn
17	Soybean
18	Wheat
19	Alfalfa
20	Hay
21	Pasture
22	Developed / High Intensity (Turf Grass)
23	Developed / Medium Intensity (Turf Grass)
24	Developed / Low Intensity (Turf Grass)
25	Developed / Open Space (Turf Grass)
26	Herbaceous Wetland
27	Woody Wetland
30	Barren
98	Open Water

Table S5. Vegetation types simulated in the agroecosystem modeling.

StartYear	EndYear	Dataset Name	Spatial Resolution	Source
N/A	N/A	Potential Vegetation	5 arc-minute	Ramankutty and Foley [1999]
1938	1992	FORE-SCE BACKCAST	250 meter	Sohl *et al*. [2016]
1993	1998	FORE-SCE HISTORICAL	250 meter	Sohl *et al*. [2014]
2001	2001	USGS NLCD 2001	30 meter	Homer *et al*. [2007]
2006	2006	USGS NLCD 2006	30 meter	Fry *et al*. [2011]
2011	2011	USGS NLCD 2011	30 meter	Homer *et al*. [2015]
2008	2016	USDA-NASS CDL	30 meter	USDA [2017]

Table S6. Land cover datasets used for the agroecosystem modeling.

Variable/Statistic Name
Federal Information Processing Standards (FIPS) county code
Cropland harvested, total area
Cropland, irrigated area
Corn harvested area
Soybeans harvested area
Wheat harvested area
Alfalfa harvested area
Hay harvested area
Non-simulated crops area
Pasture area
Irrigated pasture area
Fraction of cropland harvested that was irrigated
Fraction of pasture that was irrigated

Table S7. Variables and statistics derived from Ag Census to map historic land cover.

Start Year	End Year	Fertilizer	Manure	Source Citation
1945	1985	X		Alexander and Smith, 1990
1987	2006	X		Gronberg and Spahr, 2012
2007	2012	X		Brakebill and Gronberg, 2017
1982	1997		X	Ruddy *et al*., 2006
2002	2002		X	Mueller and Gronberg, 2013
2007	2012		X	Gronberg and Arnold, 2017

Table S8. Sources of county-level estimates of N and P inputs to the landscape.

Crop/Cover Type	Recommended Fertilizer N Rate [lb/acre]	Ratio to Corn (*R*)	Recommended Fertilizer P_2O_5 Rate [lb/acre]	Ratio to Corn (*R*)	Source Citation (state)
Corn	180	1	80	1	Laboski *et al*. 2012 (WI)
Soy	0	0	50	0.63	Laboski *et al*. 2012 (WI)
Wheat	70	0.39	35	0.44	Laboski *et al*. 2012 (WI)
Alfalfa	5	0.03	68	0.85	Laboski *et al*. 2012 (WI)
Non-alfalfa hay	100	0.56	55	0.69	Laboski *et al*. 2012 (WI)
Pasture	100	0.56	55	0.69	Laboski *et al*. 2012 (WI)
Other crop*	150	0.83	60	0.75	Mylavarapu *et al*. 2015 (FL)

Table S9. Recommended fertilizer N and P application rates, and ratios of rates to corn rate used in mapping. *Rates are an average for sorghum and cotton

Equation	*REA*	*Inventory*	*Futures*	*Conv. Yield*
	Reduced Form Estimates: $A^{-1}B_1$			
REA_{t-1}	0.52* (0.12)	-0.24 (0.29)	0.04 (0.11)	-0.07 (0.06)
$Inventory_{t-1}$	-0.02 (0.04)	0.71* (0.11)	0.04 (0.04)	-0.04* (0.02)
$Futures_{t-1}$	-0.04 (0.10)	0.90* (0.30)	0.71* (0.10)	-0.06 (0.04)
$Conv.\ Yield_{t-1}$	0.86* (0.26)	1.87* (0.53)	0.33 (0.44)	-0.01 (0.11)
Constant	0.11 (0.36)	-0.20 (1.16)	0.37 (0.30)	0.60 (0.19)
Trend	0.000 (0.003)	0.035* (0.013)	-0.011* (0.004)	0.000 (0.001)
	A Matrix: imposing $\alpha_{23} = 4.4 - 1/(\alpha_{32} + \alpha_{42}(1+\alpha_{34}))$			
REA	1	0	0	0
Inventory Supply	0.65	1	-0.50	-0.50
Inventory Demand	-0.44	0.17	1	-0.12
Supply of Storage	-0.10	0.09	0	1
	A Matrix: Identified Set			
REA	1	0	0	0
Inventory Supply	[0.49, 3.04]	1	[-4.25,-0.25]	[-4.25,-0.25]
Inventory Demand	[-0.44,-0.37]	[0.15, 0.24]	1	[-0.46, -0.16]
Supply of Storage	[-0.10, -0.08]	[0.09, 0.15]	0	1
	A Matrix: >90% Confidence Interval			
REA	1	0	0	0
Inventory Supply	[-0.04, 4.89]	1	[-6.60, -0.25]	[-6.60, -0.25]
Inventory Demand	[-0.52,-0.25]	[0.09, 0.30]	1	[-0.93, 0.12]
Supply of Storage	[-0.18, 0.02]	[0.05, 0.22]	0	1

Table S10. Soybean VAR parameter estimates. Sample range: 1961–2005; standard errors in parentheses; * indicates significance at 5%; model selection criteria values are AIC_C = -648.86 and BIC = -620.31; for the two-lag model, we obtain AICc = -640.24 and BIC = -583.15, so the one-lag model is favored. We obtain the confidence intervals using a recursive-design wild bootstrap (1).

	2006-07	2007-08	2008-09	2009-10	2010-11	Average
No Inventory-Demand Shocks						
Inventory	0.00	0.00	0.00	0.00	0.00	0.00
Fut. Price	0.00	0.00	0.00	0.00	0.00	0.00
Conv. Yield	0.00	0.00	0.00	0.00	0.00	0.00
Cash Price	0.00	0.00	0.00	0.00	0.00	0.00
No Inventory-Demand or -Supply Shocks						
Inventory	0.61	-0.28	-0.85	-0.66	-0.35	-0.31
Fut. Price	0.09	0.39	0.22	0.29	0.82	0.36
Conv. Yield	-0.06	0.04	0.10	0.06	0.03	0.03
Cash Price	0.04	0.43	0.31	0.35	0.85	0.40
No Inventory-Demand Shocks						
Inventory-Supply Shocks from Production and China-Import Surprises Only						
Inventory	-0.19	0.12	0.56	0.89	0.58	0.39
Fut. Price	0.24	0.36	-0.03	-0.07	0.51	0.20
Conv. Yield	0.02	-0.01	-0.04	-0.08	-0.04	-0.03
Cash Price	0.26	0.36	-0.07	-0.14	0.47	0.18
No Inventory-Demand Shocks (95% confidence band)						
Inventory-Supply Shocks from Production and China-Import Surprises Only						
Inventory	-0.51 0.15	-0.34 0.70	-0.25 1.26	-0.21 1.55	-0.65 1.31	-0.32 0.94
Fut. Price	0.09 0.37	0.09 0.63	-0.35 0.34	-0.41 0.34	0.12 0.97	-0.07 0.52
Conv. Yield	-0.04 0.09	-0.09 0.07	-0.12 0.02	-0.20 0.01	-0.15 0.05	-0.09 0.03
Cash Price	0.07 0.43	0.04 0.67	-0.39 0.31	-0.47 0.27	0.10 0.94	-0.10 0.50
Identified Set						
No Inventory-Demand Shocks						
Inventory-Supply Shocks from Production and China-Import Surprises Only						
Inventory	-0.26 0.34	0.11 0.12	-0.06 0.65	0.30 0.99	0.21 0.68	0.18 0.43
Fut. Price	0.18 0.24	0.34 0.37	-0.02 0.02	-0.04 0.01	0.54 0.57	0.22 0.23
Conv. Yield	-0.05 0.02	-0.01 0.01	-0.05 0.05	-0.08 -0.03	-0.05 -0.02	-0.03 -0.01
Cash Price	0.13 0.27	0.34 0.37	-0.07 0.07	-0.12 -0.02	0.49 0.55	0.19 0.22
Identified Set (>95% confidence band)						
No Inventory-Demand Shocks						
Inventory-Supply Shocks from Production and China-Import Surprises Only						
Inventory	-0.57 0.68	-0.37 0.71	-0.88 1.34	-0.71 1.66	-0.92 1.40	-0.50 0.98
Fut. Price	0.03 0.37	0.10 0.64	-0.34 0.40	-0.40 0.42	0.14 1.02	-0.06 0.54
Conv. Yield	-0.11 0.09	-0.09 0.08	-0.13 0.12	-0.20 0.04	-0.15 0.05	-0.09 0.04
Cash Price	-0.06 0.44	0.05 0.67	-0.39 0.48	-0.46 0.39	0.11 1.01	-0.09 0.54
Production Surprises (MMT)						
Actual Prod.	87.0	72.9	80.7	91.5	90.7	
May Forecast	83.8	74.7	84.5	87.0	90.1	
Surprise	3.2	-1.8	-3.8	4.5	0.6	
China Import Surprises (MMT)						
Actual Imports	28.7	37.8	41.1	50.3	52.3	
May Forecast	31.5	34.5	35.5	38.1	49.0	
Surprise	-2.8	3.3	5.6	12.2	3.3	
Total Surprise	6.0	-5.2	-9.4	-7.7	-2.8	

Table S11. Log difference between actual and counterfactual for soybeans. Here we define the log cash price as log futures plus convenience yield. Table entries are results from the BAU calculations described in the text. Total surprise is production surprise minus China import surprise. Surprise terms divided by 6.6 MMT, which is average soybean inventory from 1996-2005. Because the identifying assumptions differ slightly, there is no requirement that the point identified parameters lie in the identified set.

Equation	*REA*	*Inventory*	*Futures*	*Conv. Yield*
	Reduced Form Estimates: $A^{-1}B_1$			
REA_{t-1}	0.59* (0.14)	-0.51* (0.19)	0.42* (0.15)	-0.02 (0.07)
$Inventory_{t-1}$	-0.05 (0.07)	0.71* (0.09)	0.07 (0.06)	0.04 (0.04)
$Futures_{t-1}$	-0.16 (0.10)	0.58* (0.17)	0.53* (0.10)	-0.05 (0.05)
$Conv.\ Yield_{t-1}$	0.01 (0.24)	-0.10 (0.38)	0.18 (0.28)	0.31* (0.13)
Constant	0.85 (0.77)	1.80 (1.11)	0.17 (0.69)	-0.21 (0.37)
Trend	-0.005 (0.004)	0.016* (0.006)	-0.014* (0.004)	0.001 (0.002)
	A Matrix: imposing $\alpha_{23} = 4.4 - 1/(\alpha_{32} + \alpha_{42}(1+\alpha_{34}))$			
REA	1	0	0	0
Inventory Supply	2.11	1	-3.44	-3.44
Inventory Demand	0.04	0.81	1	0.44
Supply of Storage	0.11	0.16	0	1
	A Matrix: Identified Set			
REA	1	0	0	0
Inventory Supply	[0.71, 1.25]	1	[-1.47,-0.25]	[-1.47,-0.25]
Inventory Demand	[-0.16,-0.04]	[0.48, 0.68]	1	[0.56, 0.64]
Supply of Storage	[0.09, 0.10]	[0.12, 0.14]	0	1
	A Matrix: >90% Confidence Interval			
REA	1	0	0	0
Inventory Supply	[0.42, 1.72]	1	[-1.82, -0.25]	[-1.82, -0.25]
Inventory Demand	[-0.29,0.15]	[0.35, 0.78]	1	[0.20, 0.95]
Supply of Storage	[0.02, 0.20]	[0.07, 0.20]	0	1

Table S12. Wheat VAR parameter estimates. Sample range: 1961–2005; standard errors in parentheses; *indicates significance at 5%; model selection criteria values are AICc=-648.86 and BIC=-620.31; for the two-lag model, we obtain AICc = -640.24 and BIC = -583.15, so the one-lag model is favored. We obtain the confidence intervals using a recursive-design wild bootstrap (1).

	2006-07	2007-08	2008-09	2009-10	2010-11	Average
No Inventory-Demand Shocks						
Inventory	0.00	0.00	0.00	0.00	0.00	0.00
Fut. Price	0.00	0.00	0.00	0.00	0.00	0.00
Conv. Yield	0.00	0.00	0.00	0.00	0.00	0.00
Cash Price	0.00	0.00	0.00	0.00	0.00	0.00
No Inventory-Demand or -Supply Shocks						
Inventory	-0.06	-0.28	0.21	0.60	0.34	0.16
Fut. Price	-0.01	0.52	0.07	-0.12	0.39	0.17
Conv. Yield	0.01	0.04	-0.04	-0.07	0.02	-0.01
Cash Price	0.00	0.56	0.03	-0.19	0.41	0.16
No Inventory-Demand Shocks						
Inventory-Supply Shocks from Production Surprises Only						
Inventory	-0.04	-0.23	0.18	0.52	0.27	0.14
Fut. Price	-0.03	0.48	0.09	-0.06	0.45	0.18
Conv. Yield	0.01	0.03	-0.03	-0.06	0.02	-0.01
Cash Price	-0.03	0.51	0.05	-0.12	0.47	0.18
No Inventory-Demand Shocks (95% confidence band)						
Inventory-Supply Shocks from Production Surprises Only						
Inventory	-0.23 0.17	-0.61 0.08	-0.40 0.60	-0.10 1.00	-0.41 0.86	-0.32 0.53
Fut. Price	-0.15 0.09	0.29 0.73	-0.17 0.45	-0.31 0.29	0.21 0.79	-0.01 0.46
Conv. Yield	-0.06 0.06	-0.05 0.11	-0.15 0.05	-0.17 0.02	-0.09 0.09	-0.10 0.06
Cash Price	-0.13 0.10	0.34 0.74	-0.18 0.38	-0.33 0.21	0.24 0.79	0.00 0.43
Identified Set						
No Inventory-Demand Shocks						
Inventory-Supply Shocks from Production Surprises Only						
Inventory	-0.02 0.02	-0.19 -0.13	0.14 0.16	0.30 0.43	-0.06 0.15	0.05 0.10
Fut. Price	-0.04 -0.04	0.46 0.46	0.10 0.11	-0.03 -0.01	0.50 0.53	0.20 0.21
Conv. Yield	0.00 0.00	0.02 0.02	-0.03 -0.02	-0.04 -0.02	0.03 0.04	0.00 0.00
Cash Price	-0.05 -0.04	0.48 0.49	0.07 0.09	-0.07 -0.02	0.53 0.58	0.20 0.22
Identified Set (>95% confidence band)						
No Inventory-Demand Shocks						
Inventory-Supply Shocks from Production Surprises Only						
Inventory	-0.21 0.22	-0.58 0.17	-0.47 0.58	-0.33 0.92	-0.73 0.76	-0.41 0.49
Fut. Price	-0.16 0.08	0.27 0.72	-0.16 0.48	-0.27 0.35	0.25 0.87	0.00 0.49
Conv. Yield	-0.07 0.06	-0.06 0.10	-0.14 0.07	-0.15 0.06	-0.08 0.11	-0.10 0.07
Cash Price	-0.15 0.09	0.31 0.72	-0.17 0.43	-0.29 0.30	0.29 0.88	0.02 0.47
Production Surprises (MMT)						
Actual Prod.	49.2	55.8	68.4	60.1	58.9	
May Forecast	51.0	59.2	65.1	55.1	55.6	
Surprise	-1.8	-3.3	3.3	5.0	3.3	

Table S13. Log difference between actual and counterfactual for wheat. Here we define the log cash price as log futures plus convenience yield. Table entries are results from the BAU calculations described in the text. Surprise terms divided by 18.7 MMT, which is average wheat inventory from 1996-2005. Because the identifying assumptions differ slightly, there is no requirement that the point identified parameters lie in the identified set.

Elasticity with Respect to	Crop Rotation		
	Corn-Corn	Corn-Other	Other-Other
Price of Corn	1.644**	0.179**	-1.315**
	(0.198)	(0.065)	(0.238)
Price of Other Crops	-1.314**	-0.221**	0.891**
	(0.261)	(0.085)	(0.215)

Table S14. Long-Run Crop Rotation Elasticities. Bootstrap standard errors are in parentheses. Note * and ** denote significance at the 10% and 5% levels, respectively. The results in this table are replicated from results in table A10 in the supplementary appendix of Pates and Hendricks(17).

Elasticity with Respect to	Corn Area Elasticity
Price of Corn	0.574**<br>(0.045)
Price of Other Crops	-0.467**<br>(0.062)

Table S15. Long-Run Aggregate Corn Acreage Elasticities. Bootstrap standard errors are in parentheses. Note * and ** denote significance at the 10% and 5% levels, respectively. The results in this table are replicated from results in table 3 of Pates and Hendricks (17).

Rotation	NO_3 Leaching [kg/ha]	P runoff [kg/ha]	Sediment loss [tons/km^2]
CC = continuous corn	41.6 ± 27.5	0.282 ± 0.279	22.2 ± 35.0
SS = continuous soy	15.8 ± 12.4	0.179 ± 0.224	14.2 ± 22.5
CS = corn-soy rotation	28.8 ± 19.5	0.231 ± 0.250	18.2 ± 28.8
WW = continuous wheat	17.0 ± 11.3	0.202 ± 0.272	13.0 ± 20.6
CW = corn-wheat rotation	29.4 ± 18.8	0.242 ± 0.272	17.6 ± 27.8

Table S16. Average field-level water quality impacts (+/- one standard deviation) across CONUS cropland over 2007-16 time period for different cropping rotations.

Rotation	Fertilizer N [kg/ha]	Fertilizer P [kg/ha]	Manure N [kg/ha]	Manure P [kg/ha]
CC = continuous corn	174 ± 49.9	17.9 ± 6.50	18.9 ± 36.7	5.60 ± 11.5
SS = continuous soy	0 ± 0	11.2 ± 4.09	18.9 ± 36.7	5.60 ± 11.5
CS = corn-soy rotation	86.8 ± 25.0	14.5 ± 6.38	18.9 ± 36.7	5.60 ± 11.5
WW = continuous wheat	68.9 ± 25.4	7.82 ± 2.88	18.9 ± 36.7	5.60 ± 11.5
CW = corn-wheat rotation	121 ± 65.7	12.8 ± 7.10	18.9 ± 36.7	5.60 ± 11.5

Table S17: Fertilizer and manure inputs to each cropping rotation. Average (+/- one standard deviation) fertilizer and manure nitrogen and phosphorus across CONUS cropland.

CC compared to XX	NO_3 Leaching [kg/ha]	P runoff [kg/ha]	Sediment loss [tons/km^2]
CC–SS	25.8 ± 17.5	0.102 ± 0.080	8.01 ± 12.51
CC–CS	12.9 ± 8.8	0.051 ± 0.040	4.00 ± 6.25
CC–WW	24.6 ± 18.5	0.080 ± 0.099	9.20 ± 14.51
CC–CW	12.2 ± 9.34	0.039 ± 0.051	4.60 ± 7.25

Table S18: Corn intensification impact. Average (+/- one standard deviation) difference of impacts due to continuous corn to impacts from other cropping rotations, i.e. the differential impact of continuous corn compared to the other rotations.

Elasticity with Respect to	Type of Transition			
	Expand Cropland from Pasture	Abandon Cropland to Pasture	Expand Cropland from CRP	Abandon Cropland to CRP
Price of Crops	-0.015<br>(0.073)	0.014<br>(0.053)	7.350**<br>(0.323)	-1.218**<br>(0.096)
Pasture Rent	-0.298**<br>(0.079)	-0.685**<br>(0.050)		
CRP Rent			-0.394**<br>(0.129)	0.227**<br>(0.091)

Table S19. Five-Year Cropland Transition Elasticities. Bootstrap standard errors are in parentheses. Note * and ** denote significance at the 10% and 5% levels, respectively.

Elasticity with Respect to	Cropland Area Elasticity
Price of Crops	0.071**<br>(0.005)
Pasture Rent	0.024**<br>(0.004)
CRP Rent	-0.005**<br>(0.001)

Table S20. Five-Year Aggregate Cropland Area Elasticities. Bootstrap standard errors are in parentheses. Note * and ** denote significance at the 10% and 5% levels, respectively.

Expand Cropland from Pasture or CRP			Abandon Cropland to Pasture or CRP				
Region	Change (ha)		Region	Change (ha)		Net Change	
F	673,049	**	F	47,678		625,371	**
	(52,699)			(41,453)		(67,061)	
H	314,044	**	H	-62,641	*	376,685	**
	(40,129)			(33,776)		(52,391)	
JNOP	-41,023		JNOP	19,443		-60,465	
	(64,234)			(31,389)		(72,313)	
KL	-16,834		KL	-82,820	*	65,987	
	(56,325)			(44,700)		(71,843)	
Mcrop	95,368	**	Mcrop	-8,917		104,285	*
	(39,825)			(45,072)		(59,688)	
Mgrass	759,093	**	Mgrass	-257,218	**	1,016,311	**
	(97,651)			(76,002)		(125,091)	
RST	11,969		RST	-9,707		21,677	
	(20,716)			(18,899)		(28,194)	
Total	1,795,668	**		-354,183	**	2,149,851	**
	(151,295)			(119,121)		(193,298)	

Table S21. Predicted changes in transitions of cropland with pasture or CRP due to RFS. Bootstrap standard errors are in parentheses. Note * and ** denote significance at the 10% and 5% levels, respectively.

Expand Cropland from Pasture			Abandon Cropland to Pasture				
Region	Change (ha)		Region	Change (ha)		Net Change	
F	33,532		F	98,394	**	-64,862	
	(27,503)			(38,556)		(47,251)	
H	-9,347		H	35,468		-44,814	
	(31,021)			(32,399)		(44,421)	
JNOP	-116,488	*	JNOP	45,590		-162,079	**
	(63,763)			(30,692)		(71,323)	
KL	-68,019		KL	-74,499	*	6,480	
	(55,507)			(44,643)		(70,844)	
Mcrop	15,464		Mcrop	29,689		-14,225	
	(38,490)			(41,713)		(55,925)	
Mgrass	124,978		Mgrass	-206,332	**	331,310	**
	(93,895)			(74,291)		(120,494)	
RST	301		RST	-5,303		5,604	
	(20,612)			(18,741)		(27,816)	
Total	-19,580			-76,993		57,413	
	(138,067)			(114,138)		(178,807)	

Table S22. Predicted changes in transitions of cropland with pasture due to the RFS. Bootstrap standard errors are in parentheses. Note * and ** denote significance at the 10% and 5% levels, respectively.

Expand Cropland from CRP			Abandon Cropland to CRP				
Region	Change (ha)		Region	Change (ha)		Net Change	
F	639,518	**	F	-50,715	**	690,233	**
	(45,751)			(14,671)		(47,816)	
H	323,391	**	H	-98,109	**	421,500	**
	(26,084)			(9,258)		(27,738)	
JNOP	75,466	**	JNOP	-26,148	**	101,613	**
	(9,560)			(6,473)		(11,300)	
KL	51,186	**	KL	-8,321	**	59,507	**
	(9,827)			(3,707)		(10,630)	
Mcrop	79,904	**	Mcrop	-38,606	**	118,510	**
	(10,065)			(16,468)		(19,416)	
Mgrass	634,115	**	Mgrass	-50,887	**	685,002	**
	(28,819)			(14,127)		(31,993)	
RST	11,669	**	RST	-4,404	**	16,073	**
	(4,116)			(2,334)		(4,670)	
Total	1,815,248	**		-277,190	**	2,092,438	**
	(61,823)			(29,175)		(68,261)	

Table S23. Predicted changes in transitions of cropland with CRP due to RFS. Bootstrap standard errors are in parentheses. Note * and ** denote significance at the 10% and 5% levels, respectively.

SI References

1. C. A. Carter, G. C. Rausser, A. Smith, Commodity storage and the market effects of biofuel policies. *American Journal of Agricultural Economics* **99**, 1027–1055 (2017).

2. J. C. Williams, B. D. Wright, *Storage and commodity markets.* (Cambridge university press, 1991).

3. C. A. Carter, G. C. Rausser, A. Smith, Commodity Booms and Busts. *Annual Review of Resource Economics* **3**, 87–118 (2011).

4. M. K. Adjemian, A. Smith, Using USDA Forecasts to Estimate the Price Flexibility of Demand for Agricultural Commodities. *American Journal of Agricultural Economics* **94**, 978–995 (2012).

5. N. P. Hendricks, A. Smith, D. A. Sumner, Crop Supply Dynamics and the Illusion of Partial Adjustment. *Am J Agric Econ* **96**, 1469–1491 (2014).

6. L. Kilian, Not All Oil Price Shocks Are Alike: Disentangling Demand and Supply Shocks in the Crude Oil Market. *American Economic Review* **99**, 1053–1069 (2009).

7. S. Gonçalves, L. Kilian, Bootstrapping autoregressions with conditional heteroskedasticity of unknown form. *Journal of Econometrics* **123**, 89–120 (2004).

8. P. Garcia, S. H. Irwin, A. Smith, Futures Market Failure? *Am J Agric Econ* **97**, 40–64 (2015).

9. N. P. Hendricks, *et al.*, The environmental effects of crop price increases: Nitrogen losses in the US Corn Belt. *Journal of Environmental Economics and Management* **68**, 507–526 (2014).

10. J. Woodard, Big data and Ag-Analytics: An open source, open data platform for agricultural & environmental finance, insurance, and risk. *Agricultural Finance Review* **76**, 15–26 (2016).

11. F. S. A. USDA, FSA Common Land Unit infosheet (2012) (July 15, 2015).

12. L. Yan, D. P. Roy, Automated crop field extraction from multi-temporal Web Enabled Landsat Data. *Remote Sensing of Environment* **144**, 42–64 (2014).

13. C. Boryan, Z. Yang, R. Mueller, M. Craig, Monitoring US agriculture: the US Department of Agriculture, National Agricultural Statistics Service, Cropland Data Layer Program. *Geocarto International* **26**, 341–358 (2011).

14. N. R. C. S. Soil Survey Staff United States Department of Agriculture, Soil Survey Geographic (SSURGO) Database for the United States (November 15, 2018).

15. C. Daly, *et al.*, High-quality spatial climate data sets for the United States and beyond. *Transactions of the ASAE* **43**, 1957 (2000).

16. Bloomberg L.P., “Local US agricultural spot and commodity futures prices.” (November 8, 2017).

17. N. J. Pates, N. P. Hendricks, Fields from Afar: Evidence of Heterogeneity in United States Corn Rotational Response from Remote Sensing Data. *American Journal of Agricultural Economics* **n/a** (2021).

18. USDA, “2012 National Resources Inventory: Summary Report” (Natural Resources Conservation Service , 2015).

19. E. R. S. USDA, “USDA ERS - Agricultural Baseline Database” (May 21, 2020).

20. E. R. S. USDA, “USDA ERS - Commodity Costs and Returns” (May 21, 2020).

21. D. Hofstrand, W. Edwards, “Computing a Pasture Rental Rate” (2015) (June 10, 2020).

22. J. Atwood, T. Watts, K. Price, J. Kastens, The big picture - Satellite remote sensing applications in rangeland assessment and crop insurance in (2005) (May 21, 2020).

23. USDA NASS, QuickStats (2012) (May 10, 2012).

24. M. Stubbs, Conservation Reserve Program (CRP): Status and Issues. *Congressional Research Service*, 7–5700 (2012).

25. N. P. Hendricks, Potential Benefits from Innovations to Reduce Heat and Water Stress in Agriculture. *Journal of the Association of Environmental and Resource Economists* **5**, 545–576 (2018).

26. R. N. Lubowski, A. J. Plantinga, R. N. Stavins, What drives land-use change in the United States? A national analysis of landowner decisions. *Land Economics* **84**, 529–550 (2008).

27. B. S. Rashford, J. A. Walker, C. T. Bastian, Economics of grassland conversion to cropland in the Prairie Pothole Region. *Conservation Biology* **25**, 276–284 (2011).

28. J. J. Lawler, *et al.*, Projected land-use change impacts on ecosystem services in the United States. *Proceedings of the National Academy of Sciences* **111**, 7492–7497 (2014).

29. C. Langpap, J. Wu, Potential Environmental Impacts of Increased Reliance on Corn-Based Bioenergy. *Environmental and Resource Economics* **49**, 147–171 (2011).

30. R. Claassen, C. Langpap, J. Wu, Impacts of Federal Crop Insurance on Land Use and Environmental Quality. *American Journal of Agricultural Economics* **83**, aaw075 (2016).

31. J. M. Wooldridge, *Econometric analysis of cross section and panel data* (MIT press, 2010).

32. T. J. Lark, J. M. Salmon, H. K. Gibbs, Cropland expansion outpaces agricultural and biofuel policies in the United States. *Environ. Res. Lett.* **10**, 044003 (2015).

33. T. J. Lark, R. M. Mueller, D. M. Johnson, H. K. Gibbs, Measuring land-use and land-cover change using the U.S. department of agriculture's cropland data layer: Cautions and recommendations. *International Journal of Applied Earth Observation and Geoinformation* **62**, 224–235 (2017).

34. T. Lark, M. Bougie, S. Spawn, H. Gibbs, "Cropland Expansion in the United States, 2008-2016" (University of Wisconsin-Madison, 2018).

35. M. Motew, *et al.*, The Influence of Legacy P on Lake Water Quality in a Midwestern Agricultural Watershed. *Ecosystems* **20**, 1468–1482 (2017).

36. S. D. Donner, C. J. Kucharik, Corn-based ethanol production compromises goal of reducing nitrogen export by the Mississippi River. *Proceedings of the National Academy of Sciences* **105**, 4513–4518 (2008).

37. T. Lark, S. Spawn, M. Bougie, H. Gibbs, Cropland expansion in the United States produces marginal yields at high costs to wildlife. *Nature Communications* (Accepted, in press).

38. N. W. Chaney, *et al.*, POLARIS Soil Properties: 30-m Probabilistic Maps of Soil Properties Over the Contiguous United States. *Water Resour Res* **55**, 2916–2938 (2019).

39. E. Benham, R. J. Ahrens, W. D. Nettleton, "Clarification of Soil Textural Class Boundaries" (U.S. Department of Agriculture, Natural Resources Conservation Service, National Soil Survey Center, 2009).

40. , "Elevation derivatives for national applications" (2005) https:/doi.org/10.3133/fs20053049.

41. L. W. Zevenbergen, C. R. Thorne, Quantitative-Analysis of Land Surface-Topography. *Earth Surf Processes* **12**, 47–56 (1987).

42. P. Panagos, P. Borrelli, K. Meusburger, New European Slope Length and Steepness Factor (LS-Factor) for Modeling Soil Erosion by Water. *Geosciences* **5**, 117–126 (2015).

43. T. G. Freeman, Calculating Catchment-Area with Divergent Flow Based on a Regular Grid. *Comput Geosci* **17**, 413–422 (1991).

44. P. Quinn, K. Beven, P. Chevallier, O. Planchon, The Prediction of Hillslope Flow Paths for Distributed Hydrological Modeling Using Digital Terrain Models. *Hydrol Process* **5**, 59–79 (1991).

45. P. J. J. Desmet, G. Govers, A GIS procedure for automatically calculating the USLE LS factor on topographically complex landscape units. *J Soil Water Conserv* **51**, 427–433 (1996).

46. V. Olaya, “Basic land-surface parameters” in *Geomorphometry: Concepts, Software, Applications. Developments in Soil Science, 33.*, T. Hengl, H. I. Reuter, Eds. (Elsevier, 2009), pp. 141–169.

47. L. McKay, *et al.*, NHDPlus Version 2: User Guide (2012).

48. J. R. Williams, “Sediment-yield prediction with Universal Equation using runoff energy factor” in *Present and Prospective Technology for Predicting Sediment Yield and Sources. Vol. ARS-S-40.*, (U.S. Department of Agriculture, Agricultural Research Service, 1975), pp. 244–252.

49. M. Haines, P. Fishback, P. Rhode, United States Agriculture Data, 1840 - 2012 (2018) https:/doi.org/10.3886/ICPSR35206.v4.

50. S. Manson, J. Schroeder, D. V. Riper, S. Ruggles, IPUMS National Historical Geographic Information System: Version 13.0 (2018) https:/doi.org/10.18128/D050.V13.0.

51. N. Ramankutty, J. A. Foley, Estimating historical changes in global land cover: Croplands from 1700 to 1992. *Global Biogeochem Cy* **13**, 997–1027 (1999).

52. J. Fry, *et al.*, Completion of the 2006 National Land Cover Database for the Conterminous United States. *Photogrammetric Engineering and Remote Sensing* **77**, 858–864 (2011).

53. C. Homer, *et al.*, Completion of the 2001 National Land Cover Database for the conterminous United States. *Photogramm Eng Rem S* **73**, 337–341 (2007).

54. C. Homer, *et al.*, Completion of the 2011 National Land Cover Database for the conterminous United States–representing a decade of land cover change information. *Photogrammetric Engineering & Remote Sensing* **81**, 345–354 (2015).

55. T. Sohl, *et al.*, Modeled historical land use and land cover for the conterminous United States. *J Land Use Sci* **11**, 476–499 (2016).

56. T. L. Sohl, *et al.*, Spatially explicit modeling of 1992–2100 land cover and forest stand age for the conterminous United States. *Ecological Applications* **24**, 1015–1036 (2014).

57. Q. F. Hamlin, *et al.*, Quantifying Landscape Nutrient Inputs With Spatially Explicit Nutrient Source Estimate Maps. *Journal of Geophysical Research: Biogeosciences* **125**, e2019JG005134 (2020).

58. R. B. Alexander, R. A. Smith, "County-Level Estimates of Nitrogen and Phosphorus Fertilizer Use in the United States, 1945 to 1985. Open-File Report 90-130" (U.S. Geological Survey, 1990).

59. J. W. Brakebill, J. A. M. Gronberg, County-Level Estimates of Nitrogen and Phosphorus from Commercial Fertilizer for the Conterminous United States, 1987-2012 (2017) https:/doi.org/10.5066/F7H41PKX (February 27, 2019).

60. J. A. M. Gronberg, N. E. Spahr, "County-Level Estimates of Nitrogen and Phosphorus from Commercial Fertilizer for the Conterminous United States, 1987–2006. Scientific Investigations Report 2012-5207" (U.S. Geological Survey, 2012).

61. J. M. Gronberg, T. L. Arnold, "County-level estimates of nitrogen and phosphorus from animal manure for the conterminous United States, 2007 and 2012" (US Geological Survey, 2017).

62. D. K. Mueller, J. A. M. Gronberg, "County-Level Estimates of Nitrogen and Phosphorus from Animal Manure for the Conterminous United States, 2002: U.S. Geological Survey Open-File Report 2013-1065" (2013).

63. B. C. Ruddy, D. L. Lorenz, D. K. Mueller, "County-Level Estimates of Nutrient Inputs to the Land Surface of the Conterminous United States, 1982–2001: U.S. Geological Survey Scientific Investigations Report 2006-5012" (2006).

64. R. Mylavarapu, D. Wright, G. Kidder, "UF/IFAS Standardized Fertilization Recommendations for Agronomic Crops. SL129." (University of Florida, Soil and Water Science Department, Institute of Food and Agricultural Sciences, 2015).

65. C. A. M. Laboski, J. B. Peters, "Nutrient application guidelines for field, vegetable, and fruit crops in Wisconsin, A2809 R-11-2012" (University of Wisconsin Extension, 2012).

66. J. F. Brown, M. S. Pervez, Merging remote sensing data and national agricultural statistics to model change in irrigated agriculture. *Agricultural Systems* **127**, 28–40 (2014).

67. J. S. Gerber, *et al.*, Spatially explicit estimates of N2O emissions from croplands suggest climate mitigation opportunities from improved fertilizer management. *Global Change Biology* **22**, 3383–3394 (2016).

68. , "IPCC Assessment Report 5: Anthropogenic and Natural Radiative Forcing" (Cambridge University Press, 2013).

69. S. A. Spawn, T. J. Lark, H. K. Gibbs, Carbon emissions from cropland expansion in the United States. *Environmental Research Letters* **14**, 045009 (2019).

70. J. Sanderman, Soil carbon profile data from paired land use comparisons (2017) https:/doi.org/10.7910/DVN/QQQM8V (July 9, 2020).

71. I. Gelfand, *et al.*, Carbon debt of Conservation Reserve Program (CRP) grasslands converted to bioenergy production. *Proceedings of the National Academy of Sciences* **108**, 13864–13869 (2011).

72. C. Poeplau, *et al.*, Temporal dynamics of soil organic carbon after land-use change in the temperate zone – carbon response functions as a model approach. *Global Change Biology* **17**, 2415–2427 (2011).

73. U.S. EPA, "Renewable Fuel Standard Program (RFS2) Regulatory Impact Analysis" (Office of Transportation and Air Quality, Assessment and Standards Division, 2010).

74. M. D. Webb, "Reworking Wild Bootstrap Based Inference for Clustered Errors" (Queen's Economics Department Working Paper, 2013) (July 8, 2020).

75. Y. Li, R. Miao, M. Khanna, Effects of Ethanol Plant Proximity and Crop Prices on Land-Use Change in the United States. *Am J Agric Econ* **101**, 467–491 (2019).

76. S. Ahmed, T. Hertel, R. Lubowski, "Calibration of a Land Cover Supply Function Using Transition Probabilities" (Center for Global Trade Analysis, Department of Agricultural Economics, Purdue University, 2009) (July 14, 2021).

77. K. J. Barr, B. A. Babcock, M. A. Carriquiry, A. M. Nassar, L. Harfuch, Agricultural Land Elasticities in the United States and Brazil. *Appl Econ Perspect Policy* **33**, 449–462 (2011).

78. N. P. Hendricks, E. Er, Changes in cropland area in the United States and the role of CRP. *Food Policy* **75**, 15–23 (2018).

79. J. T. Crawford, E. H. Stanley, Controls on methane concentrations and fluxes in streams draining human-dominated landscapes. *Ecological Applications* **26**, 1581–1591 (2016).

80. J. J. Beaulieu, T. DelSontro, J. A. Downing, Eutrophication will increase methane emissions from lakes and impoundments during the 21st century. *Nat Commun* **10**, 1375 (2019).

81. Y. Yao, *et al.*, Increased global nitrous oxide emissions from streams and rivers in the Anthropocene. *Nat. Clim. Chang.* **10**, 138–142 (2020).

82. K. Paustian, *et al.*, Climate-smart soils. *Nature* **532**, 49–57 (2016).

83. N. USDA, “2017 Census of Agriculture” (2019) (September 19, 2019).

84. T. Wade, R. Claassen, S. Wallander, “Conservation-practice adoption rates vary widely by crop and region” (2015).

85. D. S. Powlson, *et al.*, Limited potential of no-till agriculture for climate change mitigation. *Nature Climate Change* **4**, 678–683 (2014).

86. R. T. Conant, M. Easter, K. Paustian, A. Swan, S. Williams, Impacts of periodic tillage on soil C stocks: A synthesis. *Soil and Tillage Research* **95**, 1–10 (2007).

87. California Air Resources Board, CA-GREET3.0 Supplemental Document and Tables of Changes (2018).

88. Wang, Michael, *et al.*, *Greenhouse gases, Regulated Emissions, and Energy use in Technologies Model ® (2020 .Net)* (Argonne National Laboratory (ANL), Argonne, IL (United States), 2020) https:/doi.org/10.11578/GREET-NET-2020/DC.20200913.1 (July 26, 2021).

89. H. Kwon, *et al.*, “Carbon Calculator for Land Use and Land Management Change from Biofuels Production (CCLUB)” (Argonne National Lab.(ANL), Argonne, IL (United States), 2020).

90. M. Wang, *et al.*, “Summary of Expansions and Updates in GREET® 2020” (Argonne National Lab.(ANL), Argonne, IL (United States), 2020).

91. C. Malins, R. Plevin, R. Edwards, How robust are reductions in modeled estimates from GTAP-BIO of the indirect land use change induced by conventional biofuels? *Journal of Cleaner Production* **258**, 120716 (2020).

92. S. A. Spawn-Lee, *et al.*, Comment on ‘Carbon intensity of corn ethanol in the United States: state of the science’ (2021) https:/doi.org/10.32942/osf.io/cxhz5 (July 5, 2021).

93. T. D. Searchinger, S. Wirsenius, T. Beringer, P. Dumas, Assessing the efficiency of changes in land use for mitigating climate change. *Nature* **564**, 249–253 (2018).

94. T. Searchinger, R. Edwards, D. Mulligan, R. Heimlich, R. Plevin, Do biofuel policies seek to cut emissions by cutting food? *Science* **347**, 1420–1422 (2015).
